# Immunophenotypic and structural signatures of severe granulation tissue in airway implant patients

**DOI:** 10.1186/s12931-025-03480-7

**Published:** 2026-01-07

**Authors:** Kimberly Barbet, Birte Schwarz, Anja Lena Thiebes, Anja Elisabeth Luengen, Christian Taube, Michaela Schedel, Kaid Darwiche

**Affiliations:** 1 Department of Pulmonary Medicine, University Medical Center Essen - Ruhrlandklinik, Essen, Germany; 2 Interventional Pulmonology, Department of Pulmonary Medicine, University Medical Center Essen - Ruhrlandklinik, Essen, Germany; 3 Department of Biohybrid and Medical Textiles (BioTex), AME - Institute of Applied Medical Engineering, Helmholtz Institute, RWTH Aachen University, Aachen, Germany; 4 Department of Pulmonology, University Medical Center Essen, Essen, Germany; 5 Department of Translational Pulmonology, Department of Pulmonary Medicine, University Medical Center Essen- Ruhrlandklinik, Essen, Germany

**Keywords:** Granulation tissue, Endobronchial valve, Immunophenotyping, Biopsy, COPD

## Abstract

**Background:**

Granulation tissue formation is a frequent complication following airway implant procedures such as endobronchial valve (EBV) therapy in patients with chronic obstructive pulmonary disease (COPD). This hyperplastic response can impair implant function and clinical outcomes, yet the underlying immunological mechanisms remain poorly understood.

**Methods:**

In a cohort of EBV patients (*n* = 147), we developed an optical scaling and scoring system to classify the severity of granulation tissue in relation to clinical outcomes. Based on this system, we prospectively collected endobronchial biopsies from EBV (*n* = 41) and stent patients (*n* = 11) for immune and structural profiling across different severity grades. Granulation tissue was analysed by histology, immunofluorescence, and flow cytometry.

**Results:**

Using the optical scoring system, almost 75% of EBV patients developed hyperplastic granulation tissue (34.7% moderate, 38.8% severe). In many moderate to severe cases, valve dysfunction, dislocation, and the need for revision was observed. Increasing severity of granulation tissue was associated with profound extracellular matrix (ECM) disruption and increased immune cell infiltration. Immunophenotyping revealed elevated neutrophils and regulatory T cells (Tregs) with a central memory phenotype. Macrophages in patients with severe granulation tissue formation showed a shift toward a CD163^+^ M2-like polarisation. Structural analyses demonstrated a loss of epithelial integrity and an upregulation of ECM remodelling markers, fibronectin, and tenascin C.

**Conclusion:**

This study provides the first systematic immune-morphological classification of airway granulation tissue following EBV therapy. Our findings revealed distinct immune and ECM signatures associated with clinical complications identify potential mechanisms and therapeutic targets, particularly relevant for COPD patients in whom current anti-inflammatory strategies are insufficient.

**Graphical abstract:**

**Figure:**
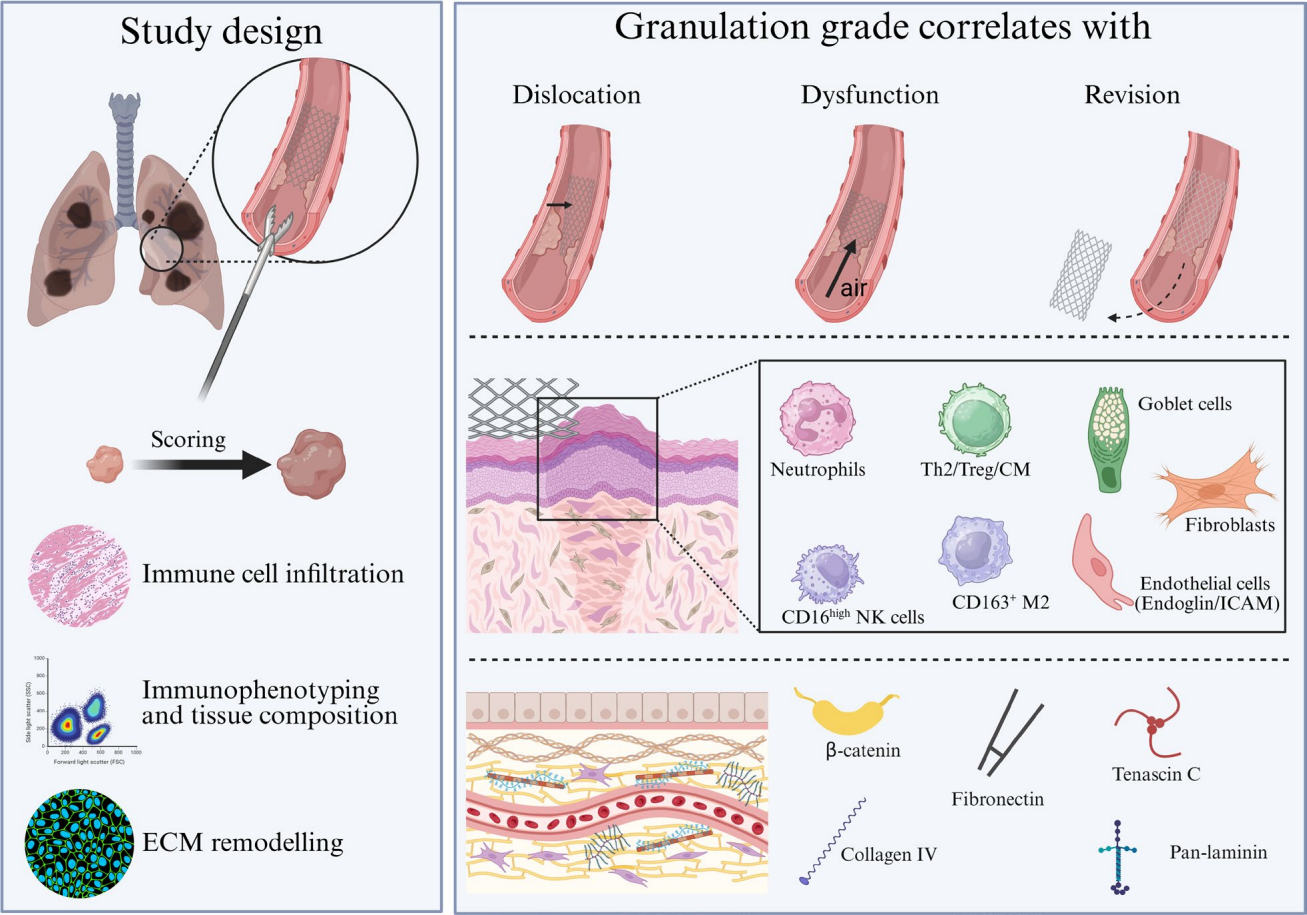
Overview of the study design and main findings on the immunological and structural characterisation of granulation tissue following endobronchial valve (EBV) or stent implantation. Granulation tissue was obtained by forceps biopsy during bronchoscopy. Scoring was performed according to severity, followed by histological and immunofluorescence analysis for immune cell infiltration, immunophenotyping by flow cytometry, and assessment of extracellular matrix (ECM) remodelling. Increased granulation grade was associated with clinical complications including EBV dislocation, dysfunction, and need for revision procedures. Key immune cell types [neutrophils, type 2 T helper (Th2) cells, regulatory T cells (Treg), central memory cells (CM), CD16high natural killer cells (NK), CD163^+^ macrophages], goblet cells, fibroblasts, endothelial cells (endoglin, ICAM), and ECM components (β-Catenin, fibronectin, collagen IV, tenascin C, pan-laminin).

**Supplementary Information:**

The online version contains supplementary material available at 10.1186/s12931-025-03480-7.

## Introduction

Over the past two decades, interventional pulmonology has developed various implantation techniques, including stents and valves, to treat collapsed airways and airway stenosis. Stents are used for the reopening of airways obstructed by strictures, tumour invasion or trauma [1]. In contrast, endobronchial valves (EBVs) are inserted to deflate areas of the lung that are overinflated affected by emphysematous hyperinflation. This is often the case in patients with chronic obstructive pulmonary disease (COPD) and emphysema [2–5]. Implanting EBVs into the affected lung lobe leads to complete occlusion, which results in atelectasis and thereby reducing lung hyperinflation [2]. With ongoing technical advances, new implant devices will continue to increase the number of patients successfully treated for a pulmonary disease.

One of the most common side effects after implantation of EBVs or stents is the formation of hyperplastic granulation tissue due to excessive cell growth [2, 6–8]. As a consequence, bleeding may occur and the overall treatment fails to be successful due to the impaired function of the implant, requiring repeated interventions [9]. In some cases, it leads to hemoptysis and persistent cough [9]. Recent reviews have emphasised that granulation tissue formation represents a key limitation to the long-term success of implantable lung devices and remains a major obstacle in interventional pulmonology [10] for which there is currently no therapeutic solution.

Despite its clinical relevance, little is known about the incidence, risk factors, or underlying mechanistic pathophysiology of granulation tissue. The only two published studies to date, which found granulation tissue in 40–50% of patients, included patients requiring the valves to be removed [2, 9]. Clinical observations suggest considerable inter-individual variability in granulation tissue development. In addition to inflammatory triggers, mechanical stimuli such as friction and pressure, bacterial colonisation, noxious agents, or endogenous factors have been proposed as potential contributors [11]. Other risk factors may include impaired mucociliary transport and microcirculation, leading to tissue starvation [12, 13]. Clinically, at this time no reliable parameters exist to identify patients who are at a higher risk of developing granulation tissue. To the best of our knowledge, the pathophysiology of implant-associated granulation tissue has not been investigated and the underlying mechanism(s) remains poorly understood.

A better understanding of the immunological and structural components of airway granulation tissue is essential for the development of preventive or therapeutic strategies. In the current study, we investigated a cohort of patients with COPD after valve placement and patients with benign or malignant airway stenosis after stent placement. A scoring system was developed to classify the degree of granulation tissue formation. Based on this classification, the prevalence of patients without or with moderate to severe granulation tissue was analysed in a larger cohort. We hypothesised that discrete immune and mesenchymal cell populations orchestrate a fibrosis-like remodelling response of implant-associated granulation tissue. We therefore aimed to delineate, for the first time, the immunological and structural architecture of bronchoscopic granulation tissue biopsies.

## Materials and methods

### Optical scaling and scoring

**Fig. 1:**
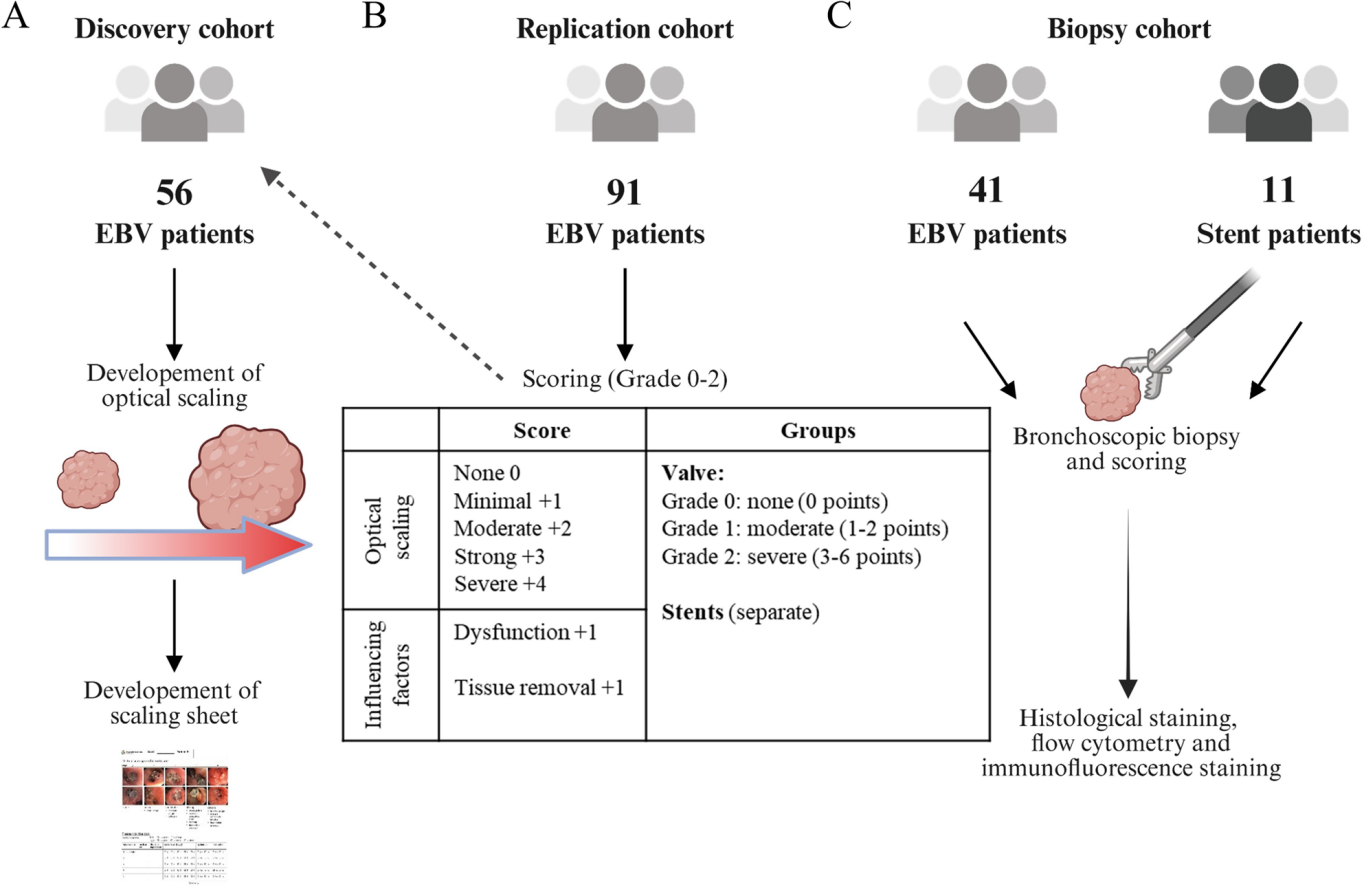
Study design.** A** Retrospective analysis in a discovery cohort (*n* = 56) of endobronchial valve (EBV) patients to develop an optical scaling and scaling sheet. **B** Categorisation based on the scaling sheet in an independent replication cohort (= 91) and re-categorisation of discovery cohort. **C** Prospective analysis of bronchoscopic biopsies from EBV patients EBV (*n* = 41) or stent (*n* = 11) grouped by the advanced score to assess the degree of granulation

To quantify the extent of granulation tissue in patients with COPD following EBV implantation, we conducted a retrospective analysis of 56 patients (discovery cohort, Figs. 1a + 2a) who had undergone follow-up bronchoscopy in 2022 after endobronchial volume reduction. For this cohort, only patients were included who had undergone EBV implantation at least 1 month prior to inclusion with available images of granulation tissue or valves without granulation tissue documented during bronchoscopy documentation. Patients with previous EBV implantation were excluded if valve images were either not existent or of insufficient quality. In accordance to the current clinical practice in interventional pulmonology for visual evaluation of granulation tissue, predefined morphological criteria were applied including size of the polyps (small, moderate, strong, massive), bleeding, necessary interventions (supplementary Fig. 1). Assessment was validated through inter-observer agreement of two independent trained observers. Using this cohort, we developed a visual scaling system to classify the amount of granulation tissue near the valves. Granulation tissue was categorised into five groups: none, mild, moderate, strong, and severe. For the assessment of inter-rater reliability, all images were scored by two independent evaluators using the same predefined optical scale.

**Fig. 2:**
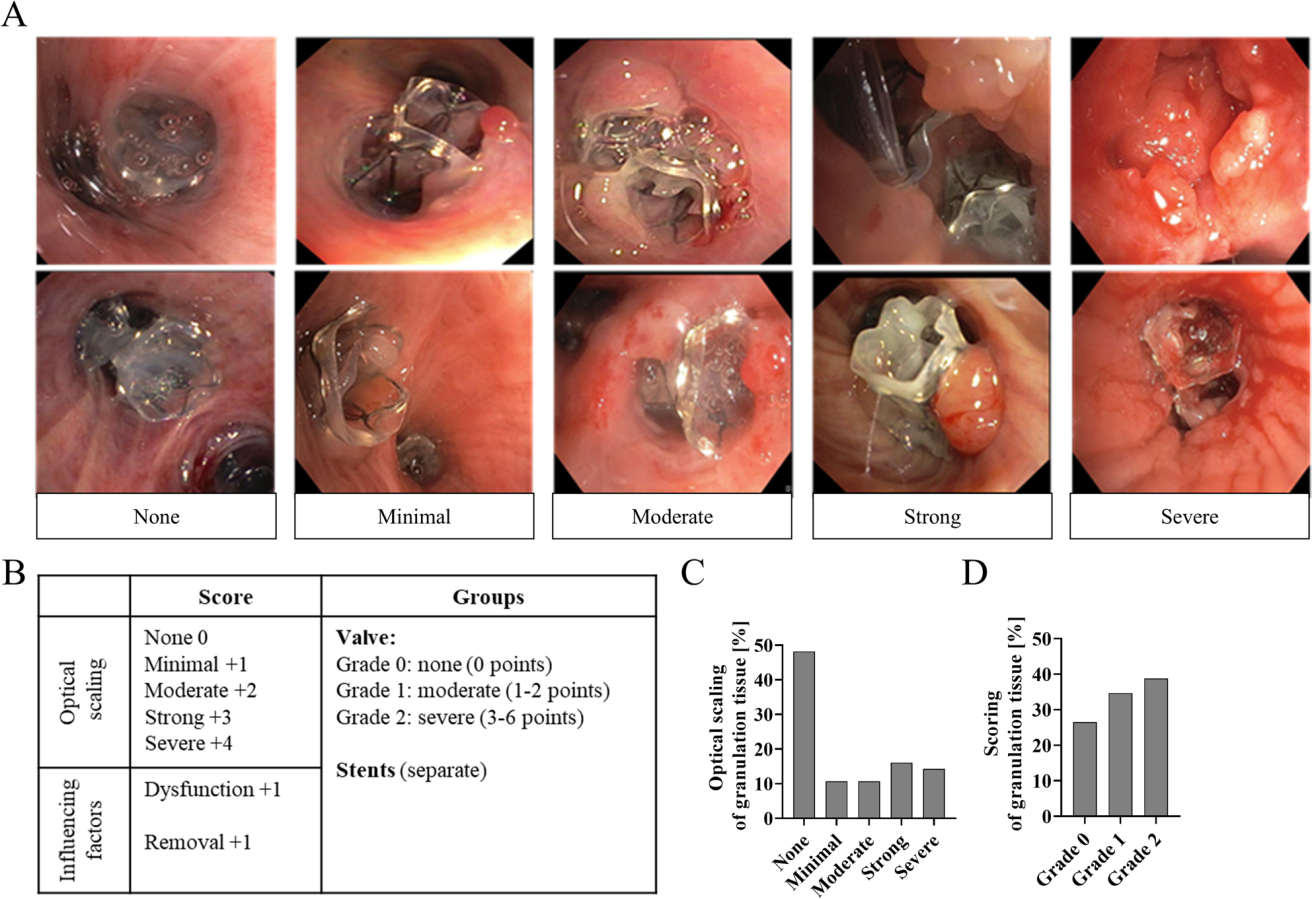
Scoring system of EBV patients by optical scaling including other risk factors.** A** The visual scaling has been developed from the discovery cohort and representative examples for each granulation tissue degree are shown. The groups were originally divided into none, minimal, moderate, strong, or severe granulation tissue. **B** Then, a categorisation was developed for EBV patients including additional influencing factors: Grade 0 = none (0 points), Grade 1 = moderate (1–2 points), Grade 2 = severe (3–6 points). **C** Relative distribution of optical scaling of granulation tissue after valve implantation of the discovery cohort (*n* = 56). **D** Relative distribution of granulation tissue degree after categorizing the discovery and the replication cohort (Grades 0–2, *n* = 147)

Based on this optical evaluation, a scaling sheet was created (see supplementary Fig. 2), which included additional factors such as the affected lung lobe, dislocation of the valves, valve dysfunction, the need for valve revision, and other local conditions (e.g., polyp formation, bleeding). The assessment led to a score of minimal 0 to a maximal score of 6 points (Figs. 1b + 2b). This initial scaling was performed independently by different trained physicians during bronchoscopy at the University Hospital Essen – Ruhrlandklinik (total *n* = 91, replication cohort) in 2023. For this replication cohort, the same inclusion and exclusion criteria applied as used for the discovery cohort. Eligibility was restricted to newly recruited patients, excluding individuals from the retrospective discovery cohort. The collected data were categorised into three severity grades: Grade 0 (no granulation, 0 points), Grade 1 (moderate granulation, 1–2 points), and Grade 2 (severe granulation, 3–6 points). Patients from the discovery cohort were then categorised accordingly.

### Sample preparation

To characterize the immunophenotype and structure of granulation tissue, a prospective cohort of EBV patients (Grade 0: *n* = 13–15, Grade 1: *n* = 13–14, Grade 2: *n* = 12) was enrolled using the scoring system (Fig. 1c) with comparable distribution between groups. In the years 2023 and 2024, biopsies from intact mucosa (Grade 0) or granulation tissue (Grades 1–2) were obtained by forceps biopsy at the Department of Interventional Pulmonology (University Hospital Essen – Ruhrlandklinik) [14]. Additional biopsies from stent patients (*n* = 6–9) were analysed separately due to difference in implantation techniques and heterogeneity of the underlying lung disease.

Tissue preparation was adapted from Schuler et al. [15]. The tissue was transferred into 5 mL digestion medium [DMEM/F-12 (11320033, Gibco, Waltham, Massachusetts, USA), DNase I (0.1 mg/ml, 11284932001, Merck, Darmstadt, Germany), collagenase (0.33 mg/ml, C9407-100 mg, Sigma Aldrich, St. Louis, Missouri, USA), hyaluronidase type IV (100 µg/ml, H4272-30MG, Sigma Aldrich). Depending on the tissue size, it was incubated at 37 °C for 20–30 min. If a larger piece of granulation tissue was obtained, the tissue was minced with a scalpel and pipetted up and down 10 times with a cut-off p1000 pipette tip. After the incubation, the digestion was stopped with 5 mL 1x DPBS (14190136, Invitrogen, Waltham, Massachusetts, USA) and the tissue was resuspended using a 10 mL pipette to further homogenize the pieces. The suspension was then centrifuged at 400 g for 8 min. The cell pellet was dissolved in 250 µl trypsin (Cat27250, Gibco)/EDTA (ED2SS-100G, Sigma Aldrich) and incubated for 5 min at room temperature. The reaction was stopped with 2 mL DMEM (P04-03600, Pan Biotech, Sydney, New South Wales) with 10% FBS (P30-3306, Pan Biotech), 1% penicillin-streptomycin (P-06–07100, Pan Biotech) and 2% L-glutamin (P04-80050, Pan Biotech). Using a cut-off p1000 pipette tip, the tissue was pipetted up and down 10 times. The cell suspension was then passed through a 70 μm cell strainer (83.3945.070, Sarsteadt, Numbrecht, Germany), which was rinsed with 4 mL 1x DPBS, and centrifuged at 400 g for 8 min. If many erythrocytes were present, 1 mL LCK lysis buffer (A10492-01, Gibco) was added and incubated at room temperature for 5–10 min. To stop the reaction, 9 mL 1x DPBS was added followed by a centrifugation step at 400 g for 8 min. The cell pellet was respuspended in 1 mL 1x DPBS and the cell numbers were counted.

### Flow cytometry

The staining of cells obtained from biopsies for flow cytometry was performed as previously described [16]. Dead cells were excluded using a viability dye (Zombie UV™, Biolegend, San Diego, United States). Surface staining of cells was performed using immune, epithelial, endothelial, and fibroblast markers (supplementary Tables 1–6) for flow cytometry and measured on a CytoflexLX (Beckmann Coulter, Brea, United States). Because biopsy samples were very small and not uniform in size, no weight-based normalization was feasible. For normalisation, immune cell percentages were calculated relative to CD45^+^ subsets and CD45^−^ subsets for tissue associated markers. Gating thresholds were defined by fluorescence-minus-one (FMO) controls. FMOs were generated using peripheral blood mononuclear cells (PBMCs) for immune-cell panels (CD45^+^ populations, Supplementary Tables 1–4) and tissue-derived positive cells for tissue associated panels (CD45^−^ populations, Supplementary Tables 5–6). Unstained samples were employed to assess background fluorescence and verify gating boundaries. Compensation matrices were monitored across all runs and gating consistency was validated through visual inspection of marker distributions. Data analyses was performed by FlowJo (Version 10.8.1, BD) with the gaiting strategies shown in the supplementary Figs. 2–7.

### Immunofluorescence staining and periodic acid-Schiff reaction

Biopsy sections (Grade 0: *n* = 3, Grade 1: *n* = 3, Grade 2: *n* = 3, stent: *n* = 3) were fixed in 4% paraformaldehyde (PFA) and stored at 4 °C. As previously described for histologic and immunofluorescence staining [17] samples were dehydrated by an ascending ethanol series and xylene as intermedium (HistoCore PEARL, Leica Biosystem, Wetzlar, Germany), and embedded in paraffin (TES 99, Medite, Burgdorf, Germany). Using a microtome (Microm HM 340E, Thermo Scientific, Waltham, USA), 3 μm-thick sections were cut. Afterwards, deparaffinised sections were stained by periodic acid Schiff’s (PAS) reaction to analyse the granulation tissue structure using a bright field light microscope. Briefly, deparaffinised samples were first hydrolysed in 1% w/v periodic acid solution before staining with Schiff’s reagent (both Merck). After a washing step in 35 °C warm tap water, cell nuclei were counterstained with Mayer’s hematoxylin (MHS16-500 mL, Sigma-Aldrich).

For immunohistological staining, the tissue was deparaffinised in a descending ethanol series. The samples were unmasked with 10 mM citrate buffer [citric acid (71289, Sigma-Aldrich) and sodium citrate (HN12.1, Carl Roth)]. The samples were then incubated with blocking solution [1xPBS, 0.1% tritron, 5% goat serum (X090710-8, Agilent Technologies, Santa Clara, California, USA)] for 60 min at room temperature. This was followed by an overnight incubation at 4 °C with the primary antibody 50 µL of the antibody solution (diluted in 100 mL PBS, 1 g BSA, 100 mg Na-azide). Briefly, the primary antibodies (supplementary Table 7) were incubated at 4 °C overnight. After three washing steps, and dilution are shown in supplementary Table 7. The sections were then washed three times for 5 min with wash buffer [Triton-X100 (0,1% v/v, Sigma Aldrich) in 1x DPBS the secondary antibody was added and incubated for 60 min at 37 °C. These included Alexa 488 goat-anti-rabbit (A11008, Invitrogen, 1:400) and Alexa Plus 555 goat-anti-mouse (A32727, Invitrogen, 1:400). After removal of the antibody solution, the membranes were washed three times and cell nuclei were counterstained with Hoechst 3342 Solution 15 (0.5 µg/mL, Chemometec, Lillerød, Denmark) before the membranes were covered with fluorescence mounting media (S302380-2, Agilent Technologies, Santa Clara, California, USA) and cover slips (Engelbrecht GmbH, Edermünde, Germany). Images were taken with an Echo Revolve (BICO, San Diego, United States) microscope and cluster images of the immunofluorescence staining were taken with an Axio Observer Z1 (Zeiss, Oberkochen, Germany) microscope.

### Statistical analysis

Statistical analyses were performed using GraphPad Prism (v9.4.1) and IBM SPSS Statistics (v29.0.0.0). Comparisons between groups were performed using Kruskal–Wallis tests, followed by Dunn’s multiple comparisons test. Correlations were evaluated using Spearman’s rank correlation coefficient. A *P*-value < 0.05 was considered statistically significant. To assess predictive validity of the visual scoring, we constructed receiver operating characteristic (ROC) curves using the endoscopic optical scale (0–4) as predictor and clinically relevant revision (yes/no) as binary outcome. Area under curve (AUC) values with 95% confidence intervals (95% CI) were calculated with bootstrap resampling (1,000 iterations). Inter-rater reliability for the granulation score was evaluated applying weighted Cohen’s kappa, which accounts for the ordinal nature of the scale. Two independent evaluators scored bronchoscopic recordings of both cohorts (*n* = 147). Kappa values were interpreted according to the Landis and Koch classification. Results are expressed as mean ± standard deviation, and significant *P*-values are reported in the Figure legends.

## Results

### Granulation leads to valve dysfunction

In the discovery cohort, an optical scaling and a scaling sheet were developed to classify granulation tissue as none, minimal, moderate, strong, or severe granulation (Figs. 1a + 2a + 2c, *n* = 56). In the discovery cohort, 31 patients (55.4%) showed no granulation tissue, while the remaining cases presented varying severities highlighting the heterogeneity of tissue response following valve implantation (Fig. 2c). An independent replication cohort (*n* = 91) with similar clinical characteristics was then recruited to develop a scoring combing the optical evaluation as well as the assessment in relation to factors such as interventional demand due to valve dysfunction and the need for tissue removal.

For subsequent scoring analyses, the discovery and replication cohorts (*n* = 147) were combined assessing the optical scaling and incorporating risk factors affecting valve function. To assess the reliability of the endoscopic granulation score, weighted Cohen’s kappa was calculated between two independent evaluators. Agreement was substantial, with a weighted κ of 0.75 (standard error of the mean = 0.04, 95% CI = 0.68–0.83, *p* < 0.001). These results indicate a high level of scoring consistency and support the robustness of the grading system for clinical use. Patients with COPD and EBV were on average 65 years old and had severe airflow obstruction as reflected by Gold stage III or IV. The forced expiratory volume in one second (FEV1) was 1.26 ± 0.94 L and the average 6-minute walk distance was 283 m. Additional patient characteristics are reported in supplementary Table 8. Based on the observed clustering of the five severity groups, three aggregated grades (Grade 0, Grade 1, and Grade 2) were defined to facilitate comparative and correlative analyses.

These data showed that 26.5% had no granulation tissue (Grade 0), 34.7% had moderate (Grade 1), and 38.8% had severe granulation (Grade 2, Fig. 2d). The severity score was significantly associated with valve dysfunction (*r* = 0.37, 95% CI: 0.22–0.51, *P* < 0.001), dislocation (*r* = 0.33, 95% CI: 0.18–0.47, *P* < 0.001), and revision (*r* = 0.40, 95% CI 0.25–0.53, *P* < 0.001; supplementary Table 9). No relationship between the degree of granulation tissue and blood, lung, or clinical markers was found (supplementary Table 8 + 9). Notably, over 70% of patients developed hypergranulation, which was linked to poorer treatment success.

### Predictive granulation score using optical scaling

The granulation tissue score showed good discriminatory power for identifying patients who developed valve dysfunction (AUC = 0.79, 95% Cl = 0.70–0.88, *p* < 0.001). It showed equally strong accuracy for the risk of valve dislocation (AUC = 0.77, 95% Cl = 0.68–0.86, *p* < 0.001). The ability to predict clinically relevant revision bronchoscopy (e.g. for obstruction or dysfunction) was similarly robust (AUC = 0.79, 95% Cl = 0.71–0.88, *p* < 0.001, Table 1).

**Table 1:** ROC analysis of optical scale for three clinically relevant outcomes

Outcome	AUC	95% Cl	*p*-value
Dysfunction	0.79	0.70–0.88	< 0.001
Dislocation	0.77	0.68–0.86	< 0.001
Revision	0.79	0.71–0.88	< 0.001

Receiver operating characteristic (ROC), area under the curve (AUC), 95% confidence interval (95% CI)

### Granulation degree affects immunophenotypes

A prospective cohort of COPD patients with EBV implants (*n* = 41, GOLD stage III or IV) or stent patients (*n* = 9) was recruited and biopsies were obtained. No significant differences in demographics, FEV1, or 6-minute walk distance compared to the discovery and replication cohorts were observed (supplementary Tables 10–13).

To assess cellular and structural changes relative to granulation severity, biopsies (Grade 0: *n* = 3, Grade 1: *n* = 3, Grade 2: *n* = 3, stent: *n* = 3) were analysed by PAS (supplementary Fig. 11). Histological analyses revealed that increasing granulation severity was associated with progressive epithelial and extracellular matrix disorganisation and a marked rise in immune cell infiltration. Stent-associated tissue showed comparable alterations to severe EBV-related granulation.

**Fig. 3:**
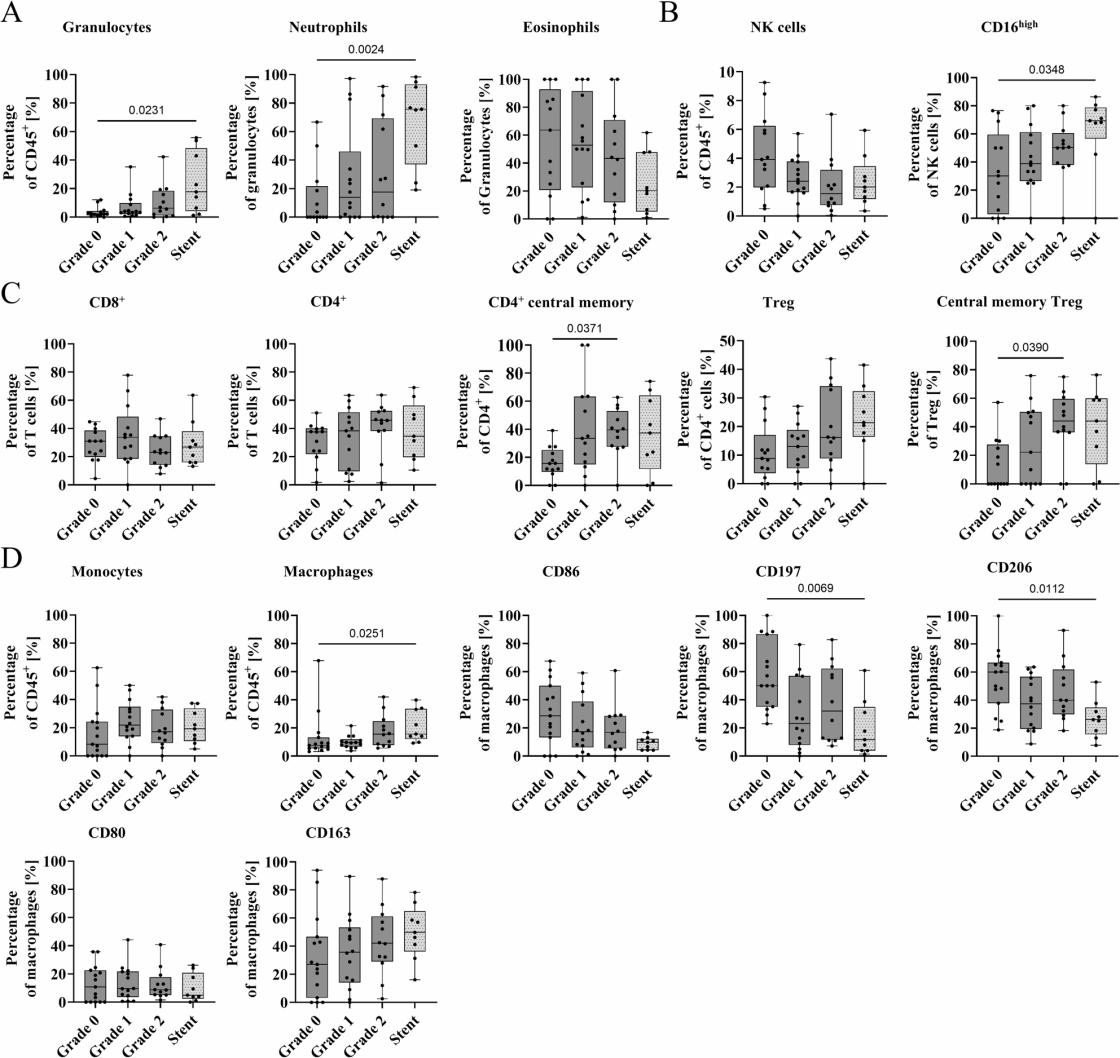
Immunophenotyping of inflammatory cells in biopsies from valve (Grade 0, Grade 1, Grade 2) and stent patients. Percentage of **A** granulocytes in CD45^+^, neutrophils from granulocytes, and eosinophils from granulocytes, **B** natural killer (NK) cells (CD56^+^) in CD45^+^ and NK cells positive for CD16^high^, **C** CD4^+^ T helper cells from CD3^+^, cytotoxic CD8^+^ T cells from CD3^+^, CD4^+^ central memory cells (CD45RA^−^/CD62L^+^) from CD4^+^, regulatory T cells (Treg; CD25^+^/CD127^low^) from CD4^+^, and central memory cells (CD45RA^−^/CD62L^+^) in regulatory T cells **D** monocytes (CD14^+^/CD16^+^) in CD45^+^, macrophages in CD45^+^ and their activation markers CD86, CD197, CD206, CD80, and CD163. Results are expressed as mean ± standard deviation (plot: min to max, all points). Statistical analyses were performed by Kruskal-Wallis test followed by Dunn’s multiple comparisons test. Grade 0: *n* = 13–15, Grade 1: *n* = 13–14, Grade 2: *n* = 12, stent: *n* = 9

**Fig. 4:**
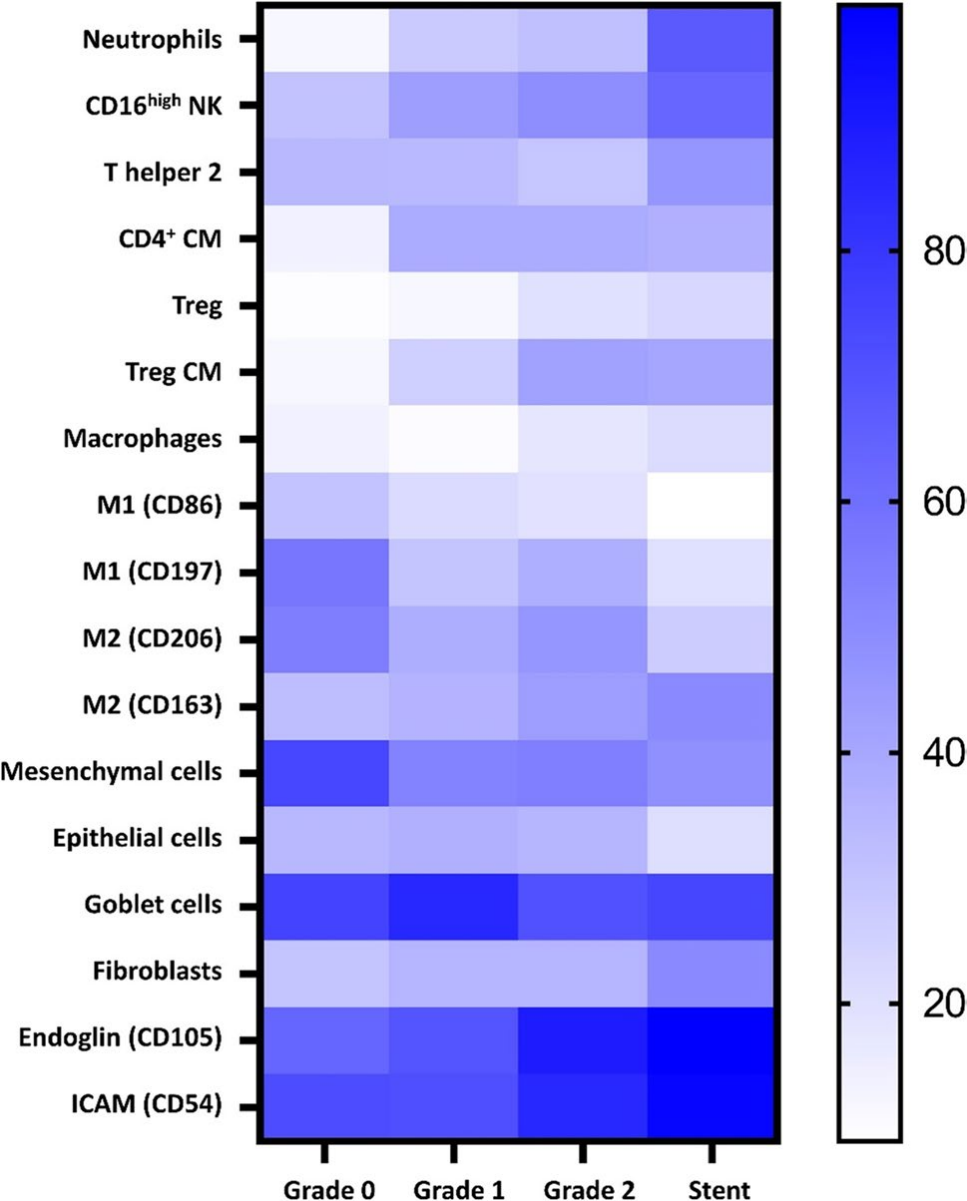
Heat map summarising key findings of immune and structural cell populations in tissue samples from patients with different grades of granulation tissue and from stent patients. Immune subsets include neutrophils, CD16^high^ NK cells, type 2 T helper (Th2) cells, CD4^+^ central memory cells (CM), regulatory T cells (Treg) and Treg central memory cells, as well as macrophages (total and polarised M1 and M2 macrophages). Structural components comprise mesenchymal cells, epithelial cells, goblet cells, fibroblasts, as well as markers of angiogenesis (endoglin) and cell adhesion (ICAM). Colour intensity represents the mean proportion (%) of each cell population within the respective group, with darker blue indicating higher frequency of the respective cell population. Columns represent mean values from Grade 0, Grade 1, Grade 2 and stent samples

As an influx of immune cells were observed in biopsies of patients with more severe granulation tissue formation, a refined analysis of the cellular composition was carried out. Detailed gating strategies and an overview of used antibodies are provided in supplementary material (supplementary Figs. 2–7, supplementary Tables 1–6). With increasing granulation severity, more granulocytes and neutrophils were detected, which was most pronounced in stent patients (Fig. 3a). While the percentage of eosinophils decreased (Fig. 3a), natural killer (NK) cells (Fig. 3b) were present in all groups, with heightened CD16^high^ expression in stent patients (Fig. 3b). T lymphocyte subsets also contributed to the immune cell influx in the granulation tissue. EBV patients with severe granulation had more CD4^+^ central memory cells and regulatory T cells (Tregs) with a memory phenotype (Fig. 3c). While minimal immune cell infiltration in Grade 0 mucosal biopsies was revealed, Grade 1 and 2 patients were marked by a gradual increase in CD8^+^ T cells, CD39^+^ activation markers, Th17 and Th1/Th17 cells, and tissue-resident memory T cells (CD69^+^CD103^+^). Stent-derived samples showed a similar immunological profile to Grade 2, including elevated alternative monocytes and plasmacytoid dendritic cells (supplementary Figs. 8 + 9). In Grade 1 granulation tissue, there was a predominance of classically activated M1-like macrophages (M1; CD80^+^, CD86^+^, CD64^+^). In contrast, Grade 2 and stent-associated tissue showed a decrease in both M1 markers (CD86, CD197, CD80) and the alternatively activated macrophage (M2) marker CD206, along with a trend towards higher CD163 expression (M2, Fig. 3d). These data showed that, overall, the immunophenotype of Grade 2 EBV patients more closely resembled that of stent patients than to patients with lower grades. However, the heatmap of the key cellular markers revealed some distinct immune signatures separating the different severity grades and stent patients (Fig. 4).

### Endoglin upregulation in severe granulation

**Fig. 5:**
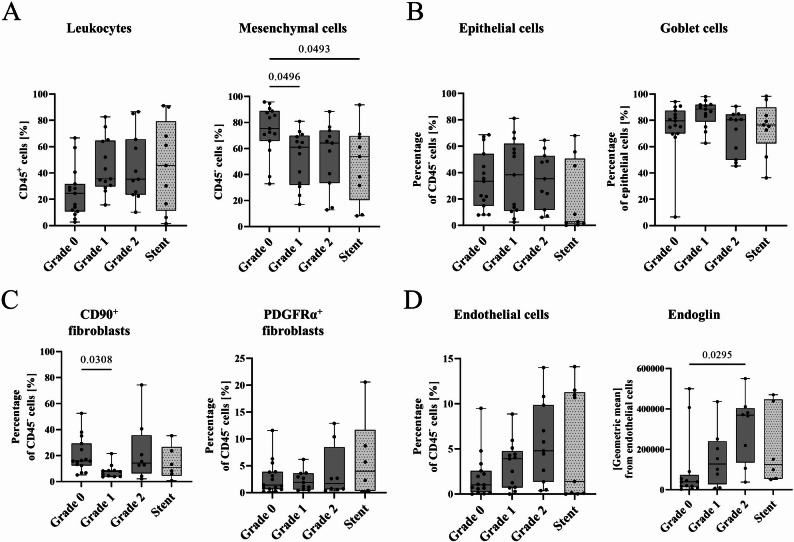
Tissue composition of biopsies from EBV patients (Grade 0, Grade 1, Grade 2) or stents analysed by flow cytometry. Percentage of **A** leukocytes (CD45^+^) and mesenchymal cells (CD45^−^) cells, **B** epithelial cells (CD326^+^) and goblet cells (TSPAN8^+^) **C** fibroblasts (CD90^+^ or PDGFRα^+^), **D** endothelial cells (CD31^+^) and the mean fluorescence intensity [geometric mean] of endoglin on endothelial cells. Results are expressed as mean ± standard deviation (plot: min to max, all points). Statistical analyses were performed by Kruskal-Wallis test followed by Dunn’s multiple comparisons test. Grade 0: *n* = 13–15, Grade 1: *n* = 13–14, Grade 2: *n* = 12, stent: *n* = 6–9

In contrast to the immune cell influx, there was a significant reduction in CD45^−^ mesenchymal cells in EBV (Grade 1) and stent patients compared to intact mucosa, with a similar trend in patients with severe granulation (Fig. 5a). Over 80% of epithelial cells were goblet cells (Fig. 5b) with a similar fibroblast distribution across all groups (Fig. 5c). Endothelial cell percentage tended to rise with increasing granulation severity, supported by differential endoglin expression (Fig. 5c). Additionally, Grade 1 and 2 granulation tissue showed a trend towards increased expression of endothelial activation markers (VCAM-1, VEGFR-2, supplementary Fig. 10). Percentage of fibroblasts (FSP1^+^, α-SMA^+^) were low in Grade 0, increased in Grade 1, and reached the highest level in tissue from Grade 2 EBV and stent patients (supplementary Fig. 10). Notably, while CD45^−^ mesenchymal cells in granulation tissue decreased, there was a marked rise in endoglin-expressing endothelial cells in those with severe granulation (Fig. 4).

### Granulation impact on tissue integrity

**Fig. 6:**
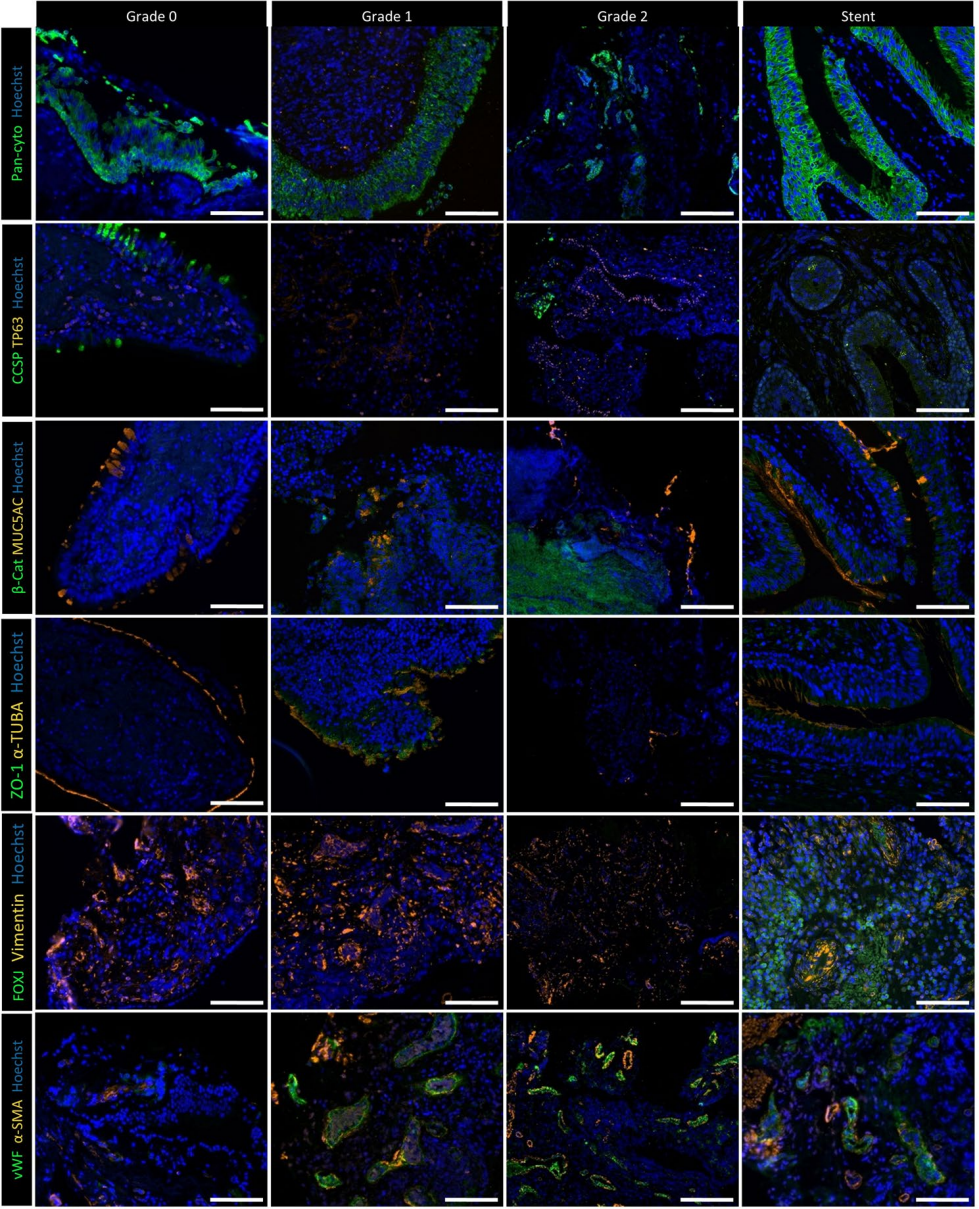
Immunofluorescence staining of epithelial cell and structural markers in tissue samples from patients with different grades of granulation tissue and from stent patients. A total of three patients per group (Grade 0, Grade 1, Grade 2, stents) were analysed (supplementary Figure 12-23). Representative images of one patient per group are shown. Pan-cytokeratin (green) for epithelial cells, CCSP (green) for club cells, and TP63 (orange) for basal cells. β-Catenin (green) for cellular adhesion and proliferation, and MUC5AC (orange) for goblet cells. ZO-1 (green) for tight junctions and α-Tubulin (orange) for ciliated cells. FOXJ (green) is a transcription factor for ciliogenesis and vimentin (orange) for mesenchymal cells. VWF (green) for endothelial cells and α-SMA for myofibroblasts or smooth muscle cells. Scale bar: 100 µm; images acquired at 20× magnification

**Fig. 7:**
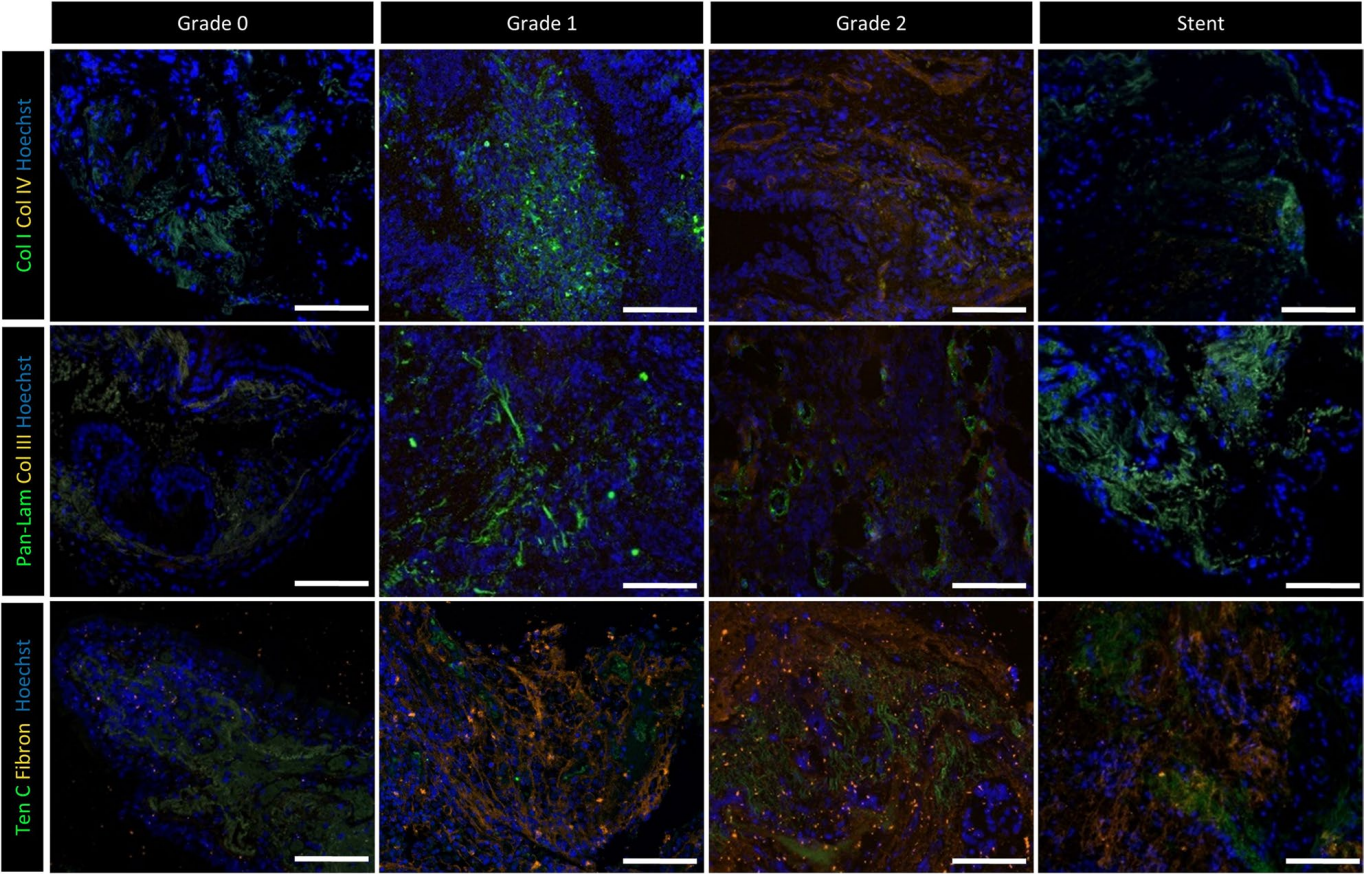
Immunofluorescence staining of extracellular matrix remodelling markers across granulation tissue with different severity grades and stent biopsies. A total of three patients per group (Grade 0, Grade 1, Grade 2, stents) were analysed (supplementary Fig. 12–23). Representative images of one patient per group are shown. Collagen I (green) extracellular matrix in wound healing and collagen IV (orange) for basal membrane during wound healing. Pan-laminin (green) for basal membrane during wound healing and collagen III (orange) for tissue regeneration and stiffness. Tenascin C (green) extracellular matrix in early wound healing or fibrosis and fibronectin (orange) for acute wound healing or fibrosis. In all images, cell nuclei (Hoechst 3342) are depicted in blue. Scale bar: 100 μm; images acquired at 20× magnification

The localisation and ECM integrity was qualitatively assessed by immunofluorescence staining on biopsies including tissue from patients with intact mucosa (Grade 0, *n* = 3), moderate (Grade 1, *n* = 3), severe (Grade 2, *n* = 3), and stent granulation tissue (*n* = 3). Although no quantitative image analysis was performed in this study, localisation and signal intensity were consistent across replicates (representative overview: Figures 6 and 7, each individual patient: supplementary Figs. 12–23) Epithelial cells (pan-cytokeratin) were present in all groups, with weaker signals in some Grade 2 and stent patients (Fig. 6), supplementary Figs. 12–23). Club (CCSP), basal (TP63), goblet (MUC5AC), and ciliated cells (ac. TUBA) were found regardless of the granulation severity with similar findings for FOXJ, a ciliogenesis transcription factor. Overall, the epithelial structure was better in patients without granulation than in any other group. In contrast, the vimentin signal, representing mesenchymal cells, was stronger in Grade 1, Grade 2, and stent patients (Fig. 6, supplementary Figs. 12–23). Mesenchymal cells were mainly localised in the connective tissue, including endothelial cells (vWF), myofibroblasts/smooth muscle cells (α-SMA), and fibroblasts (FSP1, Fig. 6, supplementary Figs. 12–23). ECM markers for cell adhesion (β-catenin) and fibrosis (perlecan) were strongly expressed in most granulation biopsies. While the tight junction (ZO-1) signal was lower in granulation tissue compared to healthy mucosa, collagen I levels seemed elevated only in intact mucosa and stent patients (Fig. 6, supplementary Figs. 12–23). The basal membrane (pan-laminin) was disorganised in all analysed granulation tissue, but tenascin C and fibronectin expression were elevated in Grade 2 and stent patients (Fig. 7, supplementary Figs. 12–23). Taken together, epithelial and mesenchymal cells were detectable, but impaired epithelial integrity and altered ECM markers existed in patients with a high granulation score or stents.

## Discussion

### Clinical relevance of granulation tissue in airway implants

Granulation tissue formation is a major complication in interventional pulmonology and limits the long-term success of EBV and airway stents. Understanding the underlying pathomechanisms of granulation tissue formation is critical for improving patient care. In this study, we applied a newly developed bronchoscopic scoring system for classifying hypergranulation after EBV implantation in large independent cohorts. Patients with severe granulation exhibited loss of ECM integrity and increased immune cell infiltration. A detailed immunophenotyping of granulation tissue revealed elevated levels of granulocytes, neutrophils, NK cells, and Tregs. These patients showed a shift towards M2 macrophages and increased mesenchymal cell numbers, including elevated endoglin expression in endothelial cells.

### Validation and utility of the novel scoring system

Granulation tissue formation can affect patients with various diseases. Tracheal stenosis, often due to prolonged mechanical ventilation, was seen in a third of patients, with 96% developing severe hypergranulation [18]. Similarly, COPD patients with EBV showed granulation tissue formation, which impacted valve function [19]. Although stents are used for central airway obstruction in conditions like lung cancer, lung transplantation, tracheobronchomalacia, fistulas, or pneumonectomy stumps, granulation tissue can reduce treatment success and patients’ quality of life [20]. Using our newly developed score, we were able to more accurately assess the prevalence and severity of granulation tissue identifying over 70% of EBV patients with hyperplastic granulation tissue. Based on very limited literature reports involving cohorts undergoing revision bronchoscopy, a prevalence of 40–50% was assumed [8, 9]. This discrepancy can be attributed to the fact that previous studies [8, 9] only included patients whose granulation tissue necessitated valve revision. In contrast, our cohort also encompassed COPD patients without or only mild granulation tissue that did not interfere with EBV therapy. The scoring system included additional factors such as the target lobe, dislocation, dysfunction, or revision of the EBVs, which enabled a more accurate characterisation of the degree of granulation tissue. While the scoring system incorporates factors such as valve dysfunction and the need for granulation tissue removal, these components were used solely for classification purposes and not for predictive analyses. To avoid circular reasoning, statistical associations between score severity and clinical outcomes were calculated independently from the scoring assignment process. We found a positive correlation between the severity of granulation and poorer EBV function, which highlights the negative impact of granulation tissue on treatment success. The strong correlation between granulation severity and valve-related complications underscores the potential of our grading system as a valuable clinical tool for monitoring implant patients at each follow-up visit. The robust predictive analyses further supported the clinical relevance of the newly developed optical scaling system demonstrating a significant discriminatory ability across valve dislocation, dysfunction, and revision bronchoscopy.

### Distinct immune profiles in severe granulation tissue

Immunophenotyping revealed progressive lymphocyte infiltration and ECM alterations with increasing granulation severity. Immune cell recruitment, as a foreign body response by release of pro-inflammatory factors has been described in coronary valves affected by shear stress [21]. In the lung, friction and pressure during breathing may influence the foreign body response, though the role of shear stress in promoting granulation tissue and immune cell recruitment needs further clarification. The absence of immune activation in implant patients without granulation tissue formation supports its role as a non-pathological baseline. In contrast, the progressive accumulation of immune cells from Grade 1 to Grade 2 EBV patients, as well as granulation tissue from stent patients, indicates a dynamic immune response driven by mechanical irritation and foreign body exposure. Predominant granulocytes like neutrophils and NK cells, as well as higher percentages of Tregs, were observed, particularly in stent patients. Tregs can interfere with cytotoxic CD8^+^ T cells in a TGF-β-dependent manner and promote a fibrotic response [22, 23]. The majority of Tregs in patients with hypergranulation were of a central memory phenotype (CD4^+^CD62L^+^), which has been implicated in another respiratory disorder including granulomatosis patients with polyangiitis (Wegener’s disease) [24]. As central memory T cells can convert into Tregs in response to TGF-β [25], this may suggest that the accumulation of CD62L^+^ Tregs may reflect a TGF-β-associated environment. In future studies, quantification of TGF-β or downstream signalling events are required to further validate this proposed mechanism. Although present in low numbers, NK cells exhibited higher CD16 expression in stent patients. In general, NK cells can have several functions during wound healing, including re-epithelialisation, angiogenesis, and tissue remodelling [26]. The altered microenvironment due to the foreign body may release specific signals that lead to the upregulation of CD16, a key receptor for antibody-dependent cellular cytotoxicity [27]. In turn, an enhanced cytotoxic potential and antibody-dependent responses of the remaining NK cells may be induced.

### M2 macrophage polarisation and fibrotic microenvironment

Consistent with chronic tissue stress and repair mechanisms, the phenotype switch of macrophages suggests a temporal transition from acute inflammation to tissue remodelling and an ECM-modifying phenotype, particularly in Grade 2 and stent patients. Macrophages shifted towards a CD163^high^ M2 phenotype in high-grade granulation tissue, indicating a microenvironment associated with tissue remodelling [28, 29]. This was accompanied by a loss of classical M1 and M2 surface markers (CD86, CD206). M1 promote implant degradation, while M2 secrete IL-10 and TGF-β, influencing ECM remodelling via matrix metalloproteinase (MMPs) [30]. Phenotypic plasticity plays a pivotal role in inflammation and tissue remodelling [31], which is associated with M2 marked by CD206 and CD163 [32–34]. In fibrotic disease, CD206 is often downregulated at certain stages and subtypes such as CD163^+^ macrophages become more dominant [35, 36]. CD163^+^ macrophages are often associated with anti-inflammatory and immunosuppressive functions [37, 38]. Emerging evidence indicates context dependent pro-fibrotic roles and their production of TGF-β [33, 39, 40]. However, expression of CD163 does not exclusively indicate a fibrotic-anti-inflammatory phenotype. The functional profile of macrophages is highly context-dependent and depending on the microenvironment, encompass both pro-and anti-inflammatory programs [41, 42]. Consistent with the observed ECM remodelling, this points to a shift towards a macrophage phenotype potentially linked to tissue remodelling and fibrosis. This reinforces the concept that chronic implant-driven inflammation and excessive tissue reactivity may promote fibrotic-like alterations in severe granulation tissue over time. To our knowledge, this is the first characterisation of the immune cell composition across granulation tissue severity grades as well as in stent patients. The shared immune cell and fibrotic program may therefore be driven by local immunoregulation rather than systemic inflammation proposing a novel pathomechanism.

### Extracellular matrix remodelling and epithelial disruption

Our data demonstrate that fibroblast expansion and extracellular matrix deposition are hallmark features of advanced granulation and stent-associated tissue, which is absent in physiological mucosa (Grade 0). Thus, structural remodelling maybe tightly linked to immune cell activity and hypergranulation. Mesenchymal cells such as epithelial cells, fibroblasts, and endothelial cells played a significant role in granulation tissue. Epithelial cells consisted largely of goblet cells, which was comparable in all groups, including the intact mucosa of EBV patients without hypergranulation. This was not surprising as goblet cell hyperplasia is a known feature of COPD [43]. Endothelial cells in patients with increasing granulation showed elevated endoglin expression, a marker essential for angiogenesis and immune cell recruitment to activated endothelial cells [44]. This may help explain the heightened immune cell infiltration. The increased presence of goblet and club cells, along with endothelial activation, likely represents a mucosal adaption to persistent irritation and local hypoxia, further supporting a model of chronic wound healing in Grade 2 and stent-associated tissue.

Overall, the structure of granulation tissue varied widely across patient groups. Fully differentiated epithelial cells were present in all groups, with ciliated cells being less prominent in EBV patients with severe granulation and stent patients. Mesenchymal cells, including fibroblasts, were more abundant in these patients. Granulation tissue also showed strong β-catenin expression, a key mediator in fibrotic diseases [45, 46]. In addition, granulation tissue was positive for fibronectin and tenascin C, both promoting fibroblast migration and angiogenesis [47]. Fibronectin is involved in the fusion of macrophages to form foreign body giant cells [48, 49]. This further supports an impaired epithelial integrity and a disrupted ECM compared to intact mucosa. The results must be replicated in the future in a larger cohort.

### Differences between EBV and stent-associated granulation tissue

The differences between EBV and stent patients is likely attributable to an inherently increased heterogeneity of underlying disease, stent material, designs and anatomical locations, as well as distinct mechanical forces. Finally, the local tissue environment, including pre-existing inflammation, vascularization, and extracellular composition, may differ between anatomical sites, further modulation immune cell recruitment and activation [50, 51]. While our study cannot definitively disentangle these factors, our findings suggest that the interplay between device, the individual bacterial colonisation and tissue context contributes to the specific immunological and structural profiles observed in stent patients.

### Clinical implications and therapeutic perspectives

Most of our patients where biopsies were obtained, were diagnosed with COPD and were treated with inhaled corticosteroids (ICS). Despite the efficacy of ICS in patients with eosinophilic inflammation, the immune response in granulation tissue was predominantly neutrophilic and macrophage-driven, hallmarks of steroid-resistant inflammation. Thus, this treatment approach may be less effective in these patients, which highlights the need for novel therapeutics targeting non-eosinophilic pathways in patients with hypergranulation. Macrolides and biologics may also be of interest as potential therapies for patients developing granulation, as both are known to modulate immune cell populations involved in granulation tissue formation [52, 53].

Our data suggest that targeting macrophage polarisation, TGF-β signaling, or matrix remodelling may offer new therapeutic options for implant-associated airway remodelling. This closely aligns with the work of Xue et al., which explored the TGF-β/Smad signaling pathway and fibroblast proliferation of granulation tissue from stent patients [54]. Furthermore, the histological and immunological parallels between Grade 2 EBV and stent patients highlight shared pathomechanisms despite distinct clinical indications. The shared mechanisms of TGF-β-driven immune cell recruitment and ECM alterations suggest that targeted modulation of the TGF-β/Smad signaling pathway, potentially through immunomodulatory therapies, could be an effective strategy for controlling granulation tissue development and its associated complications. By stabilizing the immune-modulated microenvironment and regulating ECM remodelling, such therapies may offer a promising strategy to mitigate granulation-associated tissue remodelling with potential progress towards fibrosis-like changes and improve treatment outcomes.

### Limitations and future directions

A limitation of our study is the need for enzymatic digestion to isolate lung cells, which may affect their viability and expression of surface markers potentially skewing cell representation. This was overcome by combining flow cytometry on isolated cells with analysing native biopsy tissue by PAS and immunofluorescence staining. Another shortcoming of our study is the heterogeneity in time intervals between valve or stent implantation and biopsy acquisition. Due to the retrospective nature of the scoring and clinical need for bronchoscopy, sample collection varied between patients, which may have impacted immune signatures as the local inflammatory and remodelling processes can evolve over time. Our optical scaling system was developed based on predefined morphological criteria, similar to current clinical practice in interventional pulmonology, that were validated through inter-observer agreement and supported by representative reference images. Combing this approach with additional risk factors impacting valve function has led to a scoring that is unique in this field, and may pave the way for future automated image analyses or artificial intelligence (AI)-based scoring tools. Future longitudinal studies are now needed to explore this avenue to validate the score as a predictive tool for risk stratification as well as temporal dynamics of granulation formation in more detail and. Mechanistic in vitro studies could further dissect the complex interplay between immune cells, mechanical forces, and the ECM in driving this unique fibro-inflammatory response.

## Conclusion

In summary, high-grade granulation tissue in response to implants represent a persistent, maladaptive tissue response. This study provides a comprehensive classification of implant-associated granulation tissue, highlighting increased immune and mesenchymal cells specifically in patients with Grade 2 EBV or stents. Analyses of ECM composition and tissue structure revealed how granulation tissue may impact EBV dysfunction and the need for revision bronchoscopy, with potential implication for patient outcomes. The scoring system developed here supports clinical decision-making by identifying patients at higher risk for severe tissue overgrowth, informing the need for regularly follow-up visits, and guiding consideration of targeted therapies. Collectively, these insights may help guide the development of novel targeted therapies addressing macrophage- and neutrophil-driven inflammation, which is typically corticosteroid-resistant in COPD patients.

## Supplementary Information

Supplementary Material 1.

## Data Availability

The datasets generated and analysed are available from the corresponding author on request.

## Abbreviations

α-SMA: Alpha smooth muscle actin
α-Tubulin: Alpha tubulin
β-Catenin: Beta-catenin
µL: Micro litre
6MWT: 6-minute walk test
95% CI: 95% confidence intervals
AI: Artificial intelligence
AUC: Area under curve
BMI: Body mass index
CAT: COPD assessment test
CCSP: Clara cell secretory protein
CD: Cluster of differentiation
COPD: Chronic obstructive pulmonary disease
CRP: C-reactive protein
DAMPs: Damage-associated molecular patterns
DC: Dendritic cell
DLCO: Diffusing capacity of the lungs for carbon monoxide
EBVs: Endobronchial valves
ECM: Extracellular matrix
FEV1: Forced expiratory volume in one second
FEV1/FVC: Tiffeneau-index
FMO: Fluorescence-minus-one controls
FOXJ: Forkhead box protein J
FSP1: Fibroblast specific protein 1
FVC: Forced expiratory vital capacity
HLADR: Human leukocyte antigen – DR isotype
ICAM-1: Intercellular adhesion molecule 1
KCO: Carbon monoxide transfer coefficient
M1: Classically activated/pro-inflammatory macrophages
M2: Alternative activated/anti-inflammatory macrophages
MCAM: Melanoma cell adhesion molecule
MMP: Matrix metalloproteinase
MUC5AC: Mucin 5AC
NK: Natural killer cells
NKT: Natural killer T cell
nL: Nano litre
PAS: Periodic acid-Schiff’s reaction
PBMCs: Peripheral blood mononuclear cells
pCO2: Partial pressure of carbon dioxide
PFA: Paraformaldehyde
pH: Potential hydrogen
pO2: Partial pressure of oxygen
ROC: Receiver operating characteristic
RV: Residual volume
TEMRA: Terminal differentiated T cells
Th: T helper cell
TLC: Total lung capacity
TP63: Tumor protein p63
Treg: Regulatory T cells
Vc Max: Maximal vital capacity
V-CAM-1: Vascular cell adhesion protein 1
VE-Cadherin: Vascular endothelial cadherin
VEGFR-2: Vascular endothelial growth factor receptor 2
VWF: Von Willebrand factor
Z0-1: Zonula occludens protein 1

## References

[1] Li L, Zhang X, Shi J, Wang Y, Chen H, Liu F, et al. Airway stents from now to the future: a narrative review. Respiration. 2023;102(6):439–48. 10.1159/000530421. 37232032 10.1159/000530421

[2] Koster TD, Klooster K, ten Hacken NH, et al. Endobronchial valve therapy for severe emphysema: an overview of valve-related complications and its management. Expert Rev Respir Med. 2020;14(12):1235–47. 10.1080/17476348.2020.1813571. 32842819 10.1080/17476348.2020.1813571

[3] Criner GJ, Sue R, Wright S, et al. A multicenter randomized controlled trial of zephyr endobronchial valve treatment in heterogeneous emphysema (LIBERATE). Am J Respir Crit Care Med. 2018;198(9):1151–64. 10.1164/rccm.201803-0590OC. 29787288 10.1164/rccm.201803-0590OC

[4] Sciurba FC, Ernst A, Herth FJF, et al. A randomized study of endobronchial valves for advanced emphysema. N Engl J Med. 2010;363(13):1233–44. 10.1056/NEJMoa0900928. 20860505 10.1056/NEJMoa0900928

[5] Herth FJF, Noppen M, Valipour A, et al. Efficacy predictors of lung volume reduction with zephyr valves in a European cohort. Eur Respir J. 2012;39(6):1334–42. 10.1183/09031936.00161611. 22282552 10.1183/09031936.00161611

[6] Hu HC, Liu YH, Wu YC, et al. Granulation tissue formation following dumon airway stenting: the influence of stent diameter. Thorac Cardiovasc Surg. 2011;59(3):163–8. 10.1055/s-0030-1250667. 21480137 10.1055/s-0030-1250667

[7] Damaraju V, Sehgal IS, Muthu V, et al. Bronchial valves for persistent air leak: a systematic review and meta-analysis. J Bronchol Interv Pulmonol. 2024;31(3). 10.1097/LBR.0000000000000964. 10.1097/LBR.0000000000000964 38716831

[8] Roodenburg SA, Klooster K, Hartman JE, et al. Revision bronchoscopy after endobronchial valve treatment for emphysema: indications, findings and outcomes. Int J Chron Obstruct Pulmon Dis. 2021;16:1127–36. 10.2147/COPD.S302662. 33911858 10.2147/COPD.S302662 PMC8071701

[9] Gompelmann D, Gerovasili V, Kontogianni K, et al. Endoscopic valve removal 180 days since implantation in patients with severe emphysema. Respiration. 2018;96(4):348–54. 10.1159/000489887. 30041242 10.1159/000489887

[10] Gupta A, Slebos DJ, Pouwels SD. Lung implantable devices: the issue with granulation tissue. Breathe (Sheff). 2025;21(3):240243. 10.1183/20734735.0243-2024. 40673062 10.1183/20734735.0243-2024 PMC12260914

[11] Roodenburg SA, Pouwels SD, Slebos DJ. Airway granulation response to lung-implantable medical devices: a concise overview. Eur Respir Rev. 2021;30(161). 10.1183/16000617.0066-2021. 10.1183/16000617.0066-2021 PMC9488845 34348981

[12] Ost DE, Shah AM, Lei X, et al. Respiratory infections increase the risk of granulation tissue formation following airway stenting in patients with malignant airway obstruction. Chest. 2012;141(6):1473–81. 10.1378/chest.11-2005. 22194585 10.1378/chest.11-2005 PMC4694180

[13] Saad CP, Murthy S, Krizmanich G, et al. Self-expandable metallic airway stents and flexible bronchoscopy: long-term outcomes analysis. Chest. 2003;124(5):1993–9. 10.1378/chest.124.5.1993. 14605078 10.1378/chest.124.5.1993

[14] Barbet K, Schmitz MS, Westhölter D, et al. Bronchoscopic biopsies - a novel source for primary airway epithelial cells in respiratory research. Respir Res. 2024;25(1):439. 10.1186/s12931-024-03060-1. 39719562 10.1186/s12931-024-03060-1 PMC11669235

[15] Schuler M, Cuppens K, Plönes T, et al. Neoadjuvant nivolumab with or without relatlimab in resectable non-small-cell lung cancer: a randomized phase 2 trial. Nat Med. 2024;30(6):1602–11. 10.1038/s41591-024-02965-0. 38689060 10.1038/s41591-024-02965-0 PMC11186754

[16] Raspe J, Schmitz MS, Barbet K, et al. Therapeutic properties of Helicobacter pylori-derived vacuolating cytotoxin A in an animal model of chronic allergic airway disease. Respir Res. 2023;24(1):178. 10.1186/s12931-023-02484-5. 37415170 10.1186/s12931-023-02484-5 PMC10324189

[17] Luengen AE, Kniebs C, Buhl EM, et al. Choosing the right differentiation medium to develop mucociliary phenotype of primary nasal epithelial cells in vitro. Sci Rep. 2020;10(1):6963. 10.1038/s41598-020-63922-8. 32332878 10.1038/s41598-020-63922-8 PMC7181704

[18] Ghiani A, Tsitouras K, Paderewska J, et al. Tracheal stenosis in prolonged mechanically ventilated patients: prevalence, risk factors, and bronchoscopic management. BMC Pulm Med. 2022;22(1):24. 10.1186/s12890-022-01821-6. 34991555 10.1186/s12890-022-01821-6 PMC8740413

[19] Patel M, Chowdhury J, Zhao H, et al. Meta-analysis and systematic review of bronchoscopic lung volume reduction through endobronchial valves in severe emphysema. J Bronchol Interv Pulmonol. 2022;29(3):224–37. 10.1097/LBR.0000000000000872. 10.1097/LBR.0000000000000872 PMC9233031 35698281

[20] Lee P, Kupeli E, Mehta AC. Airway stents. Clin Chest Med. 2010;31(1):141–50. 10.1016/j.ccm.2009.08.002. 20172440 10.1016/j.ccm.2009.08.002

[21] Hilborn J, Bjursten LM. A new and evolving paradigm for biocompatibility. J Tissue Eng Regen Med. 2007;1(2):110–9. 10.1002/term.4. 18038399 10.1002/term.4

[22] Mempel TR, Pittet MJ, Khazaie K, Weninger W, Weissleder R, von Boehmer H, et al. Regulatory T cells reversibly suppress cytotoxic T cell function independent of effector differentiation. Immunity. 2006;25(1):129–41. 10.1016/j.immuni.2006.04.015. 16860762 10.1016/j.immuni.2006.04.015

[23] Tennyson L, Rytel M, Palcsey S, et al. Characterization of the T-cell response to polypropylene mesh in women with complications. Am J Obstet Gynecol. 2019;220(2):e1871–8. 10.1016/j.ajog.2018.11.121. 10.1016/j.ajog.2018.11.121 PMC6557122 30419195

[24] Lamprecht P, Erdmann A, Mueller A, et al. Heterogeneity of CD4^+^ and CD8^+^ memory T cells in localized and generalized wegener’s granulomatosis. Arthritis Res Ther. 2003;5(1):R25–31. 10.1186/ar610. 12716450 10.1186/ar610 PMC154430

[25] Zhang X, Chang Li X, Xiao X, et al. CD4+/CD62L + central memory T cells can be converted to Foxp3^+^ T cells. PLoS ONE. 2013;8(10):e77322. 10.1371/journal.pone.0077322. 24155942 10.1371/journal.pone.0077322 PMC3796486

[26] Liippo J, Toriseva M, Kähäri VM. Natural killer cells in wound healing. In: Lotze MT, Thomson AW, editors. Natural killer cells: basic science and clinical application. San Diego: Academic; 2010. pp. 519–25. 10.1016/B978-0-12-370454-2.00039-9.

[27] Sablik KA, Litjens NHR, Klepper M, Betjes MGH. Increased CD16 expression on NK cells is indicative of antibody-dependent cell-mediated cytotoxicity in chronic-active antibody-mediated rejection. Transpl Immunol. 2019;54:52–8. 10.1016/j.trim.2019.02.005. 30794946 10.1016/j.trim.2019.02.005

[28] Mantovani A, Sica A, Sozzani S, et al. The chemokine system in diverse forms of macrophage activation and polarization. Trends Immunol. 2004;25(12):677–86. 10.1016/j.it.2004.09.015. 15530839 10.1016/j.it.2004.09.015

[29] Stahl M, Schupp J, Jäger B, et al. Lung collagens perpetuate pulmonary fibrosis via CD204 and M2 macrophage activation. PLoS ONE. 2013;8(11):e81382. 10.1371/journal.pone.0081382. 24278429 10.1371/journal.pone.0081382 PMC3835428

[30] Klopfleisch R, Jung F. The pathology of the foreign body reaction against biomaterials. J Biomed Mater Res A. 2017;105(3):927–40. 10.1002/jbm.a.35958. 27813288 10.1002/jbm.a.35958

[31] Liu YC, Zou XB, Chai YF, et al. Macrophage polarization in inflammatory diseases. Int J Biol Sci. 2014;10(5):520–9. 10.7150/ijbs.8879. 24910531 10.7150/ijbs.8879 PMC4046879

[32] Huang X, Xiu H, Zhang S, et al. The role of macrophages in the pathogenesis of ALI/ARDS. Mediators Inflamm. 2018;2018:1264913. 10.1155/2018/1264913. 29950923 10.1155/2018/1264913 PMC5989173

[33] Wynn TA, Vannella KM. Macrophages in tissue repair, regeneration, and fibrosis. Immunity. 2016;44(3):450–62. 10.1016/j.immuni.2016.02.015. 26982353 10.1016/j.immuni.2016.02.015 PMC4794754

[34] Byrne AJ, Maher TM, Lloyd CM. Pulmonary macrophages: a new therapeutic pathway in fibrosing lung disease? Trends Mol Med. 2016;22(4):303–16. 10.1016/j.molmed.2016.02.004. 26979628 10.1016/j.molmed.2016.02.004

[35] Gibbons MA, MacKinnon AC, Ramachandran P, et al. Ly6Chi monocytes direct alternatively activated profibrotic macrophage regulation of lung fibrosis. Am J Respir Crit Care Med. 2011;184(5):569–81. 10.1164/rccm.201010-1719OC. 21680953 10.1164/rccm.201010-1719OC

[36] Zhang W, Ohno S, Steer B, et al. S100a4 is secreted by alternatively activated alveolar macrophages and promotes activation of lung fibroblasts in pulmonary fibrosis. Front Immunol. 2018;9:1216. 10.3389/fimmu.2018.01216. 29910813 10.3389/fimmu.2018.01216 PMC5992816

[37] Kowal K, Silverberg JI, Lebwohl MG. CD163 and its role in inflammation. J Leukoc Biol. 2011;90(5):704–12. 10.1189/jlb.0311124.

[38] Shapouri–Moghaddam A, Mohammadian S, Vazini H, et al. Macrophage plasticity, polarization, and function in health and disease. J Cell Physiol. 2018;233(9):6425–40. 10.1002/jcp.26429. 29319160 10.1002/jcp.26429 PMC13482560

[39] Murray PJ, Wynn TA. Protective and pathogenic functions of macrophage subsets. Nat Rev Immunol. 2011;11(11):723–37. 10.1038/nri3073. 21997792 10.1038/nri3073 PMC3422549

[40] Etzerodt A, Moulin M, Doktor TK, et al. Tissue-resident macrophages in omentum promote metastatic spread of ovarian cancer. J Exp Med. 2020;217(4):e20191869. 10.1084/jem.20191869. 31951251 10.1084/jem.20191869 PMC7144521

[41] Ramachandran I, Lowther DE, Dryer–Minerly R, et al. Systemic and local immunity following adoptive transfer of NY–ESO–1 SPEAR T cells in synovial sarcoma. J Immunother Cancer. 2019;7(1):276. 10.1186/s40425-019-0762-2. 31651363 10.1186/s40425-019-0762-2 PMC6813983

[42] Zaloudíková M. Mechanisms and effects of macrophage polarization and its specifics in pulmonary environment. Physiol Res. 2023;72. 10.33549/physiolres.935058. 10.33549/physiolres.935058 PMC10660583 37565418

[43] Li K, Song Z, Yue Q, et al. Disease-specific transcriptional programs govern airway goblet cell metaplasia. Heliyon. 2024;10(13):e34105. 10.1016/j.heliyon.2024.e34105. 39071568 10.1016/j.heliyon.2024.e34105 PMC11283004

[44] Torsney E, Charlton R, Parums D, et al. Inducible expression of human endoglin during inflammation and wound healing in vivo. Inflamm Res. 2002;51(9):464–70. 10.1007/pl00012413. 12365720 10.1007/pl00012413

[45] Roh MR, Kumar R, Rajadurai A, et al. Beta-catenin causes fibrotic changes in the extracellular matrix via upregulation of collagen I transcription. Br J Dermatol. 2017;177(1):312–5. 10.1111/bjd.15079. 27639179 10.1111/bjd.15079

[46] Crosby LM, Waters CM. Epithelial repair mechanisms in the lung. Am J Physiol Lung Cell Mol Physiol. 2010;298(6):L715–31. 10.1152/ajplung.00361.2009. 20363851 10.1152/ajplung.00361.2009 PMC2886606

[47] Midwood KS, Orend G. The role of tenascin-C in tissue injury and tumorigenesis. J Cell Commun Signal. 2009;3(3–4):287–310. 10.1007/s12079-009-0075-1. 19838819 10.1007/s12079-009-0075-1 PMC2778592

[48] MacLauchlan S, Skokos EA, Meznarich N, et al. Macrophage fusion, giant cell formation, and the foreign body response require matrix metalloproteinase 9. J Leukoc Biol. 2009;85(4):617–26. 10.1189/jlb.1008588. 19141565 10.1189/jlb.1008588 PMC2656428

[49] Helming L, Gordon S. Macrophage fusion induced by IL-4 alternative activation is a multistage process involving multiple target molecules. Eur J Immunol. 2007;37(1):33–42. 10.1002/eji.200636788. 17154265 10.1002/eji.200636788

[50] Lee P, Kupeli E, Mehta AC. Airway stents. Clin Chest Med. 2010;1141–50. 10.1016/j.ccm.2009.08.002. 10.1016/j.ccm.2009.08.002 20172440

[51] Li L, Zhang X, Shi J, et al. Airway stents from now to the future: a narrative review. Respiration. 2023;102(6):439–48. 10.1159/000530421. 37232032 10.1159/000530421

[52] Zimmermann P, Ziesenitz VC, Curtis N, Ritz N. The Immunomodulatory effects of macrolides: a systematic review of the underlying mechanisms. Front Immunol. 2018;9:302. 10.3389/fimmu.2018.00302. 29593707 10.3389/fimmu.2018.00302 PMC5859047

[53] Kanoh S, Rubin BK. Mechanisms of action and clinical application of macrolides as Immunomodulatory medications. Clin Microbiol Rev. 2010;23(3):590–615. 10.1128/CMR.00078-09. 20610825 10.1128/CMR.00078-09 PMC2901655

[54] Xue C, Lin XP, Zhang JM, Chen W, Li L, Huang Y, et al. β-Elemene suppresses the proliferation of human airway granulation fibroblasts via Attenuation of TGF-β/Smad signaling pathway. J Cell Biochem. 2019;120(10):16553–66. 10.1002/jcb.28915. 31104326 10.1002/jcb.28915

