# Supplementary material for: Immunophenotypic and structural signatures of severe granulation tissue in airway implant patients
Source: Respir Res. 2026 Jan 7;27:29. doi: 10.1186/s12931-025-03480-7 (PMC12849643; doi:10.1186/s12931-025-03480-7)
Supplement: Supplementary file 1 — Supplementary Material 1. [file 12931_2025_3480_MOESM1_ESM.pdf]

Supplementary Material

Supplementary Figures and Tables

Universitätsmedizin Essen
Ruhrlandklinik

Date: ______________ **Patient ID:**

Optical scale granulation tissue

Stage 0 1 2 3 4

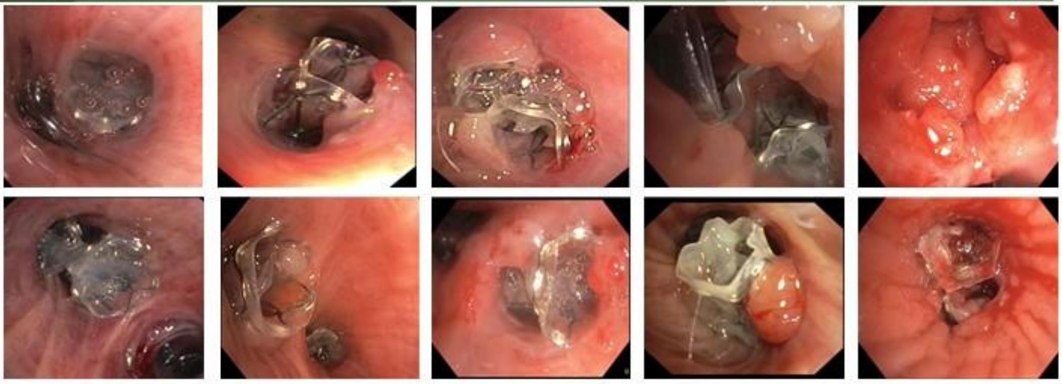

none	minimal	moderate	strong	severe
	• small polyps	• moderate polyps<br>• extensive	• strong polyps<br>• extensive granulation tissue<br>• bleeding<br>• intervention nessesary	• massive polyps<br>• Massive overgrowth bleeding<br>• Intervention nessesary

Please tick the box:

Treated lung lobe: left: ☐ L. superior ☐ L. inferior
right: ☐ L. superior ☐ L. medius ☐ L. inferior

Valve number	Localisat ion	Time from implantation	Optical scale (Stage)	Dysfunction	Dislocation
1.Index valve			☐ 0 ☐ 1 ☐ 2 ☐ 3 ☐ 4	☐ yes ☐ no	☐ yes ☐ no
2.			☐ 0 ☐ 1 ☐ 2 ☐ 3 ☐ 4	☐ yes ☐ no	☐ yes ☐ no
3.			☐ 0 ☐ 1 ☐ 2 ☐ 3 ☐ 4	☐ yes ☐ no	☐ yes ☐ no
4.			☐ 0 ☐ 1 ☐ 2 ☐ 3 ☐ 4	☐ yes ☐ no	☐ yes ☐ no
5.			☐ 0 ☐ 1 ☐ 2 ☐ 3 ☐ 4	☐ yes ☐ no	☐ yes ☐ no

Comment:

Supplementary Figure 1: Scaling sheet for the operating surgeon.

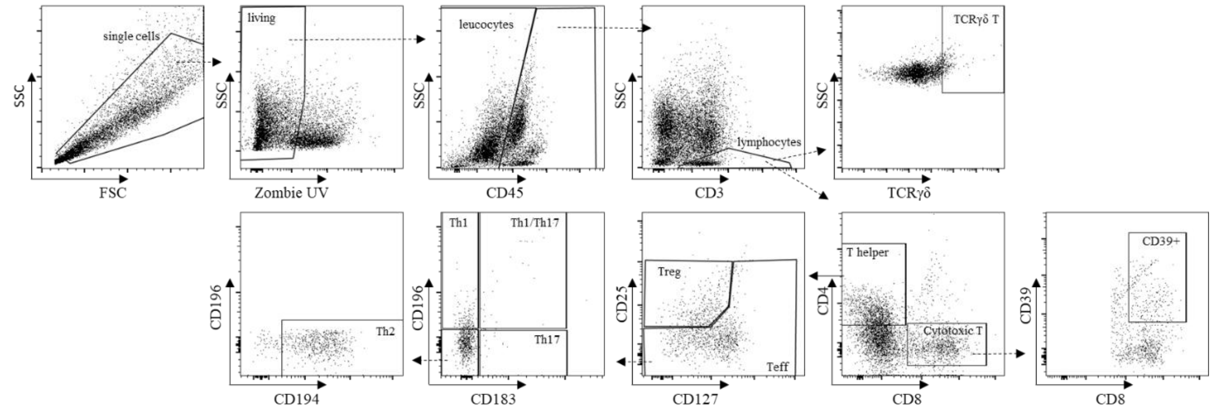

Supplementary Figure 2: Gating strategy to characterise lymphocyte subsets. Doublet exclusion was performed by plotting the height and width against the area via FSC-H/FSC-A and living cells were identified as ZombieUV$^-$ cells and leukocytes as CD45$^+$. T lymphocytes were identified as CD3$^+$ and side ward scatter (SSC) low cells. Within T lymphocytes, T helper cells were characterised as CD4$^+$ T cells and cytotoxic T cells as CD8$^+$ T cells. The TCRγδ percentage in CD3$^+$ was detected. T helper cells were further characterised as CD127^{low}CD25$^+$ regulatory T cells (Tregs) and CD127$^+$CD25$^-$ effector T cells. T helper effector cells were characterised based on the expression of CD183, CD194 and CD196 as Th1 (CD183$^+$/CD196$^-$), Th1/Th17 (CD183$^+$/CD196$^+$), Th17 (CD183$^-$/CD196$^+$) and Th2 cells (CD183$^-$/CD196$^-$/CD194$^+$) adapted from Rühle *et al.* [1,2].

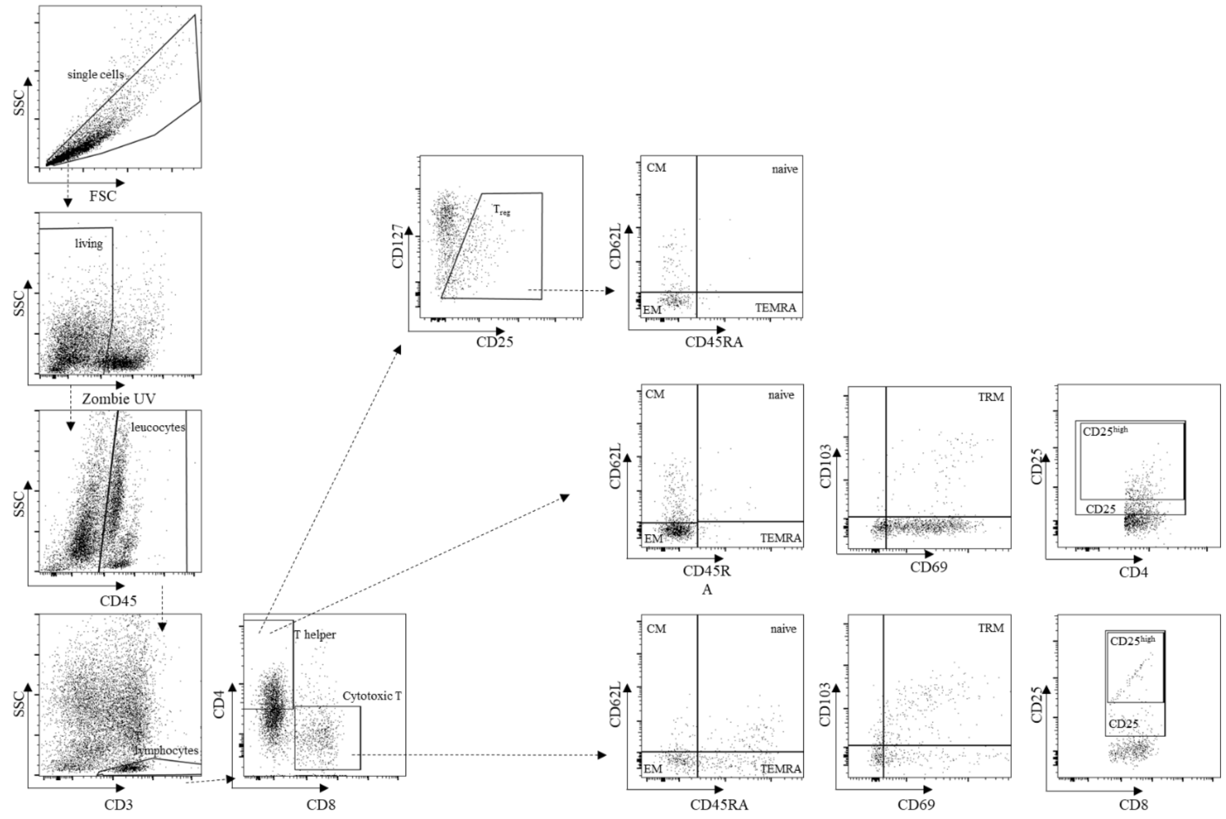

Supplementary Figure 3: Gating strategy to determine lymphocyte subsets. Doublet exclusion was performed by plotting the height and width against the area via FSC-H/FSC-A and living cells were identified as ZombieUV$^-$ cells and leukocytes as CD45$^+$.T lymphocytes were identified as CD3$^+$ and side ward scatter (SSC) low cells. Within T lymphocytes, T helper cells were characterised as CD4$^+$ T cells and cytotoxic T cells as CD8$^+$ T cells. After gating on T helper cells and cytotoxic T cells, CD45RA vs CD62L identified naïve (CD45RA$^+$/CD62L$^+$), central memory (CD45RA$^-$/CD62L$^+$), effector memory (CD45RA$^-$/CD62L$^-$), and terminal differentiated effector memory (TEMRA) (CD45RA$^+$/CD62L$^-$) CD4$^+$ or CD8$^+$ T cells. CD8 vs CD25 and CD4 vs CD25 can be used to estimate the ratio of CD8$^+$CD25$^+$, CD8$^+$CD25$^{+bright}$, CD4$^+$CD25$^+$ and CD4$^+$CD25$^{+bright}$ T cells respectively. To identify Tregs, the initial gate was placed on CD4$^+$ T cells, followed by the evaluation of CD25 vs CD127 expression; CD25$^+$CD127$^{-/dim}$ population were defined as Tregs. Further gating on these Tregs, and gating on CD45RA vs CD62L separated CD45RA$^+$/CD62L$^+$ naive- Tregs from CD45RA$^+$/CD62L$^+$ and CD45RA$^-$/CD62L$^-$ activated Tregs adapted from Jimenez *et al.* [3].

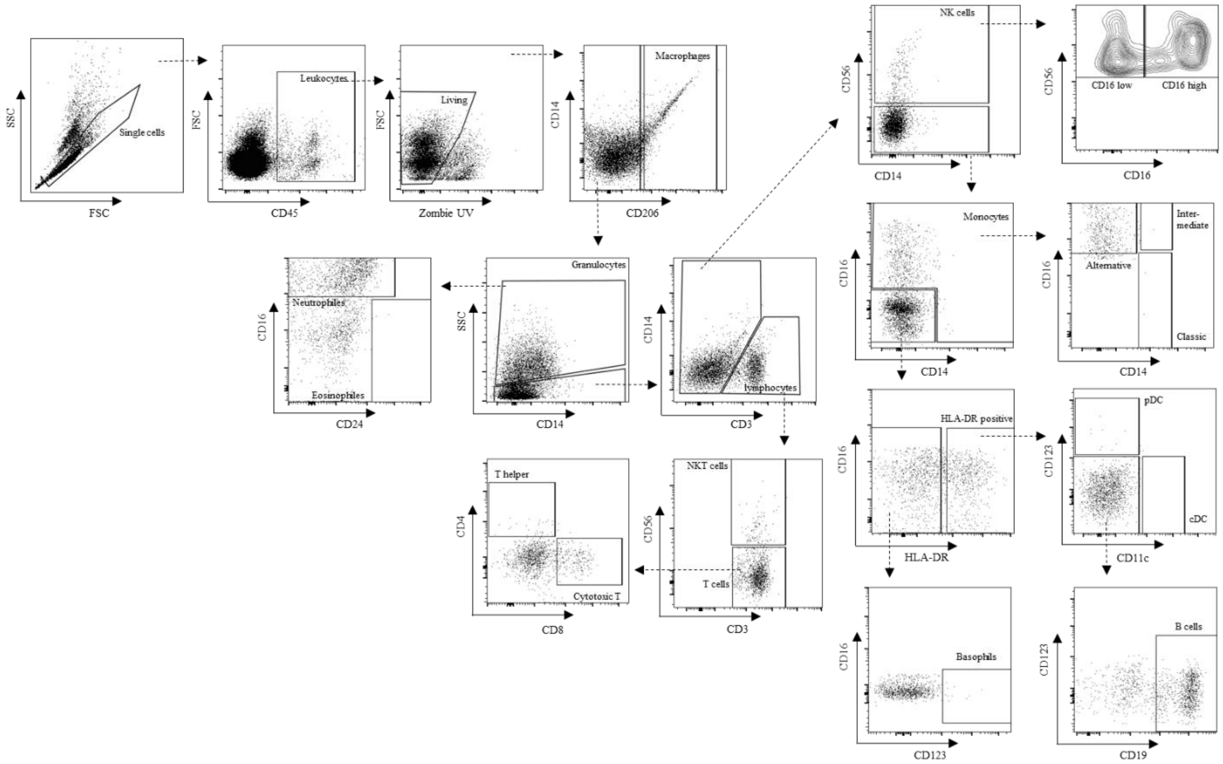

Supplementary Figure 4: Gating strategy to determine myeloid subsets. Doublets were excluded by plotting forward scatter area (FSC-A) against sideward scatter area (SSC-A). Viable cells were identified as ZombieUV$^-$negative, and leukocytes were gated as CD45-positive (CD45$^+$). Macrophages were defined as CD206$^+$/CD14$^+$ cells. Granulocytes were identified as CD14$^+$/SSC^{high} and subdivided into neutrophils (CD16$^+$/CD24$^+$) and eosinophils (CD16$^–$/CD24$^+$). Cells with low side scatter (SSC^{low}) and CD14$^+$/$^–$ expression were divided into CD3$^+$ and CD3$^–$ populations. Within the CD14$^–$/CD3$^+$ subset, NKT cells (CD3$^+$/CD56$^+$) and T cells (CD3$^+$/CD56$^–$) were identified; the latter further classified into CD4$^+$ T helper cells and CD8$^+$ cytotoxic T cells. Within the CD14$^{+/–}$/CD3$^–$ population, NK cells (CD56$^+$/CD14$^–$) were gated and differentiated as CD16^{low} and CD16^{high} NK cells. Monocytes were defined within SSC^{low}/CD14$^+$/$^–$/CD3$^–$/CD56$^–$ cells and sub classified into classical (CD14$^+$/CD16$^–$), intermediate (CD14$^+$/CD16$^+$), and alternative (CD14$^–$/CD16$^+$) monocytes. Basophils were identified within SSC^{low}/CD14$^–$/CD3$^–$/CD56$^–$/CD16$^–$/HLADR$^-$ as CD123$^+$ cells. Dendritic cells were identified within SSC^{low}/CD14$^–$/CD3$^–$/CD56$^–$/CD16$^–$/HLADR$^+$ cells as classical DCs (HLA-DR$^+$/CD11c$^+$/CD123$^–$) and plasmacytoid DCs (HLA-DR$^+$/CD11c$^–$/CD123$^+$). B cells were identified as CD19$^+$/CD123$^–$ within the HLA-DR$^+$/CD11c$^–$/CD123$^–$ population. Gating strategy adapted from Schuller *et al.* [4].

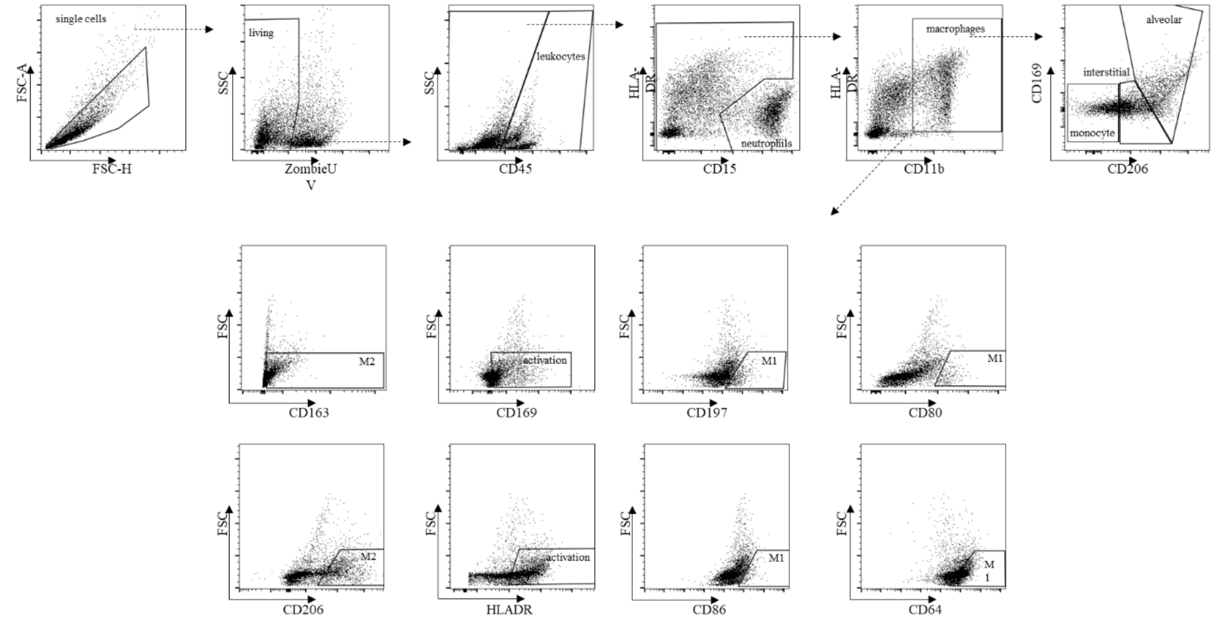

Supplementary Figure 5: Gating strategy to determine macrophage subsets and activation. Gating strategy used to identify lung monocytes and macrophages. After excluding doublets and dead cells (precautions were taken not to gate out highly auto fluorescent alveolar macrophages), cells of hematopoietic origin were identified as CD45^{+}. Neutrophils were identified as CD11b^{++}CD15^{+}HLA-DR^{-} cells and were excluded for all following analysis. We then gated on CD11b^{+}HLA-DR^{+} cells. This allowed separation from natural killer (NK) cells (CD11b^{+}HLA-DR^{-}CD56^{+}) and highly auto fluorescent eosinophils (CD11b$^{+/-}$HLA-DR^{-}Siglec 8^{+}). Finally, using CD206 and CD169, cells were separated into three subpopulations: alveolar macrophages (CD11b^{+}HLA-DR^{++}CD206^{++}CD169^{+}FSC^{high}SSC^{high}, AM), interstitial macrophages (CD11b^{+}HLA-DR^{++}CD206^{+}CD169^{-}, IM), and monocytes (CD11b^{+}HLA-DR^{+}CD206^{-}CD169^{-}, mono) [adapted from Bharat et al (5)]. In addition to Bharat et al., activation markers for macrophages were analysed. M1 macrophages were detected by CD80^{+}, CD86^{+}, CD80^{+} and CD64^{+} whereas M2 macrophages were detected by CD163^{+} and CD206$^{+.}$ CD169 and HLADR are mainly markers for general activation. The gating strategy was adapted from Bharat *et al*. [5].

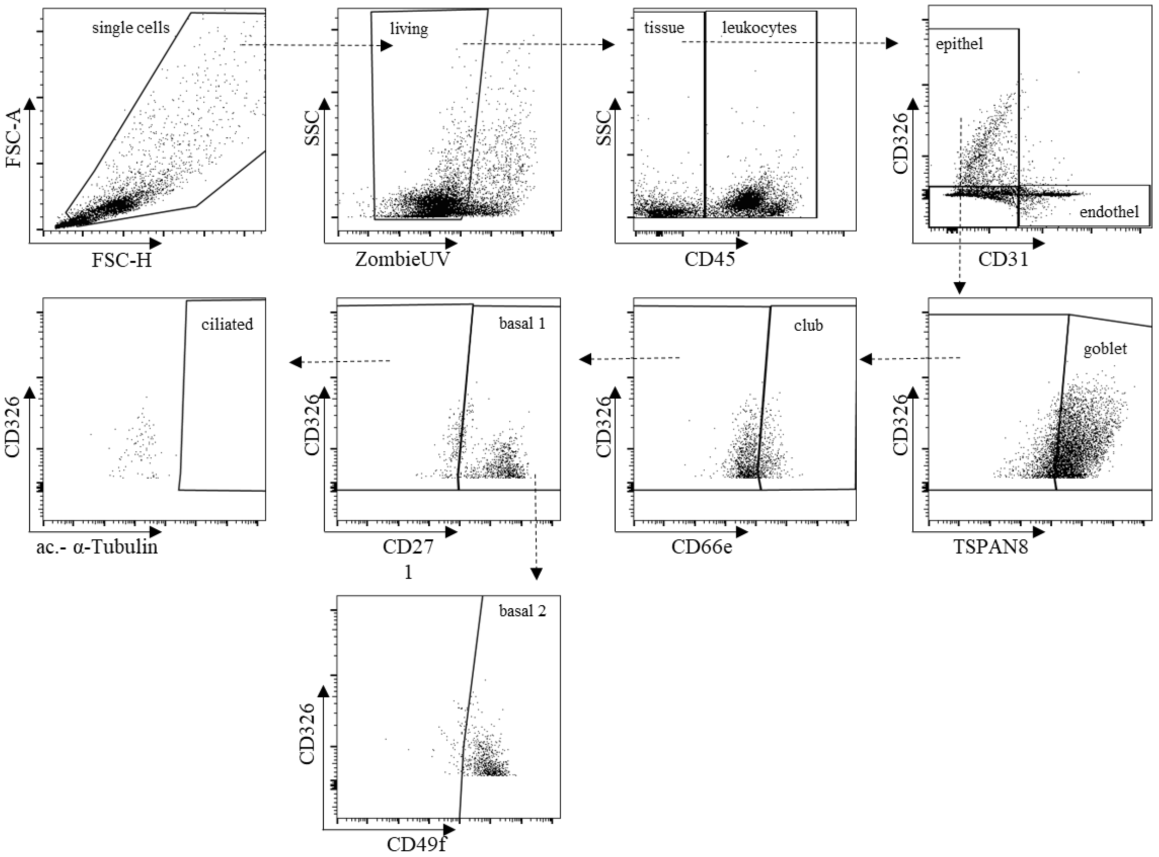

Supplementary Figure 6: Gating strategy for the differentiation of epithelial cells. Doublet exclusion was performed by plotting the height and width against the area via FSC-H/FSC-A and living cells were identified as ZombieUV$^-$ cells. CD45$^-$ cells were gated to exclude immune cells and CD326$^+$CD31$^-$ cells were defined as epithelial cells. The subpopulations of epithelial cells were distinguished using CD271$^+$/CD49f$^+$ for basal cells, CD66a/c/e$^+$ for club cells, TSPAN8$^+$ for goblet cells, and acetylated α-tubulin$^+$ for ciliated cells. The gating strategy was adapted from Bonser *et al.* [6].

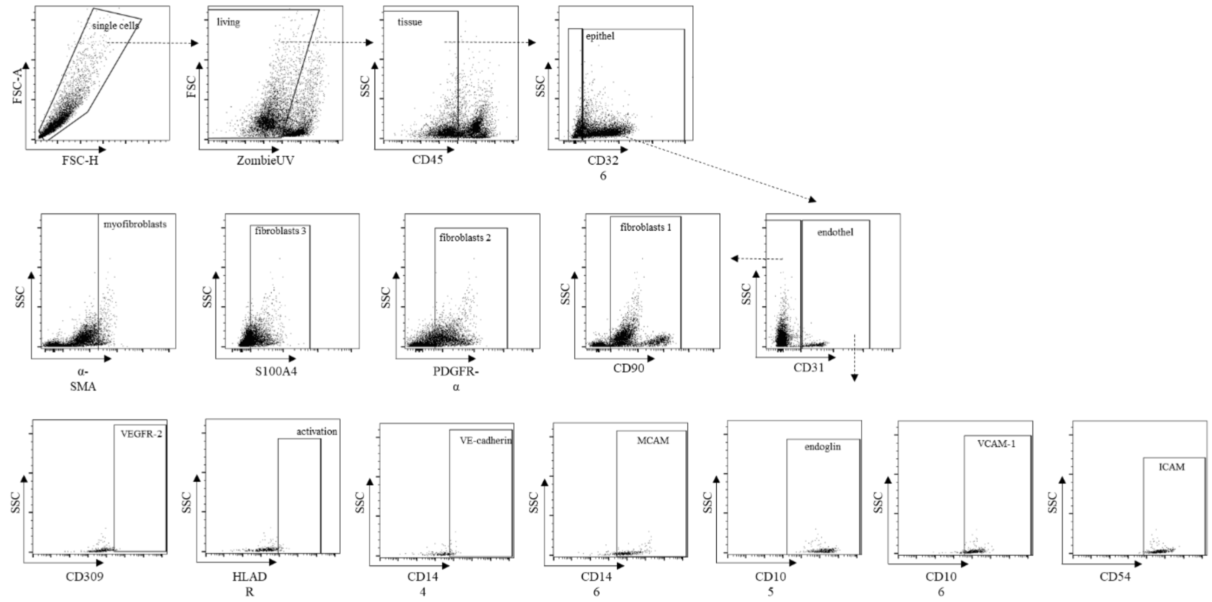

Supplementary Figure 7: Gating strategy for the activation of endothelial cells and fibroblasts. Doublet exclusion was performed by plotting the height and width against the area via FSC-H/FSC-A and living cells were identified as ZombieUV$^-$ cells. CD45$^-$ cells were gated to exclude immune cells and CD326$^+$CD31$^-$ cells were defined as epithelial cells, whereas CD326$^-$CD31$^+$ cells were identified as endothelial cells. Inside CD326$^-$CD31$^-$ cells, different fibroblast markers were analysed including CD90, PDGFR-α. S100A4 and α-SMA. The activation status of endothelial cells was analysed by CD309, HLADR, CD144, CD146, CD105, CD106 and CD54.

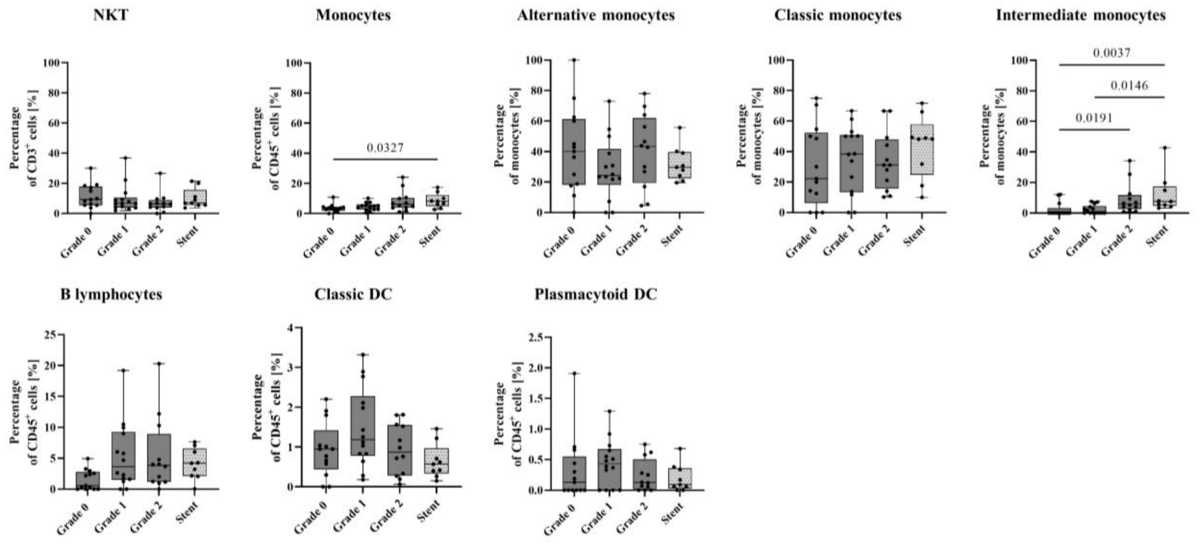

Supplementary Figure 8: Additional immunophenotyping of inflammatory cells in biopsies from valve (Grade 0, Grade 1, Grade 2) and stent patients. Percentage of natural killer T (NKT) cells in CD45$^+$. Monocytes (CD14$^+$/CD16$^+$) in CD45$^+$ cells, alternative (CD14$^-$/CD16$^+$), classic (CD14$^+$/CD16$^-$) and intermediate (CD14$^+$/CD16$^+$) in monocytes. B lymphocytes (CD19$^+$), classic (HLADR$^+$/CD11c$^+$/CD123$^-$) dendritic cells (DC) and plasmacytoid (HLADR$^+$/CD11c$^-$/CD123$^+$) DC in CD45$^+$ cells. Results are expressed as mean ± standard deviation (plot: min to max, all points). Statistical analyses were performed by Kruskal-Wallis test followed by Dunn's multiple comparisons test. Grade 0: n=13-15, Grade 1: n=13-14, Grade 2: n=12, stent: n=6-9.

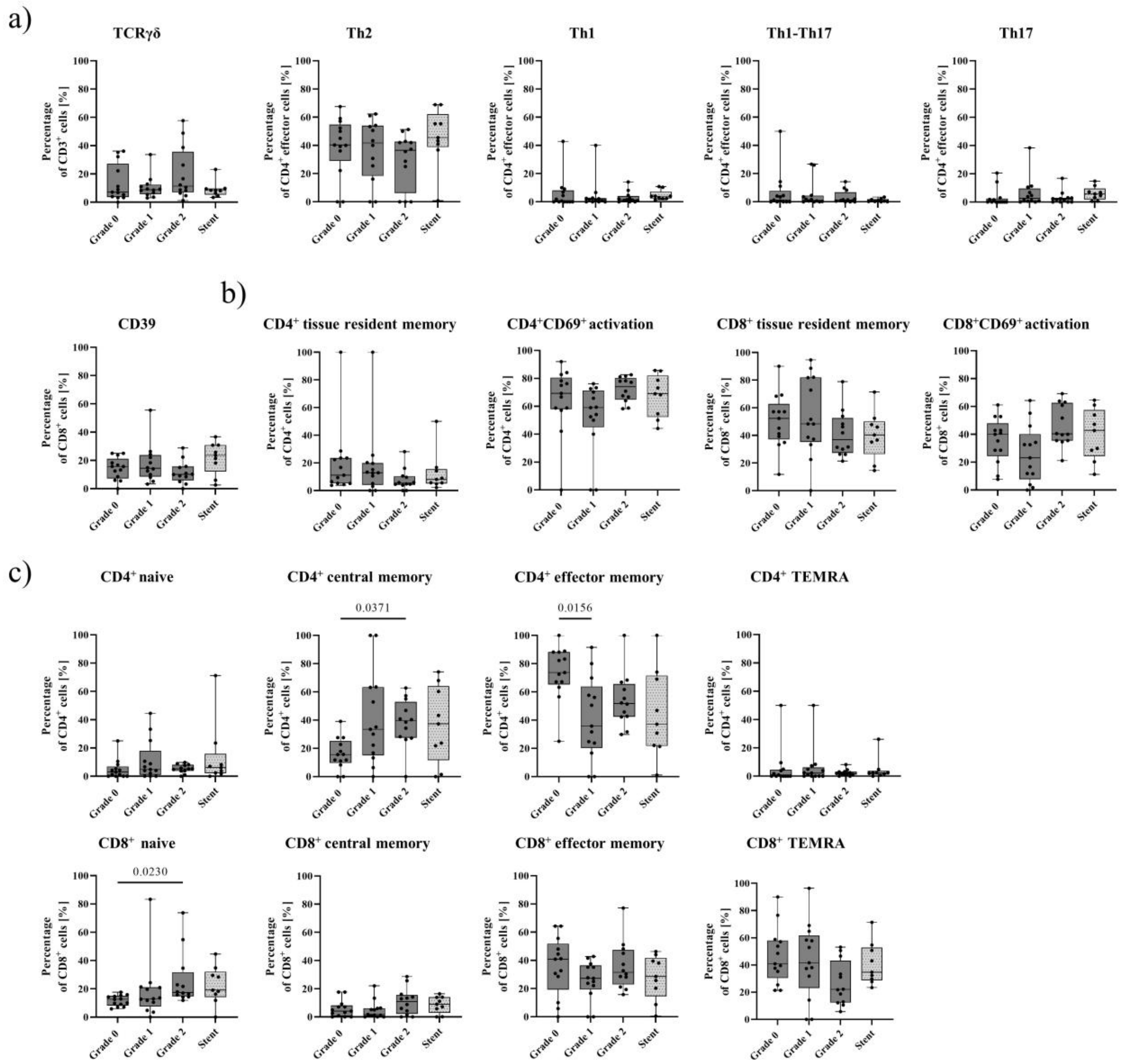

Supplementary Figure 9: Additional immunophenotyping of inflammatory cells in biopsies from valve (Grade 0, Grade 1, Grade 2) and stent patients. Percentage of a) TCRγδ in $CD3^{+}$, type 2 T helper (Th2) cells, type 1 T helper (Th1), type 1-17 T helper and type 17 T helper in effector T cells. Percentage of $CD39^{+}$ in $CD8^{+}$ cells for activation. b) Tissue resident memory ($CD69^{+}$/$CD103^{+}$) cells and T cell activation ($CD69^{+}$) in $CD4^{+}$ and $CD8^{+}$ T cells. c) Central memory ($CD45RA^{-}$/$CD62L^{+}$), effector memory ($CD45RA^{-}$/$CD62L^{-}$), naïve ($CD45RA^{+}$/$CD62L^{+}$) and terminal differentiated (TEMRA; $CD45RA^{+}$/$CD62L^{-}$) in $CD4^{+}$ T helper or cytotoxic $CD8^{+}$ T cells. Results are expressed as mean ± standard deviation (plot: min to max, all points). Statistical analyses were performed by Kruskal-Wallis test followed by Dunn's multiple comparisons test. Grade 0: n=13-15, Grade 1: n=13-14, Grade 2: n=12, stent: n=6-9.

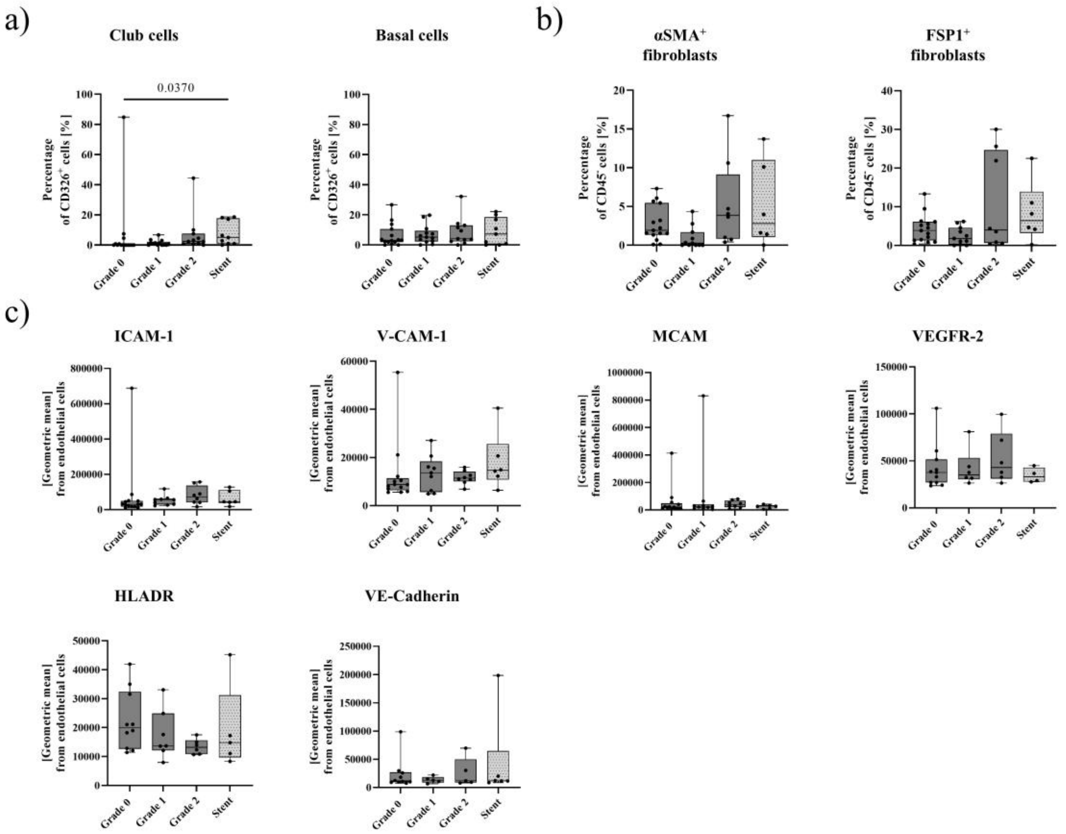

Supplementary Figure 10: Additional tissue composition of biopsies from valve (Grade 0, Grade 1, Grade 2) and stent patients analysed by flow cytometry. Percentage of a) club cells (CD66ace$^+$) and basal cells (CD271$^+$/CD49f$^+$) cells, b) fibroblasts (αSMA$^+$ or FSP1$^+$), c) the mean fluorescence intensity [geometric mean] of ICMA-1, V-CAM-1, MCAM, VEGFR-2, HLADR, and VE-Cadherin on endothelial cells. Results are expressed as mean ± standard deviation (plot: min to max, all points). Statistical analyses were performed by Kruskal-Wallis test followed by Dunn’s multiple comparisons test. Grade 0: n=13-15, Grade 1: n=13-14, Grade 2: n=12, stent: n=6-9.

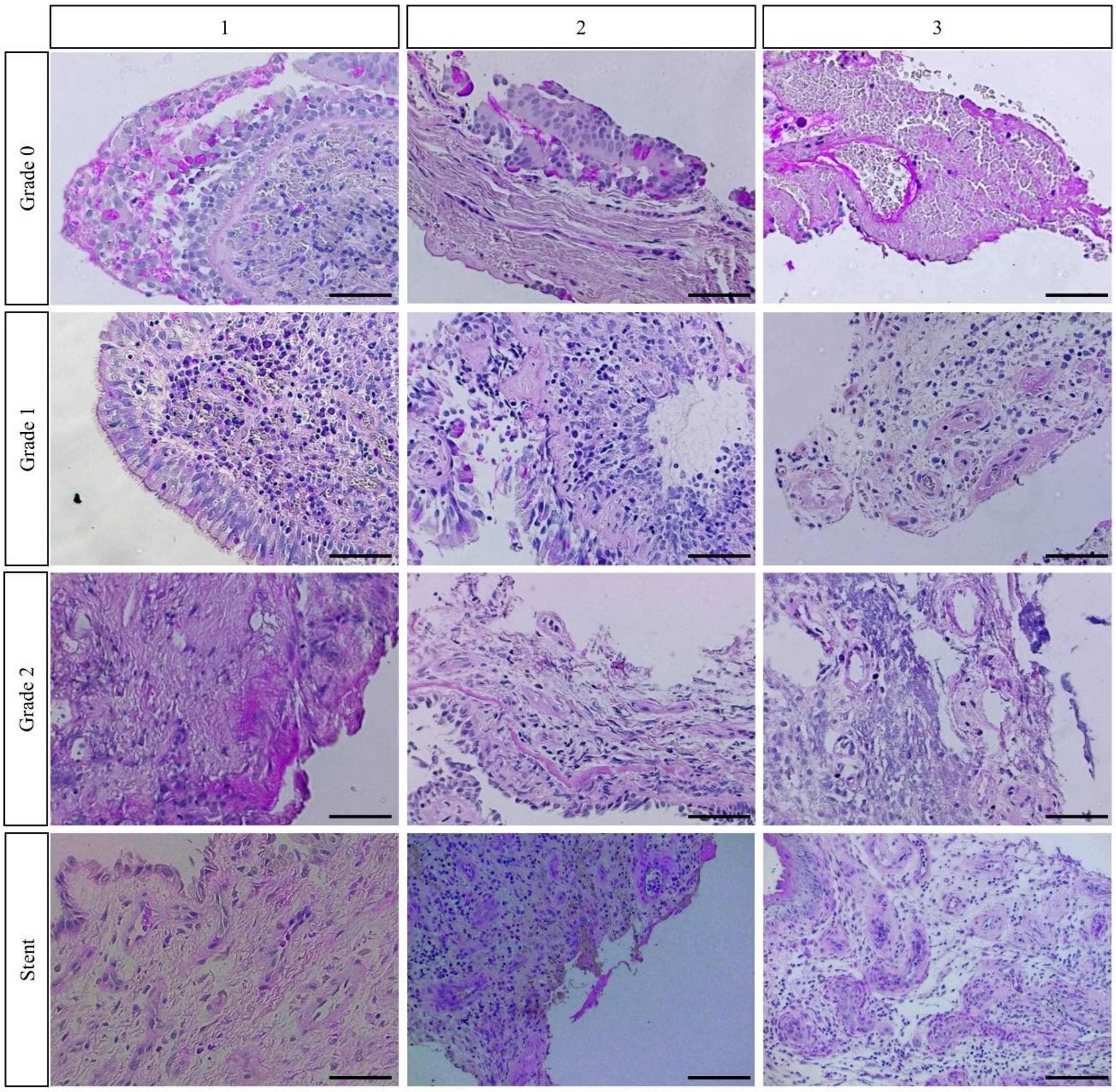

Supplementary Figure 11: Cellular and structural composition of granulation tissue biopsies. Representative periodic acid-Schiff staining of tissue from each three patients with EBV without (Grade 0), with moderate (Grade 1), and severe (Grade 2) granulation formation, as well as from patients after stent implantation. Scale bar: 150 µm; images acquired at 10× magnification.

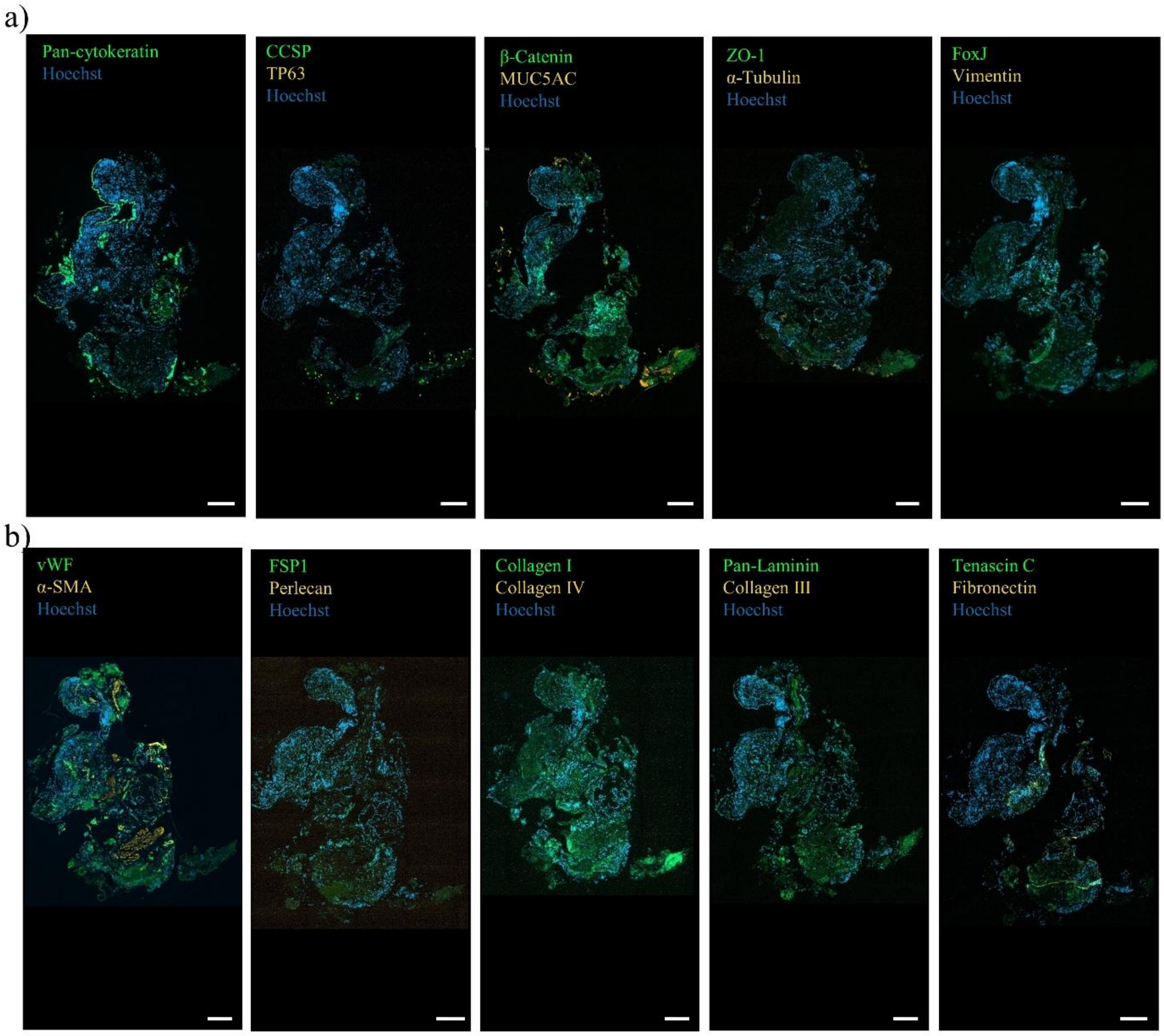

Supplementary Figure 12: Immunofluorescence staining of granulation tissue from a representative patient with valves Grade 0. a) Pan-cytokeratin (green) for epithelial cells. CCSP (green) for club cells and TP63 (orange) for basal cells. β-Catenin (green) for cellular adhesion and proliferation, and MUC5AC (orange) for goblet cells. ZO-1 (green) for tight junctions and α-Tubulin (orange) for ciliated cells. FoxJ (green) is a transcription factor for ciliogenesis and vimentin (orange) for mesenchymal cells. b) VWF (green) for endothelial cells and α-SMA for myofibroblasts or smooth muscle cells. FSP1 (green) for fibroblasts and perlecan (orange) for fibrotic tissue changes. Collagen I (green) extracellular matrix in wound healing and collagen IV (orange) for basal membrane during wound healing. Pan-laminin (green) for basal membrane during wound healing and collagen III (orange) for tissue regeneration and stiffness. Tenascin C (green) extracellular matrix in early wound healing or fibrosis and fibronectin (orange) for acute wound healing or fibrosis. In all images, cell nuclei (Hoechst 3342) are depicted in blue. Scale bar: 200 µm; images acquired at 20× magnification.

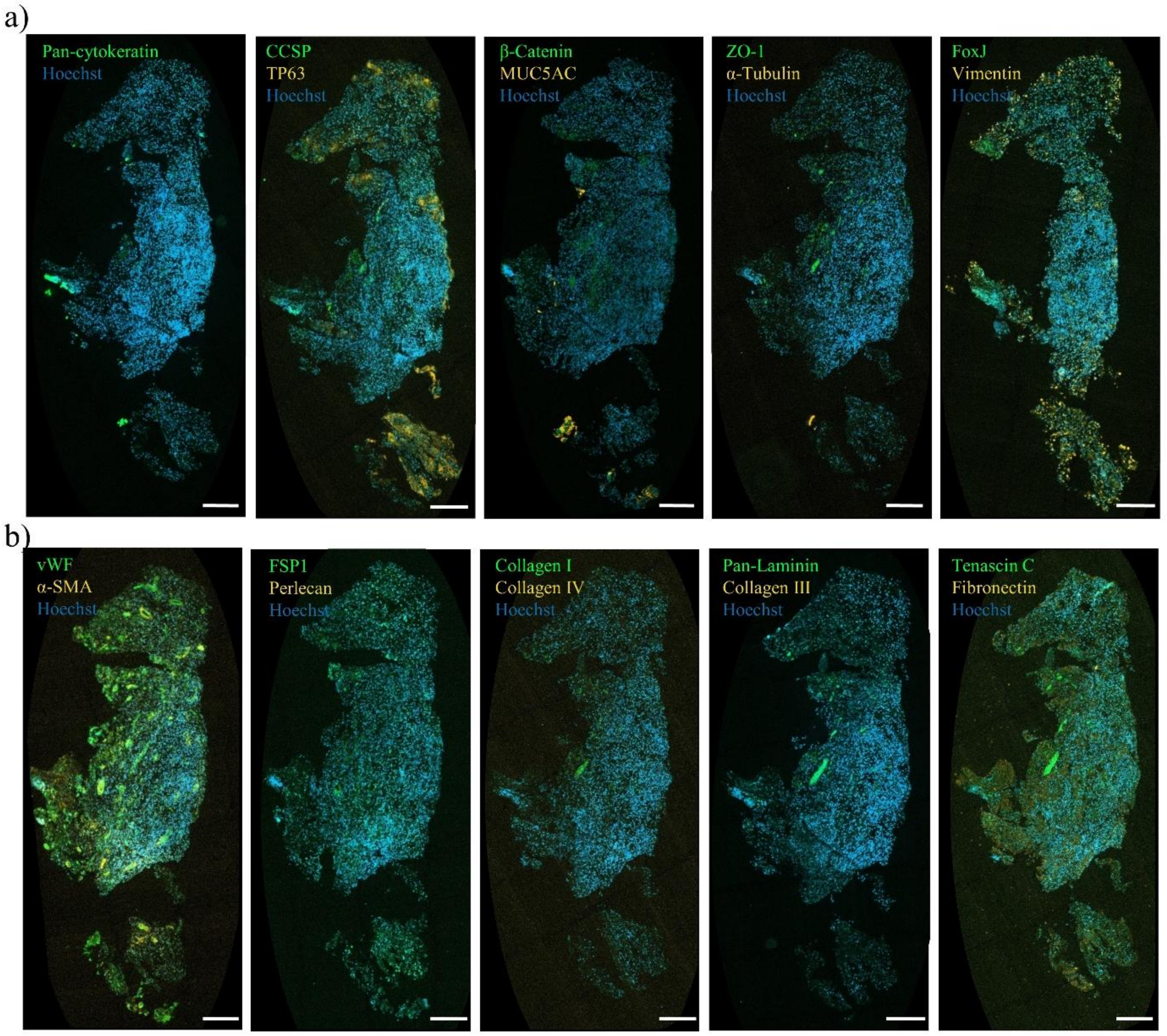

Supplementary Figure 13: Immunofluorescence staining of granulation tissue from a patient with valves Grade 1. Representative images of one Grade 1 patient are shown. a) Pan-cytokeratin (green) for epithelial cells. CCSP (green) for club cells and TP63 (orange) for basal cells. β-Catenin (green) for cellular adhesion and proliferation, and MUC5AC (orange) for goblet cells. ZO-1 (green) for tight junctions and α-Tubulin (orange) for ciliated cells. FoxJ (green) is a transcription factor for ciliogenesis and vimentin (orange) for mesenchymal cells. b) VWF (green) for endothelial cells and α-SMA for myofibroblasts or smooth muscle cells. FSP1 (green) for fibroblasts and perlecan (orange) for fibrotic tissue changes. Collagen I (green) extracellular matrix in wound healing and collagen IV (orange) for basal membrane during wound healing. Pan-laminin (green) for basal membrane during wound healing and collagen III (orange) for tissue regeneration and stiffness. Tenascin C (green) extracellular matrix in early wound healing or fibrosis and fibronectin (orange) for acute wound healing or fibrosis. In all images, cell nuclei (Hoechst 3342) are depicted in blue. Scale bar: 200 µm; images acquired at 20× magnification.

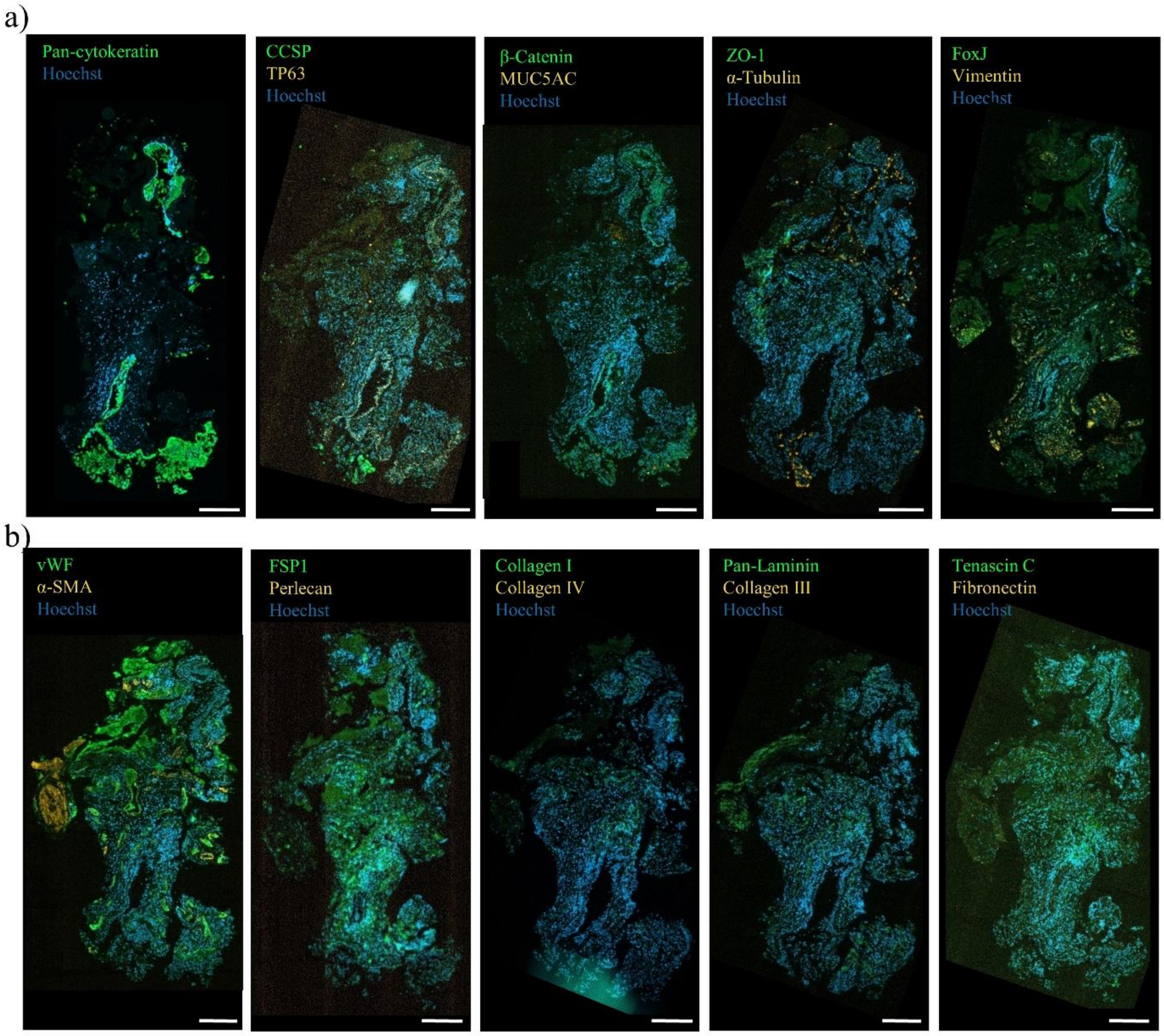

Supplementary Figure 14: Immunofluorescence staining of granulation tissue from a patient with valves Grade 2. Representative images of one Grade 2 patient are shown. a) Pan-cytokeratin (green) for epithelial cells. CCSP (green) for club cells and TP63 (orange) for basal cells. β-Catenin (green) for cellular adhesion and proliferation, and MUC5AC (orange) for goblet cells. ZO-1 (green) for tight junctions and α-Tubulin (orange) for ciliated cells. FoxJ (green) is a transcription factor for ciliogenesis and vimentin (orange) for mesenchymal cells. b) VWF (green) for endothelial cells and α-SMA for myofibroblasts or smooth muscle cells. FSP1 (green) for fibroblasts and perlecan (orange) for fibrotic tissue changes. Collagen I (green) extracellular matrix in wound healing and collagen IV (orange) for basal membrane during wound healing. Pan-laminin (green) for basal membrane during wound healing and collagen III (orange) for tissue regeneration and stiffness. Tenascin C (green) extracellular matrix in early wound healing or fibrosis and fibronectin (orange) for acute wound healing or fibrosis. In all images, cell nuclei (Hoechst 3342) are depicted in blue. Scale bar: 200 µm; images acquired at 20× magnification.

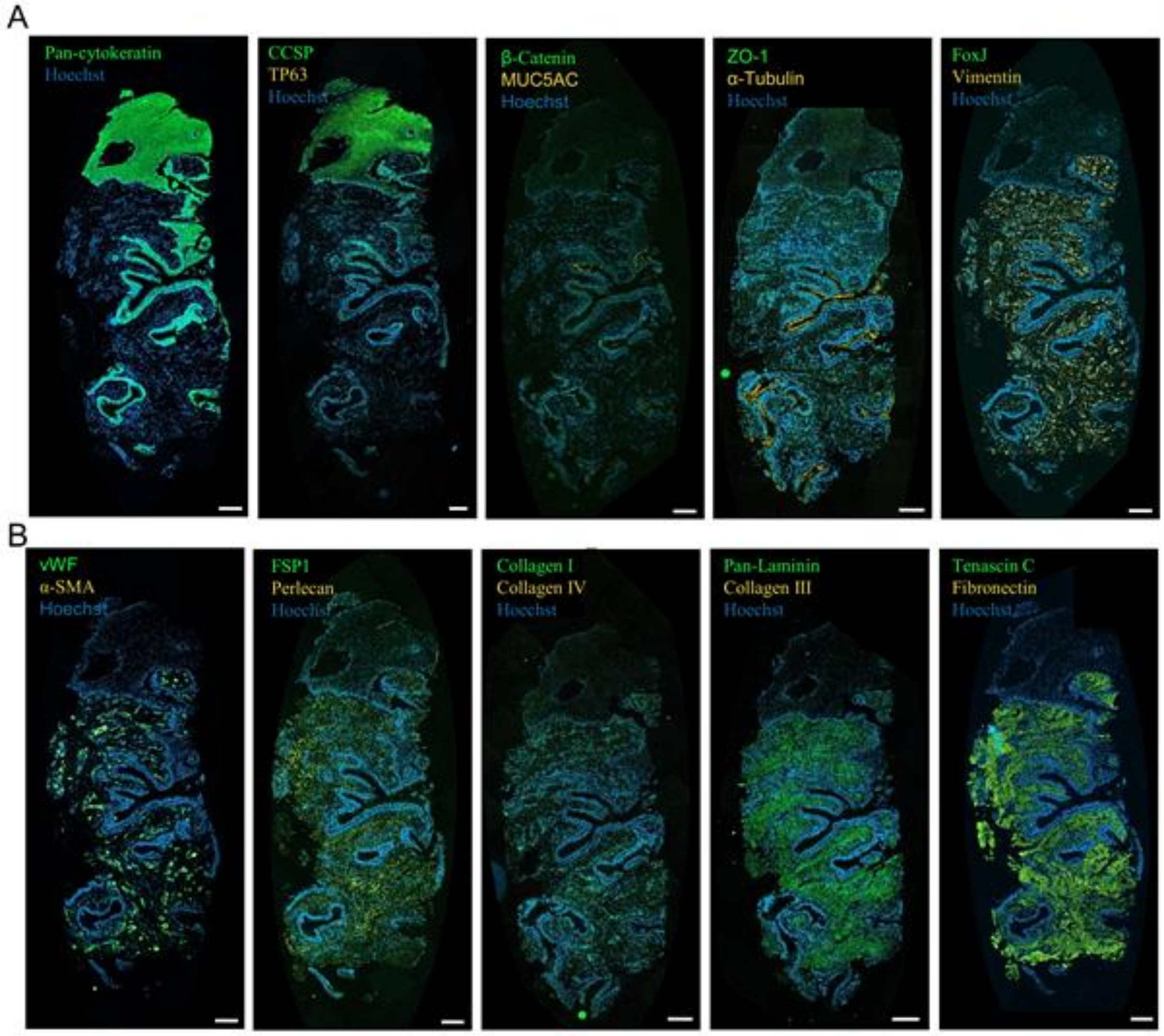

Supplementary Figure 15: Immunofluorescence staining in granulation tissue from a patient with stent implantation. Representative images of one stent patient are shown. (A) Pan-cytokeratin (green) for epithelial cells. CCSP (green) for club cells and TP63 (orange) for basal cells. β-Catenin (green) for cellular adhesion and proliferation, and MUC5AC (orange) for goblet cells. ZO-1 (green) for tight junctions and α-Tubulin (orange) for ciliated cells. FoxJ (green) is a transcription factor for ciliogenesis and vimentin (orange) for mesenchymal cells. (B) VWF (green) for endothelial cells and α-SMA for myofibroblasts or smooth muscle cells. FSP1 (green) for fibroblasts and perlecan (orange) for fibrotic tissue changes. Collagen I (green) extracellular matrix in wound healing and collagen IV (orange) for basal membrane during wound healing. Pan-laminin (green) for basal membrane during wound healing and collagen III (orange) for tissue regeneration and stiffness. Tenascin C (green) extracellular matrix in early wound healing or fibrosis and fibronectin (orange) for acute wound healing or fibrosis. In all images, cell nuclei (Hoechst 3342) are depicted in blue. Scale bar: 200 µm; images acquired at 20× magnification.

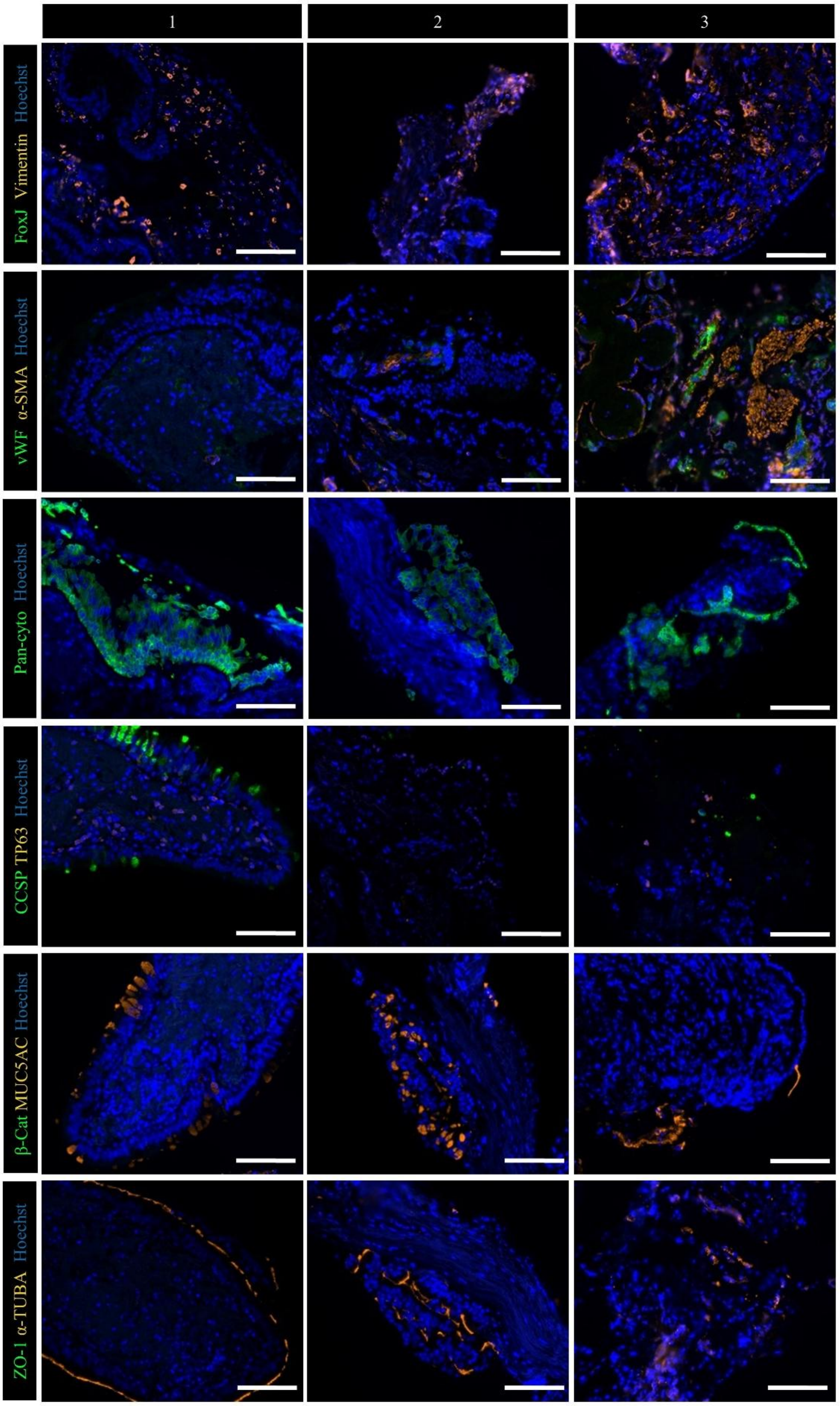

Supplementary Figure 16: Immunofluorescence staining of granulation tissue from three patient with valves Grade 0. FoxJ (green) is a transcription factor for ciliogenesis and vimentin (orange) for mesenchymal cells. VWF (green) for endothelial cells and α-SMA for myofibroblasts or smooth muscle cells. Pan-cytokeratin (green) for epithelial cells. CCSP (green) for club cells and TP63 (orange) for basal cells. β-Catenin (green) for cellular adhesion

and proliferation, and MUC5AC (orange) for goblet cells. ZO-1 (green) for tight junctions and α-Tubulin (orange) for ciliated cells. In all images, cell nuclei (Hoechst 3342) are depicted in blue. Scale bar: 100 µm; images acquired at 20× magnification.

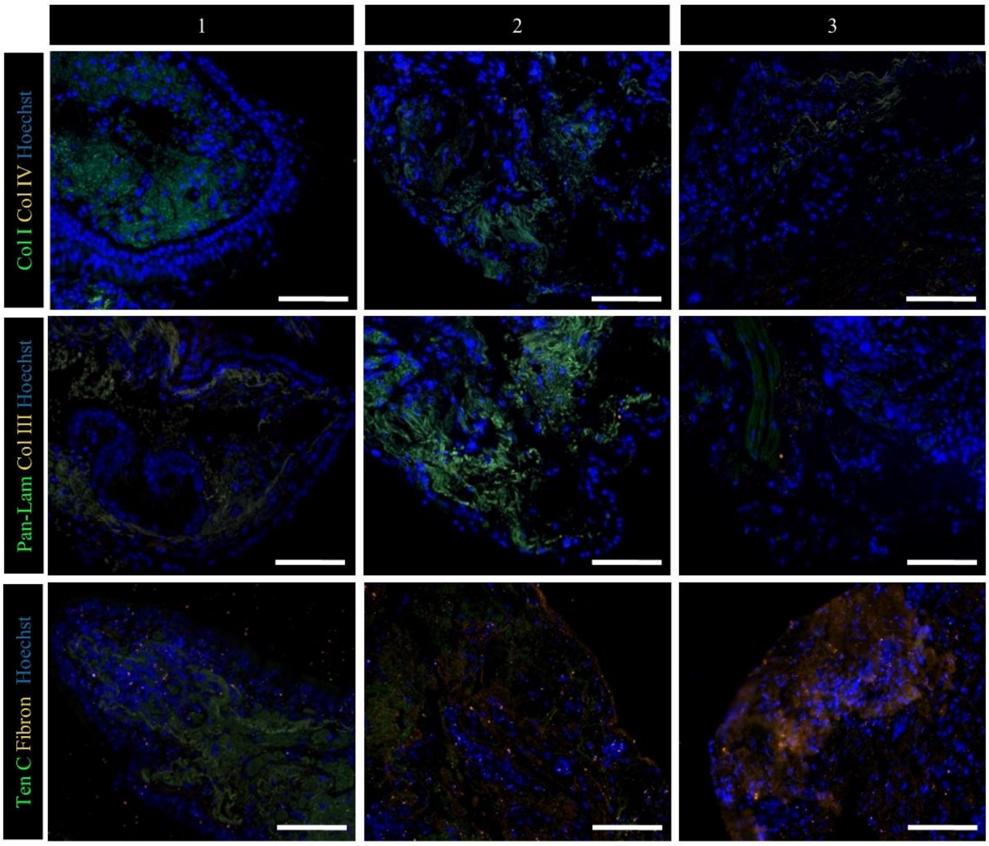

Supplementary Figure 17: Immunofluorescence staining of granulation tissue from three patient with valves Grade 0. Collagen I (green) extracellular matrix in wound healing and collagen IV (orange) for basal membrane during wound healing. Pan-laminin (green) for basal membrane during wound healing and collagen III (orange) for tissue regeneration and stiffness. Tenascin C (green) extracellular matrix in early wound healing or fibrosis and fibronectin (orange) for acute wound healing or fibrosis. In all images, cell nuclei (Hoechst 3342) are depicted in blue. Scale bar: 100 µm; images acquired at 20× magnification.

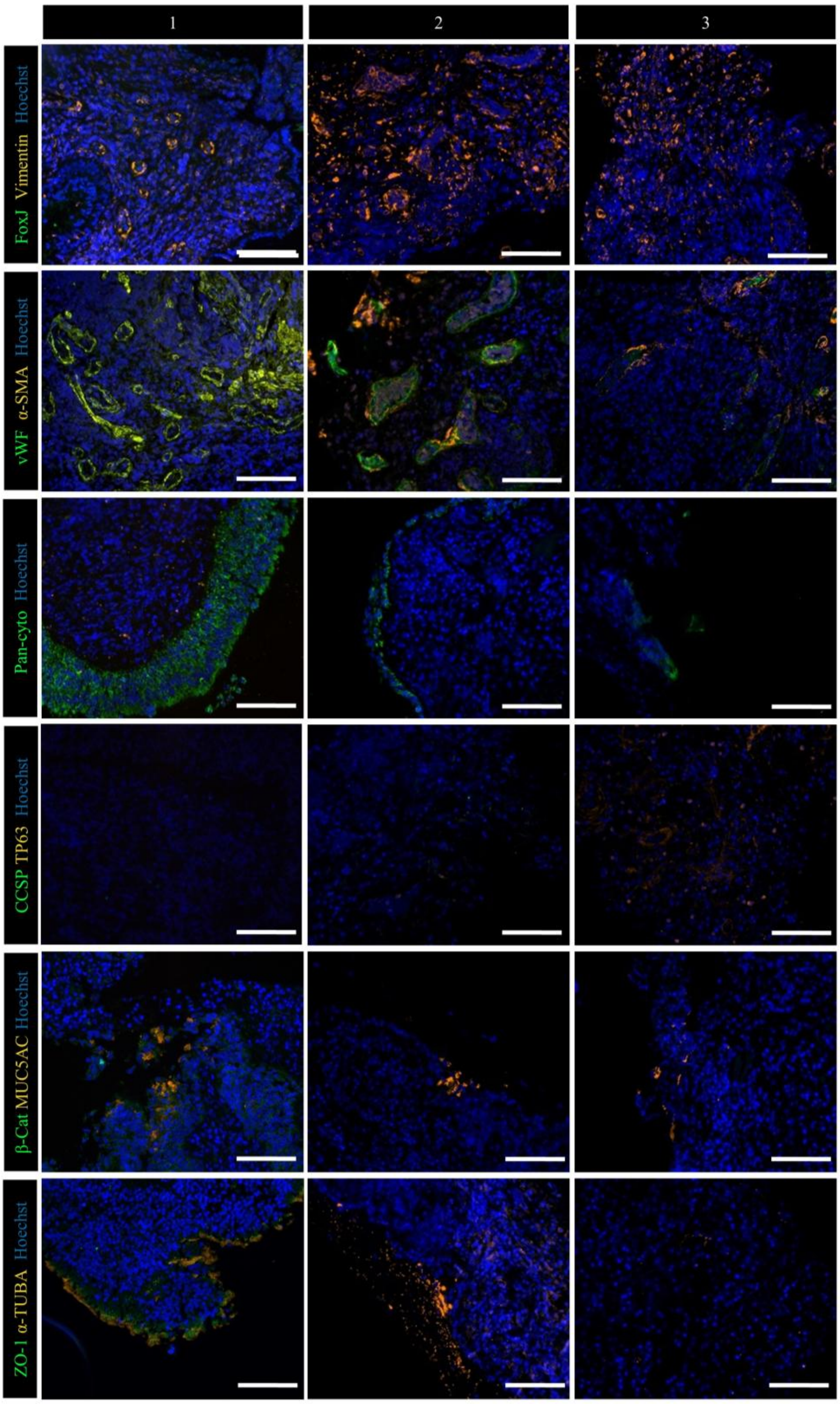

Supplementary Figure 18: Immunofluorescence staining of granulation tissue from three patient with valves Grade 1. FoxJ (green) is a transcription factor for ciliogenesis and vimentin (orange) for mesenchymal cells. VWF (green) for endothelial cells and α-SMA for myofibroblasts or smooth muscle cells. Pan-cytokeratin (green) for epithelial cells. CCSP (green) for club cells and TP63 (orange) for basal cells. β-Catenin (green) for cellular adhesion and proliferation, and MUC5AC (orange) for goblet cells. ZO-1 (green) for tight junctions and α-Tubulin (orange) for ciliated cells. In all images, cell nuclei (Hoechst 3342) are depicted in blue. Scale bar: 100 µm; images acquired at 20× magnification.

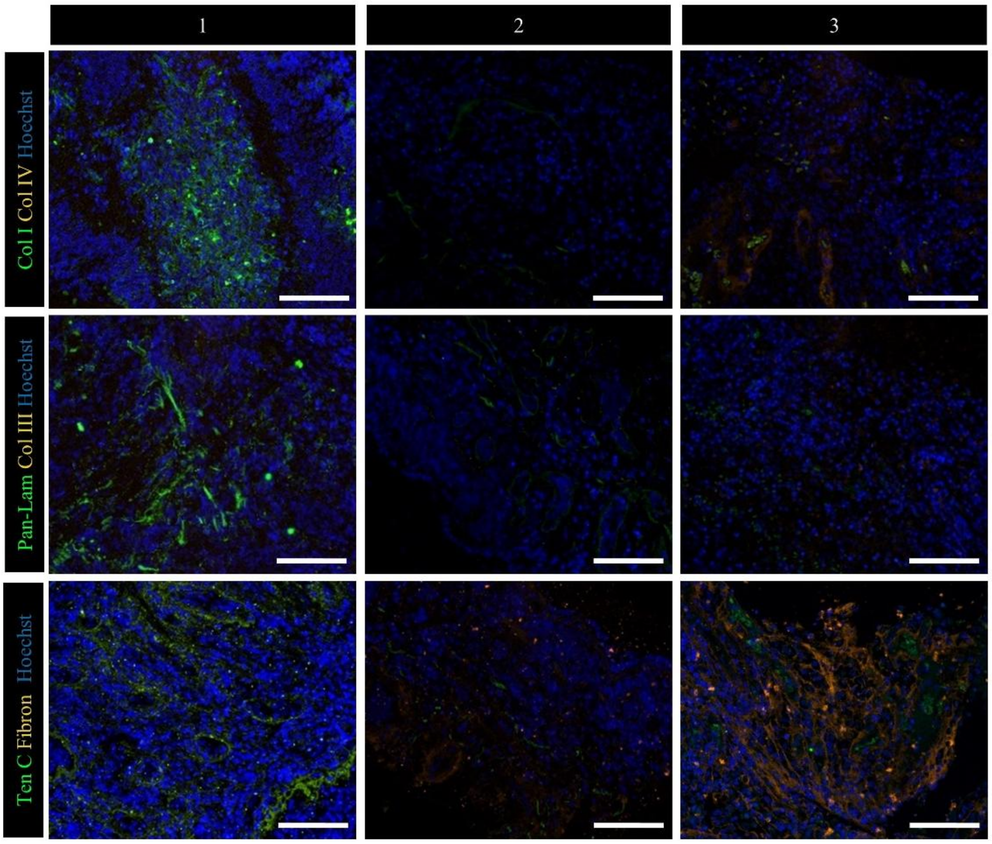

Supplementary Figure 19: Immunofluorescence staining of granulation tissue from three patient with valves Grade 1. Collagen I (green) extracellular matrix in wound healing and collagen IV (orange) for basal membrane during wound healing. Pan-laminin (green) for basal membrane during wound healing and collagen III (orange) for tissue regeneration and stiffness. Tenascin C (green) extracellular matrix in early wound healing or fibrosis and fibronectin (orange) for acute wound healing or fibrosis. In all images, cell nuclei (Hoechst 3342) are depicted in blue. Scale bar: 100 µm; images acquired at 20× magnification.

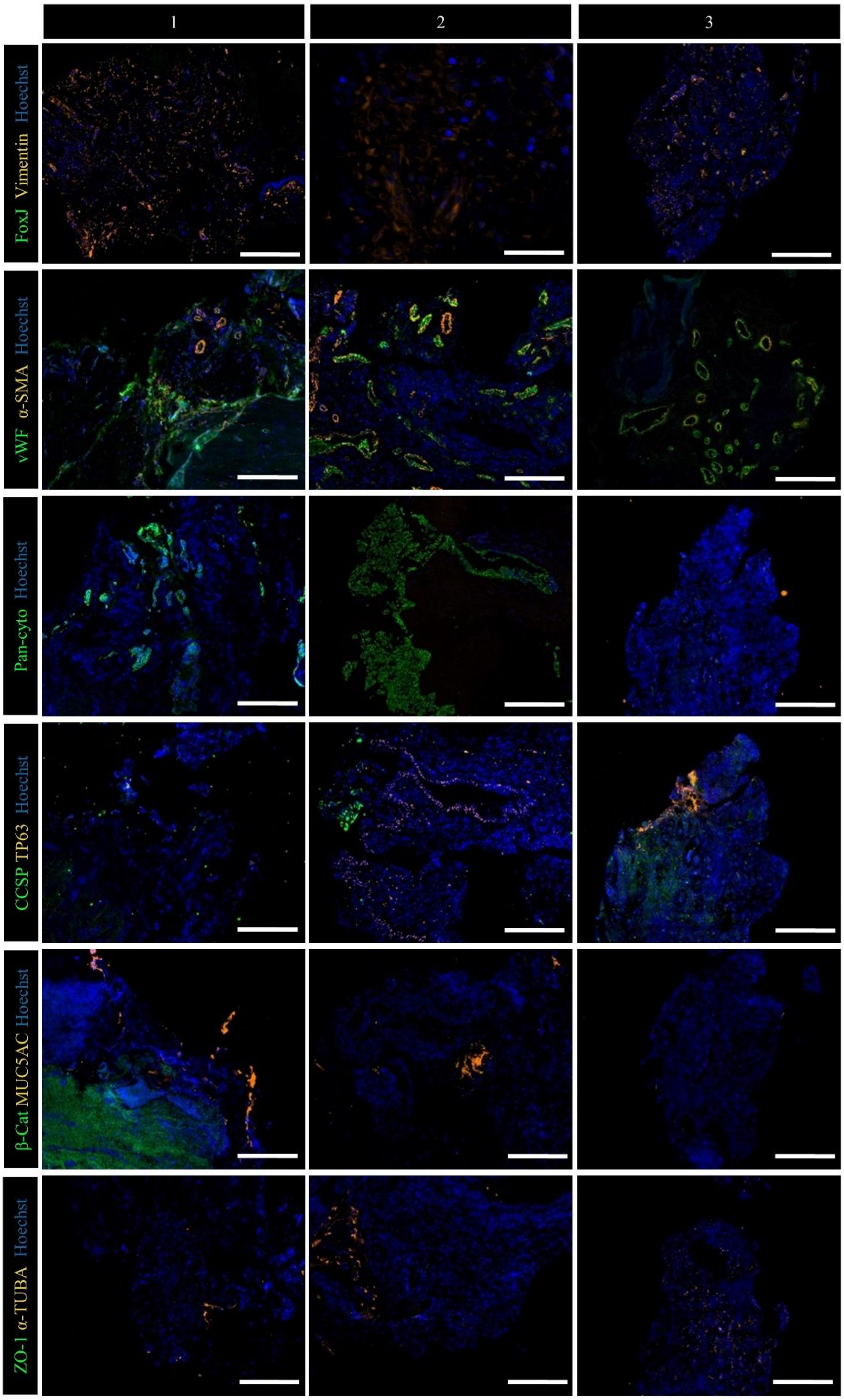

Supplementary Figure 20: Immunofluorescence staining of granulation tissue from three patient with valves Grade 2. FoxJ (green) is a transcription factor for ciliogenesis and vimentin (orange) for mesenchymal cells. VWF (green) for endothelial cells and α-SMA for myofibroblasts or smooth muscle cells. Pan-cytokeratin (green) for epithelial cells. CCSP (green) for club cells and TP63 (orange) for basal cells. β-Catenin (green) for cellular adhesion

and proliferation, and MUC5AC (orange) for goblet cells. ZO-1 (green) for tight junctions and α-Tubulin (orange) for ciliated cells. In all images, cell nuclei (Hoechst 3342) are depicted in blue. Scale bar: 100 µm; images acquired at 20× magnification.

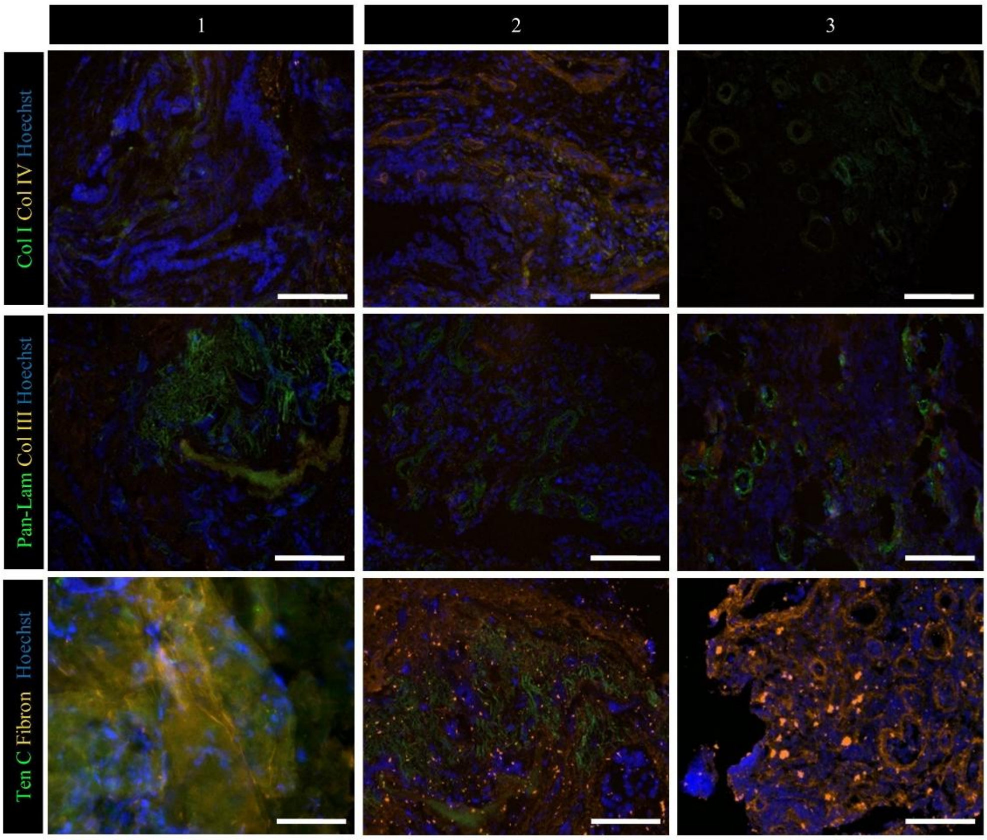

Supplementary Figure 21: Immunofluorescence staining of granulation tissue from three patient with valves Grade 2. Collagen I (green) extracellular matrix in wound healing and collagen IV (orange) for basal membrane during wound healing. Pan-laminin (green) for basal membrane during wound healing and collagen III (orange) for tissue regeneration and stiffness. Tenascin C (green) extracellular matrix in early wound healing or fibrosis and fibronectin (orange) for acute wound healing or fibrosis. In all images, cell nuclei (Hoechst 3342) are depicted in blue. Scale bar: 100 µm; images acquired at 20× magnification.

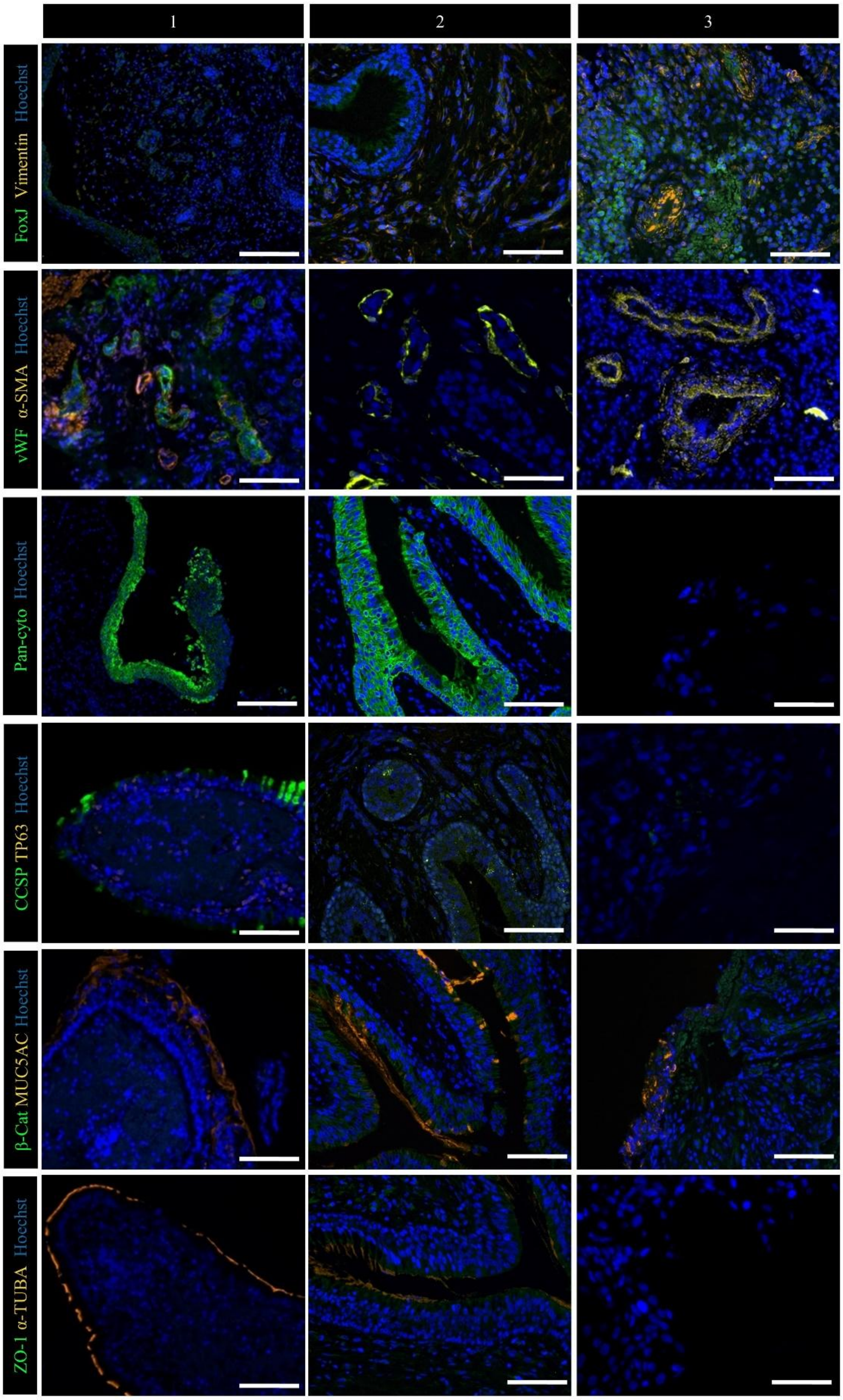

Supplementary Figure 22: Immunofluorescence staining of granulation tissue from three patients with stent. FoxJ (green) is a transcription factor for ciliogenesis and vimentin (orange) for mesenchymal cells. VWF (green) for endothelial cells and α-SMA for myofibroblasts or smooth muscle cells. Pan-cytokeratin (green) for epithelial cells. CCSP (green) for club cells and TP63 (orange) for basal cells. β-Catenin (green) for cellular adhesion and proliferation, and MUC5AC (orange) for goblet cells. ZO-1 (green) for tight junctions and α-Tubulin (orange) for

ciliated cells. In all images, cell nuclei (Hoechst 3342) are depicted in blue. Scale bar: 100 µm; images acquired at 20× magnification.

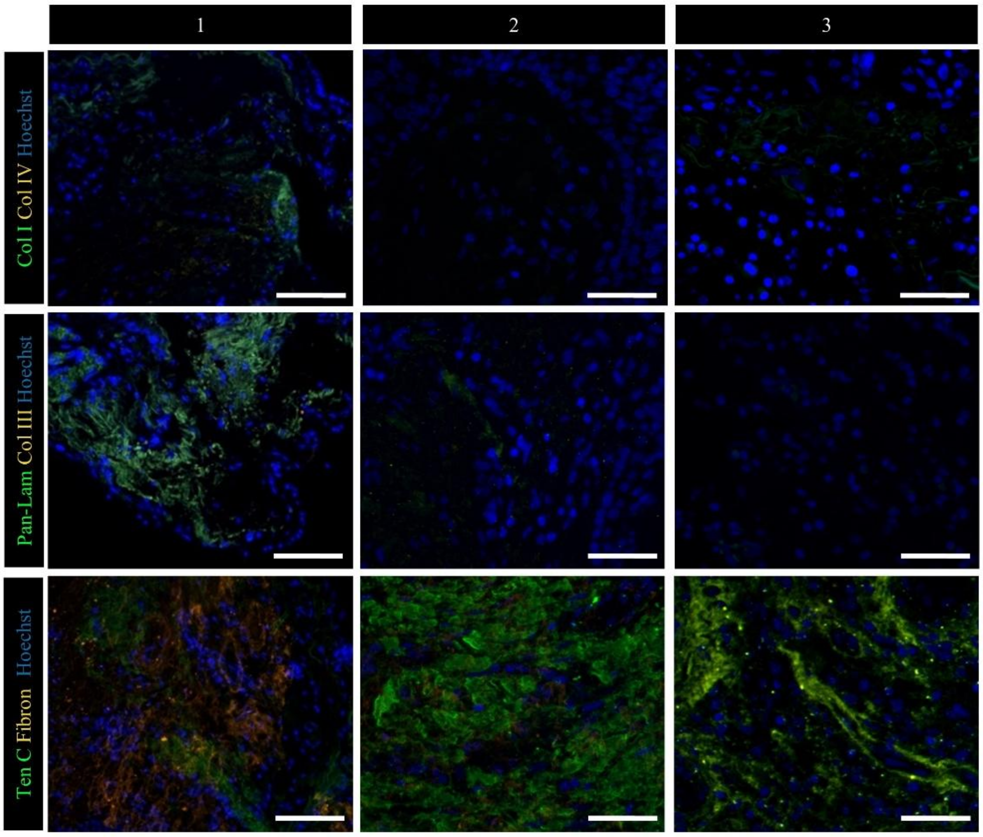

Supplementary Figure 23: Immunofluorescence staining of granulation tissue from three patients with stent. Collagen I (green) extracellular matrix in wound healing and collagen IV (orange) for basal membrane during wound healing. Pan-laminin (green) for basal membrane during wound healing and collagen III (orange) for tissue regeneration and stiffness. Tenascin C (green) extracellular matrix in early wound healing or fibrosis and fibronectin (orange) for acute wound healing or fibrosis. In all images, cell nuclei (Hoechst 3342) are depicted in blue. Scale bar: 100 µm; images acquired at 20× magnification.

Supplementary Table 1: Antibodies for characterization of T cell subpopulations by flow cytometry.

**Epitope**	**Function**	**Dilution**	**Fluorochrome**	**Company (order number)**
ZombieUV	Live/dead	1: 1000	(NUV405)	Biolegend (423107)
Protein tyrosine phosphatase receptor type C (PTPRC, CD45)	Leukocytes	1:500	AF488AF488	Biolegend (36853)
CD4	T helper cells	1:200	PerCP Cy5.5	Biolegend (300530)
T cell receptor gamma delta (TCRyd)	Gamma (γ) chain and delta (δ) chain	1:200	PeCy7	Biolegend (331222)
C-C chemokine receptor type 4 (CCR4, CD194)	T helper 2 cells	1:200	Pe-Dazzle	Biolegend (359419)
Interleukin-7 receptor alpha (IL-7Rα, CD127)	Naive T cells $CD127^{++}$, regulatory T cells $CD127^{+-}$	1:200	APC	Biolegend (351316)
CD3	T cells	1:200	AF700	Biolegend (344822)
C-C chemokine receptor type 7 (CCR7, CD197)	Naive T cells and central memory T cells	1:200	APC/Fire750	Biolegend (353246)
Interleukin-2 receptor subunit alpha (IL-2Rα, CD25)	Regulatory T cells	1:200	BV421	Biolegend (368536)
CD8 glycoprotein (CD8)	Cytotoxic T cells	1:200	BV510	Biolegend (301048)
Ectonucleoside triphosphate diphosphohydrolase-1 (ENTPD1, CD39)	Regulatory T cells	1:200	BV605	Biolegend (328236)
C-C chemokine receptor type 6 (CCR6, CD196)	T helper 17 cells	1:200	BV650	Biolegend (353442)
C-X-C chemokine receptor type 3 (CXCR3, CD183)	T helper 1 cells	1:200	BV785	Biolegend (353738)
C-C chemokine receptor type 10 (CCR10)	Memory T cells	1:200	PE	Biolegend (341503)

Cluster of differentiation (CD)

Supplementary Table 2: Antibodies for characterization of T cell activation and memory by flow cytometry.

**Epitope**	**Function**	**Dilution**	**Fluorochrome**	**Company (order number)**
ZombieUV	Dead	1:1000	(NUV405)	Biolegend (423107)
Protein tyrosine phosphatase receptor type C (PTPRC, CD45)	Leukocytes	1:500	AF488	Biolegend (36853)
CD4	T helper cells	1:200	PerCP Cy5.5	Biolegend (300530)
AIM (Activation-Induced Molecule, CD69)	Tissue-resident memory T cells (TRM)	1:200	PeCy7	Biolegend (310911)
Integrin alpha E (αE, CD103)	Tissue-resident memory T cells (TRM)	1:200	Pe-Dazzle	Biolegend (350224
L-selectin (CD62L)	Naive T cells and central memory T cells	1:200	PE	Biolegend (304806)
Interleukin-7 receptor alpha (IL-7Rα, CD127)	Naive T cells $CD127^{++}$, regulatory T cells $CD127^{+-}$	1:200	APC	Biolegend (351316)
CD3	T cells	1:200	AF700	Biolegend (344822)
C-C chemokine receptor type 7 (CCR7, CD197)	Naive T cells and central memory T cells	1:200	APC/Fire750	Biolegend (353246)
Interleukin-2 receptor subunit alpha (IL-2Rα, CD25)	Regulatory T cells	1:200	BV421	Biolegend (302630)
CD8 glycoprotein (CD8)	Cytotoxic T cells	1:200	BV510	Biolegend (301048)
CD45 isoform A (CD45RA)	Naive T cells	1:200	BV605	Biolegend (304134)

Cluster of differentiation (CD)

Supplementary Table 3: Antibodies for characterization of myeloid cells by flow cytometry.

Epitope	Function	Dilution	Fluorochrome	Company (order number)
ZombieUV	Dead	1:1000	(NUV405)	Biolegend (423107)
Protein tyrosine phosphatase receptor type C (PTPRC, CD45)	Leukocytes	1:500	AF488	Biolegend (36853)
CEACAM8 (Carcinoembryonic Antigen-Related Cell Adhesion Molecule 8, CD66b)	Granulocytes, including neutrophils and eosinophils	1:400	PE	Biolegend (392903)
CD4	T helper cells	1:400	PerCP Cy5.5	Biolegend (300530)
Interleukin-3 receptor alpha chain (IL-3Rα, CD123)	Plasmacytoid dendritic cells	1:400	Pe-Cy5	Biolegend (306008)
B-lymphocyte antigen (CD19)	B cells	1:400	PeC7	Biolegend (302216)
Neural cell adhesion molecule (NCAM, CD56)	NK cells	1:400	Pe-Dazzle	Biolegend (318348)
Heat-stable antigen (HAS, CD24)	Granulocytes	1:200	APC	Biolegend (311117)
CD3	T cells	1:400	AF700	Biolegend (344822)
Fc gamma receptor III (FcγRIII, CD16)	Monocytes and NK cells	1:400	APC/Fire750	Biolegend (302059)
Human Leukocyte Antigen - DR (HLA-DR)	Macrophages and other APCs	1:200	BV421	Biolegend (307636)
CD8 glycoprotein (CD8)	Cytotoxic T cells	1:400	BV510	Biolegend (301048)
Mannose receptor (CD206)	Macrophages (M2)	1:200	BV605	Biolegend (321140)
Integrin alpha X (ITGAX, CD11c)	Macrophages and other APCs	1:200	BV650	Biolegend (301638)
CD14	Monocytes and macrophages	1:400	BV785	Biolegend (301839)

Cluster of differentiation (CD)

Supplementary Table 4: Antibodies for characterization of macrophage activation by flow cytometry.

Epitope	Function	Dilution	Fluorochrome	Company (order number)
ZombieUV	Dead	1:1000	(NUV405)	Biolegend (423107)
Protein tyrosine phosphatase receptor type C (PTPRC, CD45)	Leukocytes	1:500	AF488	Biolegend (36853)
CD16 (FcγRIII,)	Monocytes, makrophages, NK cells	1:200	PE	Biolegend (302059)
CD163	Monocytes and makrophages (M2)	1:200	PerCP Cy5.5	Biolegend (333608)
Lewis X, fucosyltransferase 4, CD15	Granulocytes	1:200	Pe-Cy7	Biolegend (301924)
Integrin alpha M (ITGAM, CD11b)	Monocytes, macrophages, granulocytes	1:200	Pe-Dazzle	Biolegend (301348)
CD86	Macrophages (M1), DCs, B cells	1:200	AF647	Biolegend (305416)
Fc gamma receptor I (FcγRI, CD64)	Macrophages (M1)	1:200	AF700	Biolegend (305040)
C-C chemokine receptor type 7 (CCR7, CD197)	Macrophages (M1)	1:400	APC/Fire750	Biolegend (353246)
Human Leukocyte Antigen - DR (HLA-DR)	Macrophages and other APCs	1:200	BV421	Biolegend (307636)
B7-1, CD80	Macrophages (M1)	1:200	BV510	Biolegend (305233)
Mannose receptor (CD206)	Macrophages (M2)	1:200	BV605	Biolegend (321140)
Integrin alpha X (ITGAX, CD11c)	Macrophages and other APCs	1:200	BV650	Biolegend (301638)
CD14	Monocytes and macrophages	1:400	BV785	Biolegend (301839)

Cluster of differentiation (CD)

Supplementary Table 5: Antibodies for characterization of epithelial differentiation by flow cytometry.

Epitope	Function	Dilution	Fluorochrome	Company (order number)
ZombieUV		1:1000	(NUV405)	Biolegend (423107)
Protein tyrosine phosphatase receptor type C (PTPRC, CD45)	Leukocytes	1:500	BV650	Biolegend (304044)
Epithelial cell adhesion molecule (Ep-CAM, CD326)	Epithelial cells	1:400	PerCP Cy5.5	Biolegend (324214)
Nerve growth factor receptor (NGFR, CD271)	Basal cells	1:400	Pe-Cy7	Biolegend (345110)
Integrin α6, CD49f	Basal cells	1:200	BV421	Biolegend (313623)
Thymocyte differentiation antigen-1 (Thy-1, CD90)	Fibroblasts	1:200	BV510	Biolegend (328126)
Carcinoembryonic antigen-related cell adhesion molecule 1/6/5, (CEACAM1/6/5/, CD66ace)	Club cells	1:200	BV605	Biolegend (342324)
Platelet endothelial cell adhesion molecule (PECAM-1, CD31)	Endothelial cells	1:200	BV785	Biolegend (303148)
Tetraspanin-8 (TSPAN8)	Goblet cell	1:400	APC	Biolegend (363706)

Cluster of differentiation (CD)

Supplementary Table 6: Antibodies for characterization of fibroblast and endothelial activation by flow cytometry.

Epitope	Function	Dilution	Fluorochrome	Company (order number)
ZombieUV	Dead	1:1000	(NUV405)	Biolegend (423107)
Vascular endothelial growth factor receptor-2 (VEGFR-2, CD309)	Endothelial cells (angiogenesis and vascular permeability)	1:200	BV421	Biolegend (393010)
Thymocyte differentiation antigen-1 (Thy-1, CD90)	Fibroblasts (tissue remodelling and repair processes, fibrosis)	1:200	BV510	Biolegend (328126)
Human leukocyte antigen-DR (HLADR)	Endothelial activation	1:200	BV605	Biolegend (307640)
Protein tyrosine phosphatase receptor type C (PTPRC, CD45)	Leukocytes	1:400	BV650	Biolegend (304044)
Platelet endothelial cell adhesion molecule (PECAM-1, CD31)	Endothelial cells (vascular integrity)	1:200	BV785	Biolegend (303148)
Epithelial cell adhesion molecule (Ep-CAM, CD326)	Epithelial cells	1:400	PerCP Cy5.5	Biolegend (324214)
Platelet derived growth factor receptor alpha (CD140a)	Fibroblasts (proliferation, ECM-production, activation to myofibroblasts, lung fibrosis)	1:200	PE	Biolegend (323506)
Vascular endothelial cadherin (VE-cadherin, CD144)	Endothelial cells (integrity endothelial barrier)	1:200	PE/Dazzle	Biolegend (348520)
Vascular cell adhesion molecule 1 (VCAM-1, CD106)	Endothelial cells (interaction with leukocytes, fibrosis)	1:200	PECy5	Biolegend (305808)
Endoglin (CD105)	Endothelial cells (angiogenisis and pulmonary vascular remodelling; receptor TGFβ)	1:200	PECy7	Biolegend (323217)
Intercellular adhesion molecule-1 (ICAM, CD54)	Endothelial cells (leukocyte adhesion and transmigration)	1:200	AF700	Biolegend (353126)
Melanoma cell adhesion molecule (MCAM, CD146)	Endothelial cells (adhesion and migration, angiogenesis)	1:200	APC-Fire 750	Biolegend (361028)
Fibroblast-specific protein or metastasin 1 (FSP1, S100A4 MTS1) (intracellular)	Fibroblasts (activation and proliferation, interactions macrophages)	1:200	APC	Biolegend (370005)
Alpha-smooth muscle actin (α-SMA) (intracellular)	Myofibroblasts	1:200	AF488	Sigman Aldrich (F3777)

Cluster of differentiation (CD)

Supplementary Table 7: Primary antibodies used for immunohistological staining.

Epitop	Function	Dilution	Host-species	Company (order number)
β-Catenin	Cellular adhesion, EMT, Wnt	1:100	Rabbit	Cell Signaling Technology (8480)
Mucin5AC (MUC5AC)	Goblet cells	1:800	Mouse	Acris (AM50143PU-T)
Zonular occludens protein 1(ZO-1)	Tight junctions	1:100	Rabbit	Cell Signaling (13663)
Acetylated α-tubulin	Ciliated cells	1:800	Mouse	Sigma (T7451)
Pan cytoceratin	Epithelial cells	1:200	Rabbit	Origene/acris (DP010)
FoxJ	Transcription factor ciliogenesis	1:500	Rabbit	Invitrogen (PA-52189)
Vimentin	Mesenchymal cells	1:200	Mouse	Invitrogen (MA5-11883)
CCSP/uteroglobin	Club cells	1:50	Rabbit	Invitrogen (PA5-95864)
TP63	Basal cells	1:50	Mouse	Santa Cruz Biotechnology (sc-25268)
Von-Willebrand-factor (VWF)	Endothelial cells	1:100	Rabbit	Dako (A0082)
Alpha smooth muscle actin (α-SMA)	Smooth muscle cells, myofibroblasts	1:1000	Mouse	Sigma/Merck (A2547)
Collagen I	Collagen submucosa	1:200	Rabbit	OriGene Technologies (R1038)
Collagen IV	Basal membrane	1:100	Mouse	Santa Cruz (sc-59814)
Pan-laminin	Basal membrane	1:500	Rabbit	Thermo Fisher (PA1-16730)
Collagen III	Collagen submucosa	1:50	Mouse	Santa Cruz (sc-271249)
Tenascin C	Wound healing	1:100	Mabbit	Thermo Fisher (600-780)
Fibronectin	Submucosa	1:100	Mouse	Santa Cruz (sc-8422)
Fibroblast specific protein 1 (FSP1/S100A4)	Fibroblasts	1:50	Rabbit	Thermo Fisher (MA5-32347)

Cluster of differentiation (CD)

Supplementary Table 8: Clinical data before implantation of the discovery and replication cohort.

Parameter	Total cohort (n=147)	Grade 0 (n=39)	Grade 1 (n=51)	Grade 2 (n=57)
**Age (years ± SD)**	65.52 ± 7.06	66.08 ± 6.88	65.69 ± 7.58	65.00 ± 6.78
**Sex: male (%)/ female (%)**	59 (40.10) / 88 (59.90)	13 (33.30) / 26 (66.70)	21 (41.2) / 30 (58.8)	25 (43.90) / 32 (56.10)
**BMI**	22.87 ± 3.92	22.46 ± 4.02	22.43 ± 3.45	23.54 ± 4.22
**Packyears (years ± SD)**	39.94 ± 20.81	42.29 ± 23.26	40.61 ± 19.33	37.75 ± 20.49
**Blood leukocytes (cells/nL ± SD)**	8.81 ± 2.44	9.13 ± 2.60	8.31 ± 2.22	9.04 ± 2.51
**Blood eosinophils (cells/µL ± SD)**	168.82 ± 147.08	204.60 ± 196.42	139.00 ± 118.76	170.90 ± 130.40
**Blood neutrophils (cells/µL ± SD)**	6,348.20 ± 2,203.02	6,634.80 ± 2,240.77	5,945.62 ± 1812.22	6,529.10 ± 2476.33
**Blood lymphocytes (cells/µL ± SD)**	1,604.20 ± 641.26	1,695.20 ± 632.75	1,470.80 ± 494.20	1,666.60 ± 747.74
**Blood monocytes (cells/µL ± SD)**	747.10 ± 917.57	1,043.20 ± 1,775.65	653.30 ± 231.91	641.80 ± 223.79
**Blood thrombocytes (cells/µL ± SD)**	275,535.10 ± 82910.03	280996.50 ± 105879.99	261,604.70 ± 69,467.41	283,960.80 ± 77,523.92
**S-keratinin (mg/dl ± SD)**	0.78 ± 0.23	0.85 ± 0.33	0.74 ± 0.16	0.77 ± 0.19
**CRP (± SD)**	2.77 ± 4.16	4.40 ± 6.76	2.70 ± 3.45	1.74 ± 1.56
**FEV1/FVC (% ± SD)**	49.07 ± 17.95	44.11 ± 17.09	50.54 ± 18.81	51.03 ± 17.51
**FEV1 (l ± SD)**	1.26 ± 0.94	1.04 ± 0.72	1.29 ± 1.03	1.38 ± 0.99
**RV/TLC (% ± SD)**	62.34 ± 14.74	65.13 ± 13.05	61.8 ± 16.47	60.99 ± 14.35
**RV (l ± SD)**	4.82 ± 2.03	5.41 ± 2.13	4.63 ± 1.98	4.62 ± 1.98
**VC Max (l ± SD)**	2.67 ± 0.90	5.41 ± 2.13	4.63 ± 1.98	4.62 ± 1.98
**KCO (± SD)**	0.83 ± 0.48	0.74 ± 0.47	0.83 ± 0.50	0.88 ± 0.49
**DLCO [± SD]**	3.86 ± 2.97	3.29 ± 2.41	3.87 ± 3.14	4.26 ± 3.22
**O_2 stress (± SD)**	2.05 ± 1.78	2.18 ± 2.10	2.08 ± 1.70	1.94 ± 1.63
**pH (± SD)**	7.39 ± 0.04	7.40 ± 0.03	7.39 ± 0.04	7.39 ± 0.04
**pCO_2 (± SD)**	43.23 ± 7.95	42.21 ± 10.60	45.30 ± 7.06	42.07 ± 6.20
**pO_2 (± SD)**	81.12 ± 29.11	77.23 ± 20.70	81.34 ± 35.60	83.57 ± 27.84
**O_2 saturation (% ± SD)**	93.98 ± 9.25	94.40 ± 3.09	94.64 ± 2.86	93.11 ± 14.42
**Bacterial colonization (%)**	8.50	6.60	14.00	3.80
**Germ name**		1. *C. albicans*<br>2. *H. influenza*<br>3. *Sprout fungus, P. mirabilis*	1. *E. coli, C. yougae, C. albicans*<br>2. *P. aeruginosa*<br>3. *S. maltophilla*<br>4. *P. aeruginosa*<br>5. *Sprout fungus*<br>6. *C. albicans,*<br>7. *C. koseri*	1. *P. aeruginosa, S. aureus*<br>2. *S. aureus, sprout fungus*
**Distance 6MWT (m ± SD)**	283.78 ± 77.79	269.09 ± 68.03	299.77 ± 58.26	282.94 ± 93.28
**Saturation start 6MWT (% ± SD)**	92.88 ± 4.60	94.37 ± 3.68	91.82 ± 6.63	92.57 ± 3.33
**Saturation minimum 6MWT (% ± SD)**	86.82 ± 4.60	88.85 ± 3.17	85.19 ± 7.68	86.51 ± 5.63
**CAT (± SD)**	24.33 ± 5.27	25.38 ± 5.18	22.00 ± 5.80	27.00 ± 2.65
**Alpha 1 antitrypsin deficiency**	6	0	2	4

Number (n), standard deviation (SD), body mass index (BMI), c-reactive protein (CRP), tiffeneau-index (FEV1/FVC), forced expiratory volume in one second (FEV1), total lung capacity(TLC), residual volume (RV), maximal vital capacity (VC Max), carbon monoxide transfer coefficient (KCO), diffusing capacity of the lungs for carbon monoxide (DLCO), potential hydrogen (pH), partial pressure of carbon dioxide (pCO_2), partial pressure of oxygen (pO_2), 6-minute walk test (6MWT), COPD assessment test (CAT).

Supplementary Table 9: Retrospective analysis of clinical data at follow up scoring of the discovery and replication.

Parameter	Total cohort	Grade 0	Grade 1	Grade 2
**Dysfunction (%)****	16.03	0.00	9.80	43.30
**Dislocation (%)****	15.60	0.00	11.80	29.80
**Revision (%)****	19.70	0.00	13.70	38.60
**Lung lobe (%)**<br>ROL<br>RUL<br>LOL<br>LUL<br>ML	 6.10<br>17.00<br>35.40<br>39.50<br>2.00	 5.10<br>12.80<br>38.50<br>38.50<br>5.10	 7.80<br>7.80<br>41.20<br>41.20<br>2.00	 5.30<br>28.10<br>28.10<br>38.60<br>0.00
**Month since implantation**	24.78 ± 27.98	23.90 ± 24.98	25.65 ± 35.46	24.60 ± 22.21
**Blood leukocytes (cells/nL ± SD)**	9.16 ± 3.18	8.85 ± 2.65	8.96 ± 2.59	9.53 ± 3.89
**Blood eosinophils (cells/µL ± SD)**	161.90 ± 220.28	165.00 ± 180.31	155.10 ± 203.45	165.80 ± 262.90
**Blood neutrophils(cells/µL ± SD)**	7,741.10 ± 9,077.09	10,455.30 ± 16,447.42	6,663.80 ± 2,467.43	6,693.60 ± 3,894.09
**Blood lymphocytes (cells/µL ± SD)**	1,595.10 ± 918.99	1,672.50 ± 1,473.85	1,509.10 ± 262.89	1,617.70 ± 642.61
**Blood monocytes (cells/µL ± SD)**	682.50 ± 349.41	715.00 ± 359.46	647.10 ± 262.89	691.30 ±411.68
**Blood thrombocytes (cells/µL ± SD)**	288,808.00 ± 90,483.10	290,000.00 ± 75,308.890	286,708.30 ± 82,260.60	289,879.60 ± 106,822.06
**S-keratinin [(mg/dl± SD)**	0.80 ± 0.21	0.82 ± 0.20	0.79 ± 0.26	0.80 ± 0.17
**CRP (± SD)**	2.38 ± 4.92	1.32 ± 1.77	2.33 ± 3.18	3.04 ± 6.95
**FEV1/FVC (% ± SD)**	38.89 ± 10.39	38.73 ± 10.72	39.26 ± 10.44	38.69 ± 10.32
**FEV1 (l ± SD)**	0.82 ± 0.40	0.84 ± 0.34	0.80 ± 0.46	0.82 ± 0.40
**RV/TLC (% ± SD)**	67.65 ± 9.30	67.40 ± 7.69	68.78 ± 10.33	66.82 ± 9.39
**RV (l ± SD)**	5.27 ± 1.56	5.47 ± 1.71	5.36 ± 1.65	5.07 ± 1.38
**VC Max (± SD)**	2.44 ± 0.72	2.54 ± 0.67	2.32 ± 0.67	2.49 ± 0.78
**KCO (± SD)**	0.48 ± 0.72	0.56 ± 0.23	0.36 ± 0.18	0.52 ± 0.16
**DLCO (± SD)**	1.92 ± 0.96	2.29 ± 1.31	1.48 ± 0.82	0.52 ± 0.16
**O_2 stress (± SD)**	2.46 ± 1.74	2.94 ± 1.69	2.32 ± 1.76	2.31 ± 1.75
**pH (± SD)**	7.35 ± 0.60	7.41 ± 0.05	7.25 ± 1.02	7.41 ± 0.06
**pCO_2 (± SD)**	43.01 ± 8.73	41.44 ± 8.37	44.78 ± 8.89	42.49 ± 8.71
**pO_2 (± SD)**	77.19 ± 26.01	72.62 ± 20.18	81.31 ± 34.37	76.51 ± 20.19
**O_2 saturation (% ± SD)**	94.94 ± 2.28	94.36 ± 2.62	95.12 ± 2.18	95.15 ± 2.10
**Bacterial colonization (%)**	22.40	18.90	26.00	21.40
**Germ name**		1. *A. spp,*<br>*E. cloacae*<br>2. *K. oxytoca,*<br>*S. marcescense*<br>3. *S. marcescense,*<br>*P. aeruginosa*<br>4. *S. marcescense*<br>5. *P. aeruginosa*<br>6. *P. aeroginosa,*<br>*A. xylosoxidans*	1. *S. marcescense*<br>2. *S. aureus*<br>3. *E. coli,*<br>*C. albicans*<br>4. *P. aeroginosa*<br>5. *H. influenza.*<br>*C. albicans*<br>6. *S. aureus*<br>7. *E. aerogenes,*<br>*E. coli (3MRGN)*<br>8. *P. aeruginosa*<br>9. *S. pneumonia,*<br>*sprout fungus*<br>10. *Sprout fungus*<br>11. *K. aerogenes,*<br>*C. koseri,*<br>*P. vulgaris,*<br>*K. pneumoniae,*<br>*sprout fungus*	1. *S. aureus,*<br>*C. konseri*<br>2. *S. aureus*<br>3. *N. sicca,*<br>*P. buccae,*<br>*S. vestibularis,*<br>*C. albicans*<br>4. *S. aureus,*<br>*P. aeruginosa*<br>5. *K. pneumonia,*<br>*S. aureus*<br>6. *P. aeruginosa,*<br>*S. marcescense*<br>7. *M. catarrhalis*<br>8. *K. oxytoca,*<br>*S. aureus*<br>9. *E. coli (3MRGN)*<br>10. *E. coli (3MRGN),*<br>*P. aeruginosa*<br>11. *E. coli,*<br>*C. albicans*
**Distance 6MWT (m ± SD)**	303.91 ± 99.81	277.90 ± 92.65	308.00 ± 99.52	319.26 ± 103.86
**Saturation start 6MWT (%± SD)**	92.87 ± 3.54	92.93 ± 2.82	93.04 ± 3.38	92.66 ± 4.19

**Saturation minimum 6MWT (%)**	86.67 ± 4.55	87.36 ± 3.87	87.53 ± 4.31	85.34 ± 5.00
**CAT (± SD)**	26.27 ± 10.30	31.00 ± 9.90	28.00 ± 4.43	23.43± 6.60

Number (n), standard deviation (SD), body mass index (BMI), c-reactive protein (CRP), tiffeneau-index (FEV1/FVC), forced expiratory volume in one second (FEV1), total lung capacity(TLC), residual volume (RV), maximal vital capacity (VC Max), carbon monoxide transfer coefficient (KCO), diffusing capacity of the lungs for carbon monoxide (DLCO), potential hydrogen (pH), partial pressure of carbon dioxide (pCO_2), partial pressure of oxygen (pO_2), 6-minute walk test (6MWT), COPD assessment test (CAT). Correlations between parameters and increasing severity grades were performed by Spearman correlation analyses. Only significant correlations are marked. ** $p < 0.001$.

Supplementary Table 10: Clinical data of the biopsy patients before implantation of the valve.

**Parameter**	**Total cohort** (n=37)	**Grade 0** (n=11)	**Grade 1** (n=14)	**Grade 2** (n=12)
**Age (years)**	64.59 ± 8.52	62.36 ± 11.18	66.50 ± 8.86	64.42 ± 4.64
**Sex: male (%)/ female (%)**	19 (51,35)/18 (48,65)	4 (36,36) /7 (63,64)	9 (64,29) / 5 (35,71)	6 (50,00) / 6 (50,00)
**Packyears (years ± SD)**	36.33 ± 21.28	30.73 ± 18.33	43.31 ± 21.25	33.92 ± 23.40
**Blood leukocytes (cells/nL ± SD)**	8.62 ± 2.57	8.29 ± 1.96	9.04 ± 3.06	8.34 ± 2.47
**Blood eosinophils (cells/µL ± SD)**	160.86 ± 137.14	177.27 ± 111.81	129.29 ± 96.91	187.00 ± 202.38
**Blood neutrophils (cells/µL ± SD)**	5,792.94 ± 353.66	5,057.00 ± 1,235.13	6,435.00 ± 3,021.62	5,630.00 ± 2,079.95
**Blood lymphocytes (cells/µL ± SD)****	1,653.71 ± 644.38	1,946.36 ± 798.41	1,642.86 ± 557.67	1,347.00 ± 449.15
**Blood Monocytes (cells/µL ± SD)**	660.00 ± 177.17	588.18 ± 111.52	665.71 ± 164.91	731.00 ± 231.35
**Blood thrombocytes (cells/µL ± SD)**	267,558.82 ± 103,879.648	2,616,000.000 ± 62,512.58	294,428.57 ± 142,822.49	235,900.00 ± 62,600.05
**S-keratinin (mg/dl)**	0.83 ± 0.21	0.79 ± 0.20	0.87 ± 0.20	0.84 ± 0.25
**CRP (± SD)**	2.23 ± 2.00	1.92 ± 1.93	2.30 ± 2.22	3.00 ± 2.89
**FEV1/FVC (% ± SD)**	37.97 ± 7.64	36.62 ± 3.53	37.11 ± 9.31	39.46 ± 9.06
**FEV1 (l ± SD)**	1.16 ± 1.63	0.86 ± 0.17	0.97 ± 0.7	1.75 ± 3.01
**RV/TLC (%± SD)**	71.82 ± 30.67	65.20 ± 4.82	67.91 ± 8.81	85.20 ± 56.86
**RV (l ± SD)**	5.33 ± 1.46	5.25 ± 0.83	5.93 ± 1.80	4.51 ± 1.31
**VC Max (± SD)**	2.75 ± 0.82	2.81 ± 0.57	2.79 ± 1.06	2.57 ± 0.84
**KCO (± SD)**	0.54 ± 0.20	0.51 ± 0.21	0.49 ± 0.11	0.66 ± 0.28
**DLCO (± SD)**	4.51 ± 7.78	2.34 ± 1.09	5.07 ± 9.46	6.82 ± 10.59
**O_2 stress (% ± SD)**	1.86 ± 1.56	1.83 ± 1.72	1.57 ± 1.51	2.14 ± 1.77
**pH (± SD)**	7.41 ± 0.03	7.40 ± 0.02	7.42 ± 0.29	7.41 ± 0.26
**pCO_2 (± SD)**	41.36 ± 5.01	40.98 ± 5.80	41.28 ± 4.83	42.06 ± 5.02
**pO_2 (± SD)**	74.62 ± 20.26	84.51 ± 31.50	67.65 ± 10.93	71.27 ± 8.88
**O_2 saturation (% ± SD)**	92.70 ± 10.20	95.50 ± 1.91	88.25 ± 17.08	94.10 ± 2.50
**Bacterial colonization**	3	1	2	0
**Germ name**		1. *S aureus*	1. *S. aureus*<br>2. *M. chimeara*	
**Distance 6MWT (m ± SD)**	331.21 ± 97.34	258.64 ± 96.62	318.64 ± 118.75	314.90 ± 79.01
**Saturation start 6MWT (%± SD)**	93.58 ± 3.63	94.15 ± 1.83	93.01 ± 5.69	93.20 ± 2.68
**Saturation minimum 6MWT (%± SD)**	85.13 ± 6.11	84.46 ± 9.21	85.10 ± 3.07	86.10 ± 4.77
**CAT (± SD)**	23.73 ± 6.58	24.78 ± 3.93	23.17 ± 9.83	22.83 ± 7.55
**Alpha 1 antitrypsin deficiency**	2	1	1	0
**Early complication (< 4 weeks)**	Pneumothorax with drainage <7 days: 1<br>Pneumothorax with valve removal: 2<br>Pneumothorax with surgery: 1	Pneumothorax with valve removal: 1<br>Pneumothorax with surgery: 1	Pneumothorax with valve removal: 1	Pneumothorax with drainage <7 days: 1

Number (n), standard deviation (SD), body mass index (BMI), c-reactive protein (CRP), tiffeneau-index (FEV1/FVC), forced expiratory volume in one second (FEV1), total lung capacity(TLC), residual volume (RV), maximal vital capacity (VC Max), carbon monoxide transfer coefficient (KCO), diffusing capacity of the lungs for carbon monoxide (DLCO), potential hydrogen (pH), partial pressure of carbon dioxide (pCO_2), partial pressure of oxygen (pO_2), 6-minute walk test (6MWT), COPD assessment test (CAT). Correlations between all clinical parameters and increasing severity grades were performed by Spearman correlation analyses. Only significant correlations are marked.** $p < 0.001$.

Supplementary Table 11: Clinical data of biopsy patients at date of biopsy.

Parameter	Total cohort	Grade 0	Grade 1	Grade 2
**Blood leukocytes (cell/nL ± SD)**	7.55 ± 2.20	6.66 ± 2.31	8.35 ± 2.06	7.34 ± 2.15
**Blood eosinophils (cells/µL ± SD)**	198.24 ± 204.78	190.00 ± 155.16	170.00 ± 222.19	235.00 ± 227.98
**Blood neutrophils (cells/µL ± SD)**	7,640.59 ± 10,275.02	5,913.33 ± 2,068.01	10,812.31 ± 16,277.64	5,500.00 ± 2,289.12
**Blood lymphocytes (cells/µL ± SD)****	2,172.06 ± 4,048.37	1,711.11 ± 498.46	3,223.08 ± 6,538.344	1,379.17 ± 431.03
**Blood monocytes (cells/µL ± SD)**	637.94 ± 150.83	588.89 ± 119.94	626.15 ± 128.49	687.50 ± 186.99
**Blood thrombocytes (cells/µL ± SD)**	267,294.12 ± 65,326.00	259,666.67 ± 78,281.54	276,000.00 ± 67,265.64	263,583.33 ± 57,074.85
**S-keratinin (mg/dl)****	0.81 ± 0.19	0.77 ± 0.14	0.79 ± 0.17	0.86 ± 0.24
**CRP (± SD)**	1.75 ± 1.19	1.43 ± 1.11	1.87 ± 1.94	2.07 ± 0.64
**FEV1/FVC (% ± SD)**	38.50 ± 8.71	36.18 ± 4.58	39.41 ± 10.02	40.16 ± 9.52
**FEV1 (l ± SD)**	0.85 ± 0.24	0.82 ± 0.19	0.81 ± 0.26	0.92 ± 0.28
**RV/TLC (%± SD)**	69.82 ± 25.34	63.91 ± 10.52	66.56 ± 6.04	78.68 ± 41.63
**RV (l ± SD)**	5.05 ± 0.95	4.99 ± 1.01	5.08 ± 1.20	5.02 ± 0.85
**VC Max (± SD)**	2.69 ± 0.81	2.81 ± 0.91	2.57 ± 0.84	2.62 ± 0.69
**KCO (± SD)**	0.56 ± 0.19	0.54 ± 0.07	0.47 ± 0.18	0.68 ± 0.18
**DLCO (± SD)**	3.65 ± 5.23	2.67 ± 0.76	4.86 ± 7.91	2.70 ± 0.94
**O_2 stress (% ± SD)**	2.63 ± 1.89	2.83 ± 2.86	2.57 ± 1.51	2.50 ± 1.38
**pH (± SD)**	7.42 ± 0.05	7.39 ± 0.04	7.43 ± 0.35	7.44 ± 0.06
**pCO_2 (± SD)**	39.89 ± 5.21	39.91 ± 5.08	40.40 ± 4.37	39.26 ± 6.53
**pO_2 (± SD)**	74.41 ± 15.01	79.86 ± 13.08	71.68 ± 13.10	75.08 ± 17.67
**O_2 saturation [% ± SD]**	95.07 ± 2.18	95.70 ± 1.66	94.94 ± 2.04	95.13 ± 2.47
**Bacterial colonization****	12	1	5	6
**Germ name**		1. *S. aureus*	1. *E. coli*<br>2. *S. aureus, N. meningitidis*<br>3. *S. aureus*<br>4. *S. nematodiphila, A. fumigatius*<br>5. *P. aeruginosa*	1. *K. pneumonia*<br>2. *A. fumigatus*<br>3. *P. aeruginosa, C. albicans*<br>4. *A. fumigatius*<br>5. *E. coli*<br>6. *P. aeruginosa*
**Distance 6MWT (m ± SD)**	308.60 ± 93.92	328.75 ± 116.67	306.25 ± 80.58	295.30 ± 96.63
**Saturation start 6MWT (% ± SD)**	93.34 ± 3.80	94.71 ± 2.73	92.52 ± 4.50	93.15 ± 3.75
**Saturation minimum 6MWT (% ± SD)**	86.00 ± 4.17	85.75 ± 3.29	84.64 ± 3.70	87.70 ± 4.99

Number (n), standard deviation (SD), body mass index (BMI), c-reactive protein (CRP), tiffeneau-index (FEV1/FVC), forced expiratory volume in one second (FEV1), total lung capacity(TLC), residual volume (RV), maximal vital capacity (VC Max), carbon monoxide transfer coefficient (KCO), diffusing capacity of the lungs for carbon monoxide (DLCO), potential hydrogen (pH), partial pressure of carbon dioxide (pCO_2), partial pressure of oxygen (pO_2), 6-minute walk test (6MWT), COPD assessment test (CAT). Correlations between all clinical parameters and increasing severity grades were performed by Spearman correlation analyses. Only significant correlation are marked as ** at $p < 0.001$.

Supplementary Table 12: Clinical data of stent patients before implantation.

**Parameter**	**Stent patients**<br>(n=11)
**Age (years)**	62.91 ± 12.87
**Sex**	m= 3; w= 8
**Packyears (years)**	ND
**Diagnosis**	1. Skolisis; airway deformation trachea & inter-bronchius<br>2. Polichondritis<br>3. Main bronchus stenosis (after long-term ventilation)<br>4. Persistent esophagotracheal fistula<br>5. Esophagotracheal fistula + high-grade scarred esophageal stenosis<br>6. Granulomatous polyangiitis GPA<br>7. Exophytic tumour growth, subtotal stenosis<br>8. Carcinoma<br>9. Distal tracheal stenosis after long-term ventilation<br>10. ANCA vasculitis; subglottic stenosis<br>11. Small cell lung carcinoma SCLC left central
**Blood leukocytes (cells/nL ± SD)**	9.84 ± 4.16
**Blood eosinophils (cells/µL ± SD)**	220.00 ± 439.64
**Blood neutrophils (cells/µL ± SD)**	8,092.50 ± 4,385.21
**Blood lymphocytes (cells/µL ± SD)**	1,343.75 ± 679.85
**Blood monocytes (cells/µL ± SD)**	625.00 ± 314.55
**Blood thrombocytes(cells/µL ± SD)**	289,000.00 ± 147,924.812
**S-keratinin (mg/dl ± SD)**	1.05 ± 0.36
**CRP (± SD)**	5.27 ± 6.47

Number (n), standard deviation (SD), not determined (ND), c-reactive protein (CRP).

Supplementary Table 13: Clinical data of stent patients at biopsy.

**Parameter**	**Stent patients**
**Blood leukocytes (cells/nL ± SD)**	8.76 ± 4.34
**Blood eosinophils (cells/µL ± SD)**	104.29 ± 65.64
**Blood neutrophils (cells/µL ± SD)**	6,905.71 ± 4,547.67
**Blood lymphocytes (cells/µL ± SD)**	1,191.86 ± 269.36
**Blood monocytes (cells/µL ± SD)**	548.57 ± 275.65
**Blood thrombocytes (cells/µL ± SD)**	260,250.00 ± 128,057.74
**S-keratinin (mg/dl ± SD)**	1.00 ± 0.57
**CRP (± SD)**	1.6 ± 0.57
**Bacterial colonization**	8
**Germ name**	1. *Asperagillus fumigatus*<br>2. *Candida albicans, Stenotrophomonas malthophilia*<br>3. *Pseudomonas aeruginosa, Serratia marcescens, Proteus mirabilis, fungal infection, Candida albicans*<br>4. *Escherichia coli*<br>5. *Staphylococcus aureus*<br>6. *Pseudomonas aeruginosa*<br>7. *Enterobacter cloacae complex*<br>8. *Serratia marcescens, Streptococcus dysgalactiae*<br>9. *Pseudomonas aeruginosa, Haemophilus influenzae, Staphylococcus aureus*<br>10. *Proteus mirabilis, Escherichia coli*

Number (n), standard deviation (SD), c-reactive protein (CRP).

Abbreviations

µL	Micro liter
6MWT	6-minute walk test
BMI	Body mass index
CAT	COPD assessment test
CCSP	Clara cell secretory protein
CD	Cluster of differentiation
COPD	Chronic obstructive pulmonary disease
CRP	C-reactive protein
DAMPs	Damage-associated molecular patterns
DC	Dendritic cell
DLCO	Diffusing capacity of the lungs for carbon monoxide
EBVs	Endobronchial valves
ECM	Extracellular matrix
FEV1	Forced expiratory volume in one second
FEV1/FVC	Tiffeneau-index
FoxJ	Forkhead box protein J
FSP1	Fibroblast specific protein 1
FVC	Forced expiratory vital capacity
HLADR	Human leukocyte antigen – DR isotype
ICAM-1	Intercellular adhesion molecule 1
KCO	Carbon monoxide transfer coefficient
M1	Classically activated/pro-inflammatory macrophages
M2	Alternative activated/anti-inflammatory macrophages
MCAM	Melanoma cell adhesion molecule
MMP	Matrix metalloproteinase
MUC5AC	Mucin 5AC
NK	Natural killer cells
NKT	Natural killer T cell
nL	Nano liter
PAS	Periodic acid Schiff's reaction
pCO_2	Partial pressure of carbon dioxide
PFA	Paraformaldehyde
pH	Potential hydrogen
pO_2	Partial pressure of oxygen
RV	Residual volume
TEMRA	Terminal differentiated T cells
Th	T helper cell
TLC	Total lung capacity
TP63	Tumour protein p63
Treg	Regulatory T cells
Vc Max	Maximal vital capacity
V-CAM-1	Vascular cell adhesion protein 1
VE-Cadherin	Vascular endothelial cadherin
VEGFR-2	Vascular endothelial growth factor receptor 2
VWF	Von Willebrand factor
Z0-1	Zonula occludens protein 1
αSMA	Alpha smooth muscle actin
α-Tubulin	Alpha tubulin

368	β-Catenin	beta-catenin

References

1. Westhölter D, Beckert H, Straßburg S, Wiewrodt R, Koczulla AR, Greulich T, et al. Pseudomonas aeruginosa infection, but not mono or dual-combination CFTR modulator therapy, affects circulating regulatory T cells in an adult population with cystic fibrosis. J Cyst Fibros. 2021;20(6):1072–9. doi:10.1016/j.jcf.2021.05.001
2. Rühle PF, Fietkau R, Gaipl US, Frey B. Development of a modular assay for detailed immunophenotyping of peripheral human whole blood samples by multicolor flow cytometry. Int J Mol Sci. 2016;17(8):1316. doi:10.3390/ijms17081316
3. Jimenez Vera E, Chew YV, Nicholson L, Peakman M, Tree TIM. Standardisation of flow cytometry for whole blood immunophenotyping of islet transplant and transplant clinical trial recipients. PLoS One. 2019;14(5):e0217163. doi:10.1371/journal.pone.0217163
4. Schuler M, Cuppens K, Plönes T, Kirchner M, Kühle G, Stauber R, et al. Neoadjuvant nivolumab with or without relatlimab in resectable non-small-cell lung cancer: a randomized phase 2 trial. Nat Med. 2024;30(6):1602–11. doi:10.1038/s41591-024-02965-0
5. Bharat A, Bhorade SM, Morales-Nebreda L, McQuattie-Pimentel AC, Soberanes S, Ridge KM, et al. Flow cytometry reveals similarities between lung macrophages in humans and mice. Am J Respir Cell Mol Biol. 2016;54(1):147–9. doi:10.1165/rcmb.2015-0147LE
6. Bonser LR, Koh KD, Johansson K, Zlock L, Zhou X, Erle DJ. Flow-cytometric analysis and purification of airway epithelial-cell subsets. Am J Respir Cell Mol Biol. 2021;64(3):308–17. doi:10.1165/rcmb.2020-0149MA
